# Prenatal phthalate exposure and adverse birth outcomes in the USA: a prospective analysis of births and estimates of attributable burden and costs

**DOI:** 10.1016/S2542-5196(23)00270-X

**Published:** 2024-02

**Authors:** Leonardo Trasande, Morgan E Nelson, Akram Alshawabkeh, Emily S Barrett, Jessie P Buckley, Dana Dabelea, Anne L Dunlop, Julie B Herbstman, John D Meeker, Mrudula Naidu, Craig Newschaffer, Amy M Padula, Megan E Romano, Douglas M Ruden, Sheela Sathyanarayana, Susan L Schantz, Anne P Starling, Ghassan B Hamra

**Affiliations:** Department of Pediatrics, Division of Environmental Pediatrics, New York University Grossman School of Medicine, New York, NY, USA; Department of Population Health, New York University Grossman School of Medicine, New York, NY, USA; New York University Wagner School of Public Service, New York, NY, USA; RTI International, Research Triangle Park, Raleigh, NC, USA; Northeastern University, Boston, MA, USA; Department of Biostatistics and Epidemiology, Rutgers University School of Public Health, Piscataway, NJ, USA; Department of Epidemiology, Gillings School of Global Public Health, University of North Carolina at Chapel Hill, Chapel Hill, NC, USA; Department of Environmental Health and Engineering; Department of Epidemiology; Johns Hopkins Bloomberg School of Public Health, Baltimore, MD, USA;Lifecourse Epidemiology Adiposity and Diabetes Center, University of Colorado Anschutz Medical Campus, Aurora, CO, USA; Department of Gynecology and Obstetrics, Emory University School of Medicine, Atlanta, GA, USA; Department of Environmental Health Sciences, Columbia University Mailman School of Public Health, New York, NY, USA; Department of Environmental Health Sciences, University of Michigan School of Public Health, Ann Arbor, MI, USA; Department of Pediatrics, Division of Environmental Pediatrics, New York University Grossman School of Medicine, New York, NY, USA; College of Human Health and Development, Penn State University, Hershey, PA, USA; Department of Obstetrics, Gynecology and Reproductive Sciences, University of California, San Francisco, San Francisco, CA, USA; Department of Epidemiology, Geisel School of Medicine at Dartmouth, Lebanon, NH, USA; Department of Obstetrics and Gynecology, Wayne State University, Detroit, MI, USA; Seattle Children’s Research Institute, Seattle, WA, USA; Department of Pediatrics, University of Washington, Seattle, WA, USA; Beckman Institute for Advanced Science and Technology, University of Illinois at Urbana-Champaign, Urbana, IL, USA; Department of Epidemiology, Gillings School of Global Public Health, University of North Carolina at Chapel Hill, Chapel Hill, NC, USA; Johns Hopkins Bloomberg School of Public Health, Baltimore, MD, USA;Lifecourse Epidemiology Adiposity and Diabetes Center, University of Colorado Anschutz Medical Campus, Aurora, CO, USA; Department of Environmental Health and Engineering; Department of Epidemiology

## Abstract

**Background:**

Phthalates are synthetic chemicals widely used in consumer products and have been identified to contribute to preterm birth. Existing studies have methodological limitations and potential effects of di-2-ethylhexyl phthalate (DEHP) replacements are poorly characterised. Attributable fractions and costs have not been quantified, limiting the ability to weigh trade-offs involved in ongoing use. We aimed to leverage a large, diverse US cohort to study associations of phthalate metabolites with birthweight and gestational age, and estimate attributable adverse birth outcomes and associated costs.

**Methods:**

In this prospective analysis we used extant data in the US National Institutes of Health Environmental influences on Child Health Outcomes (ECHO) Program from 1998 to 2022 to study associations of 20 phthalate metabolites with gestational age at birth, birthweight, birth length, and birthweight for gestational age z-scores. We also estimated attributable adverse birth outcomes and associated costs. Mother–child dyads were included in the study if there were one or more urinary phthalate measurements during the index pregnancy; data on child’s gestational age and birthweight; and singleton delivery.

**Findings:**

We identified 5006 mother–child dyads from 13 cohorts in the ECHO Program. Phthalic acid, diisodecyl phthalate (DiDP), di-n-octyl phthalate (DnOP), and diisononyl phthalate (DiNP) were most strongly associated with gestational age, birth length, and birthweight, especially compared with DEHP or other metabolite groupings. Although DEHP was associated with preterm birth (odds ratio 1·45 [95% CI 1·05–2·01]), the risks per log_10_ increase were higher for phthalic acid (2·71 [1·91–3·83]), DiNP (2·25 [1·67–3·00]), DiDP (1·69 [1·25–2·28]), and DnOP (2·90 [1·96–4·23]). We estimated 56 595 (sensitivity analyses 24 003–120 116) phthalate-attributable preterm birth cases in 2018 with associated costs of US$3·84 billion (sensitivity analysis 1·63– 8·14 billion).

**Interpretation:**

In a large, diverse sample of US births, exposure to DEHP, DiDP, DiNP, and DnOP were associated with decreased gestational age and increased risk of preterm birth, suggesting substantial opportunities for prevention. This finding suggests the adverse consequences of substitution of DEHP with chemically similar phthalates and need to regulate chemicals with similar properties as a class.

## Introduction

The consequences of shortened gestation and intrauterine growth restriction are profound, including infant and childhood mortality;^1,2^ adverse psychological, behavioural, and educational outcomes in young adulthood;^3,4^ and cardiovascular disease and diabetes in later life.^5–7^ In the USA, low birthweight occurred among 8·2% of live births and preterm birth occurred among 10·1% of live births in 2020, failing to achieve Healthy People 2020 goals of 7·8% and 9·4%, respectively.^8^

Low birthweight, preterm birth, and other adverse birth outcomes (small for gestational age and large for gestational age) are highly multifactorial with risk factors including maternal age, inadequate prenatal care, low socioeconomic status, and pre-eclampsia, most of which are not easily amenable to modification or avoidance. Increasingly, synthetic chemicals are being recognised for potential independent contributions.^9^ One class of synthetic chemicals, phthalates, which are used in personal care products and food packaging, induce inflammation and oxidative stress, and are endocrine disruptors, with varying degrees of estrogenic and anti-androgenic effects.^9^ Moreover, these pathways interact; inflammation can influence hormonal regulation in pregnancy.^10^ Inflammation and oxidative stress can induce endothelial activation common in pre-clampsia, and oxidative stress can induce placental insufficiency as well as pre-clampsia and premature rupture of membranes.^11^

Multiple cohort studies, systematic reviews, and meta-analyses have identified largely consistent relationships of prenatal phthalate exposures with decreased gestational age and preterm birth.^12–31^ Some limitations in interpretation include imprecision due to the use of only one to two spot urine samples in pregnancy to evaluate exposure, the short half-life of most phthalates, and the relatively modest number of studies which have examined effects in Hispanic, Latino, or other minority populations.^32^ Addition ally, the potential effect of replacements of di-2-ethylhexyl phthalate (DEHP) are less well characterised. The attributable fraction of adverse birth outcomes due to these chemical exposures has not been quantified, limiting the ability to weigh the societal trade-offs involved in the ongoing use of these chemicals of concern.

The National Institutes of Health Environmental influences on Child Health Outcomes (ECHO) Program unites existing paediatric cohorts from across the USA in a common, harmonised, and prospective protocol to identify environmental and preventable origins of low birthweight, preterm birth, and other effects on child health and development. We aimed to leverage this large, diverse ECHO cohort to study the effects of prenatal phthalate exposure on birthweight and gestational age, and to estimate attributable adverse birth outcomes and associated health and other costs to society.

## Methods

### Study design

In this prospective analysis we used data from the ECHO cohort study, which includes 69 unique cohorts, to improve understanding of the effect of environmental insults on children’s health. Existing data are harmonised to facilitate pooled analyses, and new data are collected using a common, standardised protocol.^33^ Eligibility criteria for mother–child dyads from the ECHO Program to be included in the current analysis were: one or more urinary phthalate measurements during the index pregnancy; data on child’s gestational age and birthweight; and singleton delivery.

The Western IRB-Copernicus Group serves as the Central Institutional Review Board for the ECHO Program, ensuring that all participants provided appropriate consent.

### Measurement of urinary phthalate metabolites

Phthalate metabolites in maternal urine were measured at the Centers for Disease Control and Prevention^34^ and Human Health Exposure Analysis Resource laboratories.^35–37^ To be considered, we required that a phthalate was detectable in more than 50% of samples and that at least 1000 participants had a detectable sample. We summed phthalates into groups on the basis of known similarities in chemical structure, following previous practices^38^ in which classes were interrogated as a screen for associations of significance, and then examining drivers of the significant classes. This approach has the effect of reducing comparison number (and concerns about false positives). Specifically, we summed low molecular weight (LMW) phthalate, high molecular weight (HMW) phthalate, and DEHP, di-n-octyl phthalate (DnOP), and diisononyl phthalate (DiNP) metabolites into groups. If a cohort was missing any phthalate that contributed to a grouping, it was excluded from analyses for that phthalate group, but not necessarily other phthalate groups. We included phthalates in summed groups that were detected in at least 50% of the eligible participants; all analytes analysed were more than 70% above the limit of detection across all the cohorts. The LMW group includes mono-ethyl phthalate, mono-n-butyl phthalate, and mono-isobutyl phthalate. The HMW group includes mono(3-carboxylpropyl) phthalate (MCPP), mono(2-ethyl-5-carboxypentyl) phthalate (MECPP), mono(2-ethyl-5-hydroxyhexyl) phthalate (MEHHP), mono(2-ethyl-5-oxohexyl) phthalate (MEOHP), mono-2-ethylhexyl phthalate (MEHP), and monobenzyl phthalate. We summed DEHP metabolites MEHP, MEHHP, MEOHP, and MECPP into a third group. We summed MCPP and mono(7-carboxyheptly) phthalate to represent DnOP and mono-isononyl phthalate (MiNP) and mono-carboxy isocytyl phthalate (MCOP) to represent DiNP. Finally, we use mono-carboxy isononyl phthalate as a proxy for diisodecyl phthalate (DiDP). All sums are based on molecular weights and the formulas are available in the appendix (p 10). Summed groups were log_10_ transformed before analysis. Phthalic acid was treated separately as it is known to be an end metabolite for all the previously mentioned phthalates.

Before summing into groups, we first replaced any values that were below the lower limit of detection with the lower limit of detection divided by the square root of 2. We then adjusted for urinary dilution by use of either creatinine or specific gravity, depending on availability of each from cohorts. We used the Boeninger method to standardise phthalate biomarkers by cohort-specific median creatinine or specific gravity value, which has been shown to be valid previously.^39^ We finally converted all measurements to nmol/L. We took the mean of repeated measures within a trimester and trimester specific measures or means were later meaned to pregnancy-average values. We decided not to test mixtures of chemicals in the present study, because the correlations were not high.

### Outcomes

Our continuous outcomes of interest were gestational age at birth (completed weeks), birthweight (g), birth length (cm), and birthweight for gestational age z-scores; the latter were standardised using child sex at birth and birth parent’s parity.^40^ We also considered dichotomous outcomes including preterm birth (birth <37 weeks *vs* ≥37 weeks), small for gestational age and large for gestational age (based on the lower and upper tenth percentiles of z-score standardised birthweight for gestational age estimated from a US reference population^40^), low birthweight (<2500 g *vs* ≥2500 g), and low birthweight among preterm and term births.

### Statistical analysis

We adjusted all models for a priori theorised confounders. These confounders included maternal age at delivery (continuous years), maternal race and ethnicity (non-Hispanic White, non-Hispanic Black, Hispanic or Latino, other or unknown), maternal education (High School Degree or General Education Diploma or less, and some college and higher), parity (0, 1, and ≥2), and child sex at birth (male *vs* female). In models considering birthweight for gestational age z-scores as the outcome, we excluded child sex and parity, but included them in supplementary analyses.

Our main analyses considered LMW, HMW, DEHP, phthalic acid, DiNP, DnOP, and DiDP as primary phthalate metabolites of interest. In all analyses we used linear mixed effects models and included cohort as a random effect term to account for baseline differences across cohorts. For continuous outcomes, we treated the outcome as normally distributed, and for dichotomous outcomes we applied a logistic regression framework; because outcomes are all less than 10% of the total sample, we interpret odds ratios as risk ratios (RRs). All models were adjusted for the covariates listed earlier. Tobacco exposure was also substantially missing in the sample, and because it was low prevalence, we kept tobacco as a confounder in sensitivity analyses. Because phthalates were available at different timepoints in pregnancy and, for some mothers, at multiple timepoints during pregnancy, we explored trimester-specific effects of phthalates in addition to estimating effects of pregnancy-averaged phthalate metabolites.

In addition to primary analyses, we conducted exploratory analyses to explore modifying effects of covariates, to evaluate if effects were different in subgroups. Specifically, we estimated associations of metabolites on birth outcomes within strata of child biological sex at birth (male *vs* female), maternal education (high school *vs* some college or greater), parity (0, 1, or ≥2 previous children), and maternal race or ethnicity (non-Hispanic White, non-Hispanic Black, or Hispanic or Latino). We considered whether tobacco use during pregnancy (yes or no) additionally confounded the relationship of phthalate metabolites with birth outcomes. We also considered the relationship of phthalates with a more granular categorisation of gestational age: preterm (<37·0 weeks), early term (≥37·0 to <39·0 weeks), and late term (≥41·0 weeks) versus term (≥39·0 to <41·0 weeks) as the reference group. We also considered a birthweight for gestational age z-scores that were not based on parity, but only sex at birth. We conducted leave-one-out analyses to determine if the main findings were driven by results from a single cohort. We added pre-pregnancy BMI to full models and assessed the effects on regression coefficients. Finally, recognising that phthalates can contribute to gestational diabetes and hypertensive disorders that might mediate or moderate observed associations, we separately added gestational diabetes and hypertensive disorders to full models and assessed the effects on regression coefficients.

All statistical analyses were conducted in SAS statistical software.

### Calculations of phthalate-attributable preterm birth and associated costs

We focused attribution of adverse birth outcomes on preterm birth due to the consistency and strength of relationships observed in our results from regression analyses for this outcome. The ECHO data span a broad time period, as early as 1998, and more recent and nationally representative data are available from the 2017–18 National Health and Nutrition Examination Survey (NHANES). To extrapolate from the observed relationships of phthalates with preterm birth and gestational age, we obtained urinary levels of MEHP, MEOHP, MEHHP, MECPP, MCOP, MiNP and monocarboxynonylphthalate (MCNP) metabolites in 16–49-year-old women (assuming similarity of these exposures to pregnant women in the USA) from the 2017–18 NHANES. We computed molar sums of MEHP, MEOHP, MEHHP, and MECPP to estimate DEHP exposure. We used measurements of MCNP to quantify DiDP exposure, and we computed molar sums of MiNP and MCOP to estimate DiNP exposure. Estimates of DnOP-attributable preterm birth were not possible because measurements of the main metabolite, mono-carboxyheptylphthalate, were not performed in that wave of NHANES. We quantified the 10th, 25th, 50th, 75th, and 90th percentiles (expressed as log_10_ of the μM concentrations using molar sums) using the appropriate subsample weights, respecting the complex survey design. Following approaches applied previously,^41^ we assumed that the lowest 10% of the US population had no exposure, and that each subsequent centile grouping (eg, 10–24th percentile) had the lowest level within the range represented by the grouping (ie, 10th percentile). We assumed no effects below the 25th percentile, and quantified exposure-response relationships from the ECHO cohorts for decrements in gestational age, using the 10th percentile in NHANES 2017–18 as a reference level.

We used natality data from the National Vital Statistics System (NVSS) of the National Center for Health Statistics^42,43^ to determine mean gestational age (38·73 weeks), total number of births (3 664 651), and number of singleton preterm births in 2018 (242 019, the exposed scenario) and then increased mean gestational age in each DEHP, DiDP, or DiNP centile by the absolute value of the attributable decrement to calculate the number of preterm births in a scenario free of DEHP, DiDP, or DiNP effects. The DEHP, DiDP, or DiNP attributable preterm birth disease burdens were the difference in preterm births between the two scenarios, assuming a normal distribution of gestational age. Using the logistic regression models of preterm birth and DEHP exposure across pregnancy in ECHO, we calculated a weighted average of increments in preterm birth across US births due to DiNP exposure as a main estimate of the burden of preterm birth in the USA in 2018 due to phthalates. Although a substantial literature suggests synergy in adverse effects across multiple phthalate exposures which occur simultaneously,^44^ as a conservative measure, we did not assume additive effects across individual phthalates.

Recognising multiple sources of potential uncertainty in attributable burden estimates, we employed several sensitivity analyses. First, we quantified attributable preterm birth applying the relationships identified in continuous models of gestational age in the ECHO cohorts and applied those relationships to exposure levels modelled from NHANES 2017–18 (using the same reference level) to quantify increments in preterm birth over and above baseline rates in 2018 from NVSS. We reprised the estimates for DiDP and DiNP, using logistic models of preterm birth and continuous models of gestational age from ECHO and exposure data from NHANES.

We calculated total costs of preterm birth attributable to in-utero exposure by adding preterm birth-associated costs of hospitalisation for medical care (direct cost) to lost lifetime economic productivity, operationalised as loss of intellectual quotient points due to preterm birth (indirect cost). The lifetime costs of preterm birth, inclusive of direct medical care, intellectual quotient loss and other indirect consequences, was estimated to be US$64 815 per case in 2016.^45^ We updated this cost to $67 836 in 2018 by applying adjustment with the Medical Care Consumer Price Index,^46^ and multiplied by attributable preterm birth to quantify societal costs associated with phthalate exposure.

To illustrate the steps applied, we used estimates of preterm birth for the 25th percentile of DEHP metabolites (expressed as a log_10_ of the μM sum). The 10th and 25th percentiles of the log_10_ of the μM sum were −1·773 and −1·597, respectively. A 0·175 log_10_ unit increase (subtracting −1·773 μM from −1·597 μM) in DEHP metabolites was multiplied by 0·15 weeks lost divided by log_10_ DEHP, producing a 0·026 week decrement in gestational age for the 25th percentile. Repeating this exercise produced decrements of 0·074 for the 50th percentile, 0·11 for the 75th percentile, and 0·14 for the 90th percentile. Applying these decrements using the NORMDIST function in Excel for the 25th percentile:

=NORM.DIST(37,38⋅73+C11,2⋅82,TRUE)-NORM.DIST(37,38⋅73,2⋅82,TRUE)

we obtain an 0·31% increase in preterm birth; repeating this exercise for the other percentiles yields 0·88% for the 50th percentile, 1·28% for the 75th percentile, and 1·67% for the 90th percentile, and a weighted average preterm birth increment of 0·65%, calculated by (0·25 × 0·31%) + (0·25 × 0·88%) +(0·15% × 1·28) + (0·10 × 1·67%). Multiplying 0·65% by 3 664 651 births yielded 24 003 attributable preterm births, and multiplying 24 003 preterm births by $67 665 yields $1 626 529 632.

### Role of the funding source

The funder had no role in study design, data collection, data analysis, data interpretation, or writing of the report.

## Results

We identified 5006 mother–child dyads from 13 cohorts in the ECHO cohort study (appendix pp 7–9) with information on up to 20 urinary phthalate metabolites. Most mothers (2810 [56%]) were aged 25–34 years at delivery (table 1). Most mothers were non-Hispanic White (2145 [43%]), and there were similar percentages of non-Hispanic Black (1210 [24%]) and Hispanic or Latino participants (1263 [25%]). Study participants were generally well educated, having received some college (907 [18%]), a bachelor’s degree (1230 [25%]), or a postgraduate degree (1106 [22%]). A low percentage reported smoking during pregnancy (285 [6%]).

Phthalate metabolite concentrations were similar to those identified in women of childbearing age in national surveys.^47^ The distributions of phthalates were similar across trimesters (figure). Generally, phthalates were not strongly correlated with one another (appendix p 86), except MECPP, MEHHP, and MEOHP, which are metabolites of DEHP. The highest concentrations measured were mono-ethyl phthalate and phthalic acid (appendix p 87). As described in other US populations,^48^ non-Hispanic Black participants had higher concentrations of multiple phthalate metabolites, except for DEHP replacements (table 2), and Hispanic or Latino mothers were found to have higher urinary concentrations of LMW metabolites and lower concentrations of the HMW phthalates, DEHP and phthalic acid. Maternal age was inversely related to all phthalate metabolites, and tobacco use and lower maternal education were associated with greater concentrations of phthalate metabolites. No differences were identified by child sex. Higher LMW metabolites and phthalic acid were identified in primiparous mothers compared with nulliparous mothers, but differences were not noted among multiparous mothers compared with nulliparous mothers.

Racial and ethnic disparities in birth outcomes were apparent (table 3), with shortened gestation and lower birthweight and length among non-Hispanic Black and Hispanic mothers compared with White mothers. Non-Hispanic Black and Hispanic mothers also had lower birthweight for gestational age z-score compared with White mothers. Maternal age was associated with higher birthweight and length, and maternal education was associated with longer gestation as well as higher birthweight and length. Parity was inversely associated with gestational age and positively associated with birthweight. Tobacco use in pregnancy was associated with reduced birth length but not gestational age or birthweight. All covariates were significantly associated with a subset or all outcomes of interest, showing the need for adjustment in full multivariate models.

Phthalate metabolites generally showed inverse associations with outcomes of interest. For continuous outcomes (table 4), point estimates for all phthalates were in a negative direction for all birth outcomes, with varying degrees of precision. Most notable, associations of phthalic acid, DiNP, DiDP, and DnOP with gestational age, birth length, and birthweight were generally of a higher magnitude than LMW, HMW, and DEHP. For example, compared with associations of DEHP with preterm birth (odds ratio 1·45 [95% CI 1·05–2·01]), the risks per log_10_ increase were higher for phthalic acid (2·71 [1·91–3·83]), DiNP (2·25 [1·67–3·00]), DiDP (1·69 [1·25–2·28]), and DnOP (2·90 [1·96–4·23]). When considering trimester specific associations, the magni tude of association was generally strongest for third trimester phthalate concentrations; we note that third trimester specific associations were generally consistent with pregnancy average-based associations, as indicated by a large degree of confidence interval overlap. Associations were somewhat attenuated or less precise, generally, for first and second trimester specific phthalate concentrations (appendix p 11). There were no notable associations of birthweight for gestational age z-scores overall or by trimester.

Associations of phthalates and binary outcomes of interest were similar (table 5). As before, pregnancy averaged concentrations of phthalic acid, DiNP, DiDP, and DnOP were most strongly associated with preterm birth and low birthweight. All phthalates of interest showed a harmful association with birthweight overall (appendix p 13). As with continuous outcomes, associations were generally somewhat stronger for third trimester phthalate concentrations; although, 95% CIs largely overlapped with pregnancy-averaged outcome associations. First and second trimester specific associations were generally less precise or of lower magnitude (appendix pp 13).

Sensitivity analyses suggested some group-specific differences in associations of phthalates to birth outcomes of interest. Most notable, the association of DnOP to preterm birth was stronger for females (RR 4·84 [95% CI 2·75–8·54]) than males (2·26 [1·30–3·91]; appendix pp 16–25). Many associations of phthalic acid, DiNP, and DnOP with birth outcomes were notably stronger among non-Hispanic White mothers and some college educated mothers (appendix pp 26–40). For example, among non-Hispanic White mothers, RRs per log_10_ increase in phthalic acid exposure were 3·91 (2·45–6·24) for preterm birth and 4·01 (2·42–6·64) for low birthweight, and were notably stronger compared with other race or ethnicity groups and full sample analyses. Among mothers with some college education, the estimated RRs associated with phthalic acid were 3·48 (2·36–5·12) for preterm birth and 3·16 (2·09–4·78) for low birthweight (appendix pp 41–50).

Differences between full sample results were not noted for parity-specific groups or for additional adjustment for tobacco exposures (appendix pp 51–70). Results considering a more granular categorisation of birth timing (preterm, early term, and late term *vs* full term) did not yield findings different from our full sample analyses (appendix p 71). Addition of pre-pregnancy BMI did not reveal changes in coefficients by more than 15% (appendix pp 72–75), nor did gestational diabetes or hypertensive disorders of pregnancy (appendix pp 76–85). Finally, we note that leave-one-out analyses showed that the magnitude of association between phthalic acid, DiNP, and DnOP with gestational age and preterm birth might have been influenced by a single cohort; however, associations were in the same direction of effect with and without inclusion of the cohort (appendix pp 88–89).

Using NHANES data on phthalate exposure and observed associations with preterm birth in ECHO, the main estimate of attributable preterm birth in 2018 is 56 595 (sensitivity analyses 24 003–120 116) with associated costs of US$3·84 billion (sensitivity analyses 1·63–8·14 billion; table 6). Sensitivity analyses using the observed relationships with continuous gestational age, the attributable cases were 24 003 and costs were $1·63 billion. Estimates derived from the observed relationships with DiDP exposure in ECHO and exposure data in NHANES ranged from 57 017 to 79 947 cases and $3·86 billion to $5·42 billion, and estimates derived from relationships with DiNP exposure in ECHO and exposure data in NHANES ranged from 76 838 to 120 116 cases and $5·21 billion to $8·14 billion.

## Discussion

We identified associations of phthalate exposure with decreased gestational age in a large and diverse sample generally representative of the USA. The patterns of association suggest DEHP replacements to be driving the increase in preterm birth. This finding is of great concern because DiNP, DiDP, and 1,2-cyclohexane dicarboxylic acid diisononyl ester are replacing DEHP in food packaging.^49^ Extrapolated nationally, the attributable burden is substantial, suggesting opportunities to decrease this early life morbidity closer to Healthy People 2020 goals. The lost economic productivity and additional medical care costs due to phthalate-induced preterm birth in 2018 alone ranges between $1·63 billion and 8·14 billion.

Strengths of the analysis include the large sample size, high quality of laboratory analyses, harmonisation approach, multiple robustness checks, and the specificity of the effect to gestational age. The ECHO consortium combines data from many cohorts representing diverse populations and exposures over time, which allowed for evaluation of the effect of replacement phthalates on birth outcomes. Most phthalates were highly detected, which minimised the need for imputing values below the analytical limit of detection. Effect modification was modest in exploratory analyses, supporting the rigor of the associations identified.

There are limitations to interpretation, which include the potential for unmeasured confounding. Because DEHP, DiDP, DiNP, and DnOP are known dietary contaminants, the lack of harmonised diet data across the cohorts is important to emphasise. The results are substantially driven by one cohort, although it should be noted that this cohort also has the highest number of cases of preterm birth in the study. The inclusion of cases from this cohort likely increased statistical power to detect effects of phthalate exposure on gestational age, reinforcing the value of ECHO in uniting cohorts with different risk profiles. Although each individual cohort by itself has limited power to observe the small effect when considered individually, the associations are consistently in the same direction. We emphasise that many other preterm birth risks (such as previous preterm birth and maternal age) are comparably small in this multifactorial condition, and also are not as readily modifiable. Pharmacokinetic studies in adults suggest that phthalates have 12–48 h half-lives,^50^ raising the potential for exposure imprecision introduced by relying on spot urine samples. We do note that weak indices of exposure could bias associations toward the null;^51–53^ although, this post-hoc justification has limits. We also acknowledge potential residual confounding by unmeasured or unknown coexposures.

Previous studies largely examined cohorts preceding the replacement of DEHP.^30,31^ We acknowledge partial overlap in the populations included in the previous systematic review^31^ and pooled analysis^30^ (two and five of the cohorts included in the present analysis, respectively). With a majority of the sample enrolled after 2010 (when DEHP replacements were increasing in use), a primary goal of the present study was to address a different question than that posed by the previous systematic review^31^ and pooled analysis:^30^ the effect of replacement phthalates. A crude comparison of the present study would suggest discrepancies, as we find fewer associations of LMW phthalates, but this would be misleading because we did not query specific LMW metabolites. Our results are largely consistent with the systematic review^31^ in identifying associations of DEHP metabolites with decreased gestational age and increased preterm birth risk. A limitation of our work compared with previous studies is our inability to distinguish spontaneous from medically-induced preterm birth.

Safer alternatives exist for phthalates, yet often the costs of these alternatives are identified as a barrier to their use. Attributable fractions of preterm birth due to these exposures have not been quantified previously, limiting the ability to weigh societal trade-offs involved in the ongoing use of these chemicals. We used methods developed by the Institute of Medicine^54^ for identifying the environmental burden and cost of illness. However, we only considered medical care, lost intellectual quotient and related loss of economic productivity, when there are other long-term consequences of preterm birth that add other social costs. Other chronic conditions due to phthalates include childhood obesity, adult obesity and diabetes, endometrio sis, male factor infertility, and cardiovascular mortality,^55^ with total costs nearly $100 billion annually. The $1·63–8·14 billion costs of preterm birth described here are also annual insofar as exposures continue at current levels. Because DEHP, DiNP, and DiDP are additives to plastics, these results also add to the disease burden and costs of plastic in the USA, which were recently estimated to be $250 billion annually.^56^

The societal benefits of replacements only exist if the replacements do not produce the same or other adverse health effects. The present analysis is truly limited as a cost of inaction to remove phthalates contributing to preterm birth. Estimates of DnOP-attributable preterm birth were not possible because measurements of the main metabolite, monocarboxyheptyl phthalate, were not performed in that wave of NHANES. We also appreciate there are many more phthalates than the 20 we studied, which is another source of underestimation of the costs of phthalate exposure. Our findings also support individual behavioural interventions to reduce exposure. These include choosing personal care products labelled to be free of phthalates,^57^ and replacement of packaged foods with fresh foods.^58^

In a large, diverse US sample, DEHP, DiDP, DiNP, and phthalic acid exposures were associated with decreases in gestational age and increased preterm birth risk, suggesting opportunities for prevention. The contribution is substantial, with 56 595 preventable preterm birth cases and associated costs of $3·84 billion. The adverse consequences of chemically similar phthalates to DEHP suggest a need to regulate chemicals with similar properties as a class.

## Supplementary Material

1

## Figures and Tables

**Figure:**
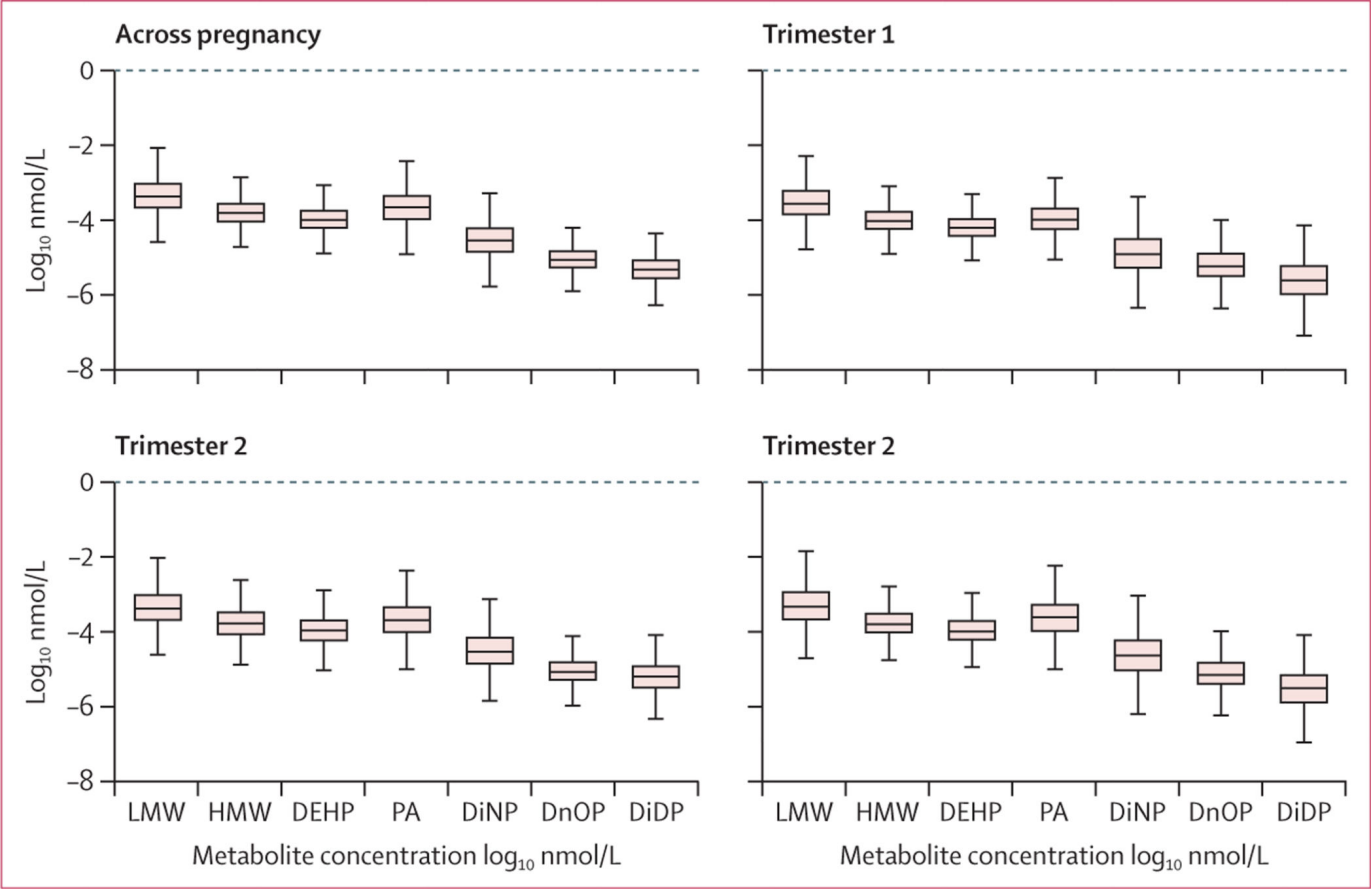
Distribution of phthalate metabolites in women during pregnancy Thick bands represent the median, the boxes represent the IQR, and the whiskars represent the range. DEHP=di-12-ethylhexyl phthalate. DiDP=diisodecyl phthalate. DiNP=diisononyl phthalate. DnOP=di-n-octyl phthalate. HMW=high molecular weight. LMW=low molecular weight. PA=phthalic acid.

**Table 1:** Description of the study population

	Study population

**Maternal variables**	
Age category	
<25 years	1376/5006 (28%)
25–34 years	2810/5006 (56%)
≥35 years	820/5006 (16%)
Race or ethnicity	
Non-Hispanic White	2145/5006 (43%)
Non-Hispanic Black	1210/5006 (24%)
Hispanic or Latino	1263/5006 (25%)
Other or unknown	388/5006 (8%)
Education	
Less than high school	554/5006 (11%)
High school degree, GED, or equivalent	1178/5006 (24%)
Some college, no degree; associate’s degree; or trade school	907/5006 (18%)
Bachelor’s degree	1230/5006 (25%)
Postgraduate degree	1106/5006 (22%)
Data missing	31/5006 (1%)
Parity	
0	1907/5006 (38%)
1	1729/5006 (35%)
2	666/5006 (13%)
3	230/5006 (5%)
>4	145/5006 (3%)
Data missing	329/5006 (7%)
Tobacco use during pregnancy	
Yes	285/5006 (6%)
No	3720/5006 (74%)
Data missing	1001/5006 (20%)
**Child variables**	
Child sex	
Male	2524/5006 (50%)
Female	2477/5006 (50%)
Data missing	5/5006 (<1%)
**Exposure distributions**	
Median LMW phthalate metabolites, nmol/L	
Across pregnancy	423 (713·4); 4730
Trimester 1	283 (472·3); 1248
Trimester 2	406 (722·2); 3424
Trimester 3	467 (936·7); 3390
Median HMW phthalate metabolites, nmol/L	
Across pregnancy	155 (180·7); 4798
Trimester 1	97 (111·3); 1428
Trimester 2	161 (233·2); 3362
Trimester 3	160 (206·9); 3124
Median DEHP metabolites, nmol/L	
Across pregnancy	101 (115·5); 4806
Trimester 1	65 (70·9); 1457
Trimester 2	105 (138·7); 3369
Trimester 3	102 (133·6); 3137
Median phthalic acid, nmol/L	
Across pregnancy	220 (335·2); 2891
Trimester 1	107 (150·1); 1154
Trimester 2	198 (340·5); 2331
Trimester 3	247 (418·5); 2408
Median DiNP metabolites, nmol/L	
Across pregnancy	28 (46); 3129
Trimester 1	13 (26·5); 1078
Trimester 2	29 (54·2); 2562
Trimester 3	23 (49·7); 1666
Median DnOP metabolites, nmol/L	
Across pregnancy	9 (9·2); 2320
Trimester 1	6 (9·9); 1061
Trimester 2	8 (10); 1925
Trimester 3	7 (10·9); 1481
Median DiDP metabolites, nmol/L	
Across pregnancy	5 (5·7); 4109
Trimester 1	3 (5·1); 1166
Trimester 2	6 (8·5); 3268
Trimester 3	3 (5·7); 2855
**Outcome distributions**	
Mean gestational age at birth, weeks	38·8 (1·82); 5006
Number of preterm births	394/5006 (8%)
Mean length at birth, cm	50·4 (3·02); 4238
Mean birthweight, g	3312 (544); 5006
Number of small for gestational age	342/4992 (7%)
Number of large for gestational age	759/4992 (15%)
Mean birthweight for gestational age, z-score	0 (1·08); 4447
Number of low birthweight, all	307/5006 (6%)
Number of low birthweight, preterm	179/394 (45%)
Number of low birthweight, term	128/4612 (3%)

Data are n/N (%), median (IQR); N, or mean (SD); N. DEHP=di-2-ethylhexyl phthalate. DiDP=diisodecyl phthalate. DiNP=diisononyl phthalate. DnOP=di-noctyl phthalate. GED=general educational development. HMW=high molecular weight. LMW=low molecular weight.

**Table 2:** Associations of exposures with covariates

	LMW phthalate	HMW phthalate	DEHP	Phthalic acid	DiNP	DnOP	DiDP

Maternal age	−0·019 (−0·021 to −0·017)*	−0·013 (−0·015 to −0·011)*	−0·010 (−0·012 to −0·009)*	−0·018 (−0·021 to −0·015)*	0·003 (−0·001 to 0·006)	0·003 (0·000 to 0·005)*	0·004 (0·002 to 0·006)*
Race							
Non-Hispanic Black *vs* non-Hispanic White	0·471 (0·439 to 0·503)*	0·216 (0·19 to 0·242)*	0·195 (0·168 to 0·222)*	0·355 (0·318 to 0·391)*	−0·171 (−0·217 to −0·125)*	−0·076 (−0·112 to −0·040)*	−0·080 (−0·114 to −0·045)*
Hispanic or Latino *vs* non-Hispanic White	0·180 (0·149 to 0·212)*	0·034 (0·008 to 0·059)*	0·082 (0·055 to 0·108)*	−0·111 (−0·156 to −0·066)*	−0·208 (−0·259 to −0·156)*	−0·119 (−0·159 to −0·078)*	−0·073 (−0·107 to −0·039)*
Other or unknown *vs* non-Hispanic White	0·058 (0·008 to 0·107)*	0·003 (−0·037 to 0·043)	0·024 (−0·018 to 0·066)	−0·005 (−0·061 to 0·051)	−0·079 (−0·144 to −0·015)*	−0·061 (−0·112 to −0·0100)*	−0·040 (−0·090 to 0·011)
Higher education *vs* high school degree, GED, or equivalent	−0·285 (−0·313 to −0·258)*	−0·186 (−0·208 to −0·165)*	−0·156 (−0·178 to −0·134)*	−0·219 (−0·252 to −0·186)*	0·169 (0·129 to 0·208)*	0·053 (0·022 to 0·084)*	0·100 (0·071 to 0·128)*
Parity							
1 *vs* 0	−0·073 (−0·105 to −0·04)*	−0·019 (−0·044 to 0·006)	−0·015 (−0·041 to 0·011)	−0·122 (−0·162 to −0·083)*	−0·059 (−0·103 to −0·015)*	−0·029 (−0·065 to 0·006)	−0·054 (−0·085 to −0·023)*
>2 *vs* 0	−0·003 (−0·041 to 0·034)	0·080 (0·051 to 0·109)*	0·044 (0·014 to 0·074)*	0·043 (−0·004 to 0·09)	0·012 (−0·042 to 0·066)	−0·025 (−0·068 to 0·019)	−0·053 (−0·090 to −0·016)*
Tobacco use *vs* no tobacco use	0·049 (−0·009 to 0·107)	0·077 (0·031 to 0·124)*	0·036 (−0·012 to 0·083)	0·113 (0·047 to 0·178)*	0·078 (−0·002 to 0·159)	0·045 (−0·015 to 0·104)	0·011 (−0·042 to 0·064)
Child sex: female *vs* male	−0·007 (−0·035 to 0·020)	0·013 (−0·008 to 0·034)	0·007 (−0·014 to 0·029)	0·011 (−0·022 to 0·045)	−0·001 (−0·038 to 0·036)	0·000 (−0·029 to 0·029)	−0·004 (−0·031 to 0·023)

Data are beta (95% CI). DEHP=di-2-ethylhexyl phthalate. DiDP=diisodecyl phthalate. DiNP=diisononyl phthalate. DnOP=di-n-octyl phthalate. GED=general educational development. HMW=high molecular weight. LMW=low molecular weight.

* Indicates significance criteria were met.

**Table 3:** Associations of outcomes with covariates

	Gestational age	Preterm birth	Birth length	Birthweight	Small for gestational age	Large for gestational age	Birthweight for gestational age	Low birthweight

Maternal age	−0·005 (−0·013 to 0·004)	1·01 (0·99 to 1·02)	0·040 (0·025 to 0·056)*	9·549 (7·027 to 12·070)*	0·97 (0·95 to 0·98)*	1·05 (1·03 to 1·06)*	0·020 (0·015 to 0·026)*	1·01 (0·99 to 1·03)
Race								
Non-Hispanic Black *vs* non-Hispanic White	−0·375 (−0·503 to −0·247)*	1·63 (1·27 to 2·1)*	−1·075 (−1·301 to −0·849)*	−266·808 (−304·374 to −229·242)*	2·06 (1·66 to 2·55)*	0·44 (0·33 to 0·57)*	−0·522 (−0·601 to −0·444)*	2·07 (1·56 to 2·73)*
Hispanic or Latino *vs* non-Hispanic White	−0·195 (−0·322 to −0·069)*	1·2 (0·92 to 1·57)	−0·596 (−0·822 to −0·371)*	−126·134 (−163·192 to −89·075)*	1·38 (1·1 to 1·73)*	0·73 (0·58 to 0·92)*	−0·236 (−0·314 to −0·158)*	1·18 (0·86 to 1·62)
Other or unknown *vs* non-Hispanic White	−0·246 (−0·442 to −0·05)*	1·43 (0·97 to 2·11)	−0·625 (−0·984 to −0·265)*	−141·928 (−199·57 to −84·286)*	1·77 (1·29 to 2·44)*	0·67 (0·46 to 0·98)*	−0·297 (−0·421 to −0·173)*	1·53 (0·99 to 2·37)
Higher education *vs* high school degree, GED, or equivalent	0·106 (0 to 0·212)	0·77 (0·62 to 0·95)*	0·565 (0·375 to 0·755)*	120·901 (89·425 to 152·376)*	0·69 (0·58 to 0·83)*	1·42 (1·15 to 1·74)*	0·279 (0·213 to 0·344)*	0·76 (0·6 to 0·96)*
Parity								
1 *vs* 0	−0·150 (−0·267 to −0·033)*	0·94 (0·73 to 1·2)	0·158 (−0·052 to 0·367)	110·166 (75·118 to 145·214)*	0·69 (0·57 to 0·85)*	1·88 (1·49 to 2·37)*	0·095 (0·023 to 0·167)*	0·81 (0·61 to 1·06)
>2 *vs* 0	−0·166 (−0·302 to −0·030)*	1·17 (0·89 to 1·54)	0·024 (−0·224 to 0·273)	104·797 (64·125 to 145·468)*	0·57 (0·44 to 0·73)*	1·93 (1·49 to 2·49)*	0·031 (−0·052 to 0·115)	0·81 (0·59 to 1·12)
Tobacco use *vs* no tobacco use	−0·045 (−0·267 to 0·177)	0·93 (0·59 to 1·46)	−0·621 (−1·038 to −0·205)*	−62·469 (−127·463 to 2·524)	1·32 (0·93 to 1·88)	0·94 (0·62 to 1·43)	−0·174 (−0·305 to −0·042)*	1·22 (0·77 to 1·94)
Child sex: female *vs* male	0·047 (−0·054 to 0·148)	0·84 (0·68 to 1·03)	−0·764 (−0·944 to −0·583)*	−122·559 (−152·508 to −92·610)*	1·03 (0·86 to 1·22)	1·05 (0·87 to 1·26)	−0·010 (−0·074 to 0·054)	1·30 (1·03 to 1·64)*


Data are beta (95% CI). GED=general educational development.

* Indicates significance criteria were met.

**Table 4:** Adjusted associations of phthalate exposures with birth outcomes (continuously measured)

	Gestational age		Birth length		Birthweight		Birthweight for gestational age	
	Across pregnancy	Trimester 3	Across pregnancy	Trimester 3	Across pregnancy	Trimester 3	Across pregnancy	Trimester 3

LMW	−0·095 (−0·224 to 0·034); 4389	−0·126 (−0·249 to −0·002)*; 3097	−0·279 (−0·511 to −0·047)*; 3721	−0·273 (−0·496 to −0·051)*; 2657	−26·520 (−64·069 to 11·03); 4389	−41·921 (−79·536 to −4·307)*; 3097	−0·070 (−0·148 to 0·007); 4160	−0·068 (−0·148 to 0·012); 2881
HMW	−0·230 (−0·400 to −0·061)*; 4446	−0·248 (−0·426 to −0·070)*; 2831	−0·238 (−0·539 to 0·062); 3851	−0·334 (−0·653 to −0·016)*; 2417	−36·316 (−86·273 to 13·641); 4446	−37·415 (−90·984 to 16·153); 2831	0·018 (−0·083 to 0·119); 4217	0·007 (−0·106 to 0·119); 2615
DEHP	−0·253 (−0·414 to −0·091)*; 4454	−0·287 (−0·452 to −0·121)*; 2844	−0·234 (−0·520 to 0·052); 3859	−0·314 (−0·611 to −0·017)*; 2430	−45·720 (−93·318 to 1·879); 4454	−69·053 (−119·544 to −18·563)*; 2844	0·010 (−0·087 to 0·106); 4225	−0·025 (−0·130 to 0·079); 2628
PA	−0·671 (−0·868 to −0·475)*; 2607	−0·586 (−0·737 to −0·435)*; 2143	−0·892 (−1·284 to −0·501)*; 2054	−0·762 (−1·063 to −0·461)*; 1772	−162·867 (−220·254 to −105·479)*; 2607	−133·082 (−179·494 to −86·670)*; 2143	−0·058 (−0·170 to 0·053); 2388	−0·024 (−0·118 to 0·069); 1934
DiNP	−0·485 (−0·637 to −0·334)*; 2845	−0·533 (−0·716 to −0·349)*; 1400	−0·562 (−0·830 to −0·295)*; 2456	−0·650 (−0·998 to −0·302)*; 1071	−93·494 (−137·162 to −49·827)*; 2845	−105·904 (−153·879 to −57·929)*; 1400	0·024 (−0·062 to 0·110); 2624	−0·024 (−0·127 to 0·080); 1190
DnOP	−0·632 (−0·882 to −0·382)*; 2037	−0·530 (−0·764 to −0·295)*; 1217	−0·594 (−1·046 to −0·141)*; 1653	−0·659 (−1·109 to −0·209)*; 887	−144·028 (−214·002 to −74·055)*; 2037	−143·537 (−211·303 to −75·772)*; 1217	−0·039 (−0·173 to 0·094); 1818	−0·090 (−0·227 to 0·048); 1008
DiDP	−0·261 (−0·407 to −0·114)*; 3775	−0·148 (−0·286 to −0·009)*; 2568	−0·234 (−0·496 to 0·029); 3226	−0·159 (−0·407 to 0·088); 2198	−51·721 (−92·991 to −10·452)*; 3775	−37·630 (−79·739 to 4·479); 2568	−0·014 (−0·102 to 0·075); 3553	−0·030 (−0·116 to 0·056); 2358

Data are beta (95% CI); N. Models adjusted for maternal race or ethnicity, parity, and education, and child sex; cohort as random effect. DEHP=di-2-ethylhexyl phthalate. DiDP=diisodecyl phthalate. DiNP=diisononyl phthalate. DnOP=di-n-octyl phthalate. HMW=high molecular weight. LMW=low molecular weight. PA=phthalic acid.

* Indicates significance criteria were met.

**Table 5:** Adjusted associations of phthalate exposures with categorical outcomes

	Preterm birth		Small for gestational age		Large for gestational age		Low birthweight	
	Across pregnancy	Trimester 3	Across pregnancy	Trimester 3	Across pregnancy	Trimester 3	Across pregnancy	Trimester 3

LMW	1·02 (0·77 to 1·33); 4389	1·10 (0·84 to 1·45); 3097	1·01 (0·81 to 1·27); 4382	0·90 (0·71 to 1·13); 3091	0·96 (0·75 to 1·23); 4382	1·02 (0·80 to 1·30); 3091	1·26 (0·94 to 1·69); 4389	1·16 (0·87 to 1·57); 3097
HMW	1·52 (1·08 to 2·15)*; 4446	1·69 (1·18 to 2·44)*; 2831	1·05 (0·78 to 1·43); 4438	1·03 (0·74 to 1·43); 2825	0·95 (0·69 to 1·30); 4438	0·91 (0·64 to 1·29); 2825	1·52 (1·03 to 2·24)*; 4446	1·34 (0·89 to 2·01); 2831
DEHP	1·45 (1·05 to 2·01)*; 4454	1·72 (1·23 to 2·40)*; 2844	1·07 (0·80 to 1·43); 4446	1·01 (0·75 to 1·37); 2838	0·92 (0·68 to 1·24); 4446	0·82 (0·59 to 1·15); 2838	1·55 (1·08 to 2·23)*; 4454	1·55 (1·07 to 2·25)*; 2844
PA	2·71 (1·91 to 3·83)*; 2607	2·40 (1·74 to 3·32)*; 2143	1·06 (0·75 to 1·51); 2607	1·02 (0·76 to 1·36); 2143	1·16 (0·86 to 1·57); 2607	1·01 (0·78 to 1·31); 2143	2·78 (1·91 to 4·04)*; 2607	2·07 (1·47 to 2·92)*; 2143
DiNP	2·25 (1·69 to3·00)*; 2845	2·35 (1·69 to 3·26)*; 1400	1·03 (0·79 to 1·34); 2845	1·09 (0·80 to 1·47); 1400	1·08 (0·83 to 1·42); 2845	0·89 (0·67 to 1·19); 1400	2·13 (1·55 to 2·93)*; 2845	1·99 (1·4 to 2·82)*; 1400
DnOP	2·90 (1·96 to 4·29)*; 2037	2·48 (1·71 to 3·60)*; 1217	1·05 (0·71 to 1·56); 2037	1·26 (0·88 to 1·81); 1217	0·97 (0·64 to 1·47); 2037	0·88 (0·60 to 1·28); 1217	2·05 (1·36 to 3·11)*; 2037	1·99 (1·34 to 2·94)*; 1217
DiDP	1·69 (1·25 to 2·28)*; 3775	1·44 (1·06 to 1·96)*; 2568	1·04 (0·80 to 1·35); 3775	1·01 (0·80 to 1·28); 2568	1·20 (0·91 to 1·57); 3775	1·12 (0·86 to 1·48); 2568	1·65 (1·19 to 2·29)*; 3775	1·3 (0·94 to 1·80); 2568

Data are OR (95% CI); N. Models adjusted for maternal race or ethnicity, parity, and education, and child sex; cohort as random effect. DEHP=di-2-ethylhexyl phthalate. DiDP=diisodecyl phthalate. DiNP=diisononyl phthalate. DnOP=di-n-octyl phthalate. HMW=high molecular weight. LMW=low molecular weight. PA=phthalic acid.

* Indicates significance criteria were met.

**Table 6:** Estimates of phthalate-attributable preterm birth in the USA*

	Exposure, log_10_ of μM DEHP	Decrement in gestational age, continuous model	Increment in preterm birth, continuous model†	Risk ratio for preterm birth, logistic model	Increment in preterm birth, logistic model†

**DEHP-derived estimates**					
0–9th percentile	No exposure assumed	NA	··	··	··
10–24th percentile	−1·773	0 (ref)	NA	0 (ref)	NA
25–49th percentile	−1·597	0·026 weeks	0·31%	1·07	0·73%
50–74th percentile	−1·278	0·074 weeks	0·88%	1·22	2·18%
75–90th percentile	−1·055	0·107 weeks	1·28%	1·24	2·42%
>90th percentile	−0·834	0·141 weeks	1·67%	1·45	4·55%
Weighted mean	··	··	0·65%	··	1·54%
Number of attributable preterm births	··	··	24 003	··	56 595
Attributable cost of preterm birth‡	··	··	$1 626 529 632	··	$3 835 175 861
**DiNP-derived estimates**					
0–9th percentile	No exposure assumed	NA	··	··	··
10–24th percentile	−2·239	0 (ref)	NA	0 (ref)	NA
25–49th percentile	−2·044	−0·090 weeks	1·06%	1·15	1·55%
50–74th percentile	−1·768	−0·217 weeks	2·60%	1·42	4·17%
75–90th percentile	−1·533	−0·325 weeks	3·93%	1·46	4·58%
>90th percentile	−1·195	−0·480 weeks	5·90%	2·16	11·61%
Weighted mean	··	··	2·10%	··	3·28%
Number of attributable preterm births	··	··	76 838	··	120 116
Attributable cost of preterm birth‡	··	··	$5 206 900 312	··	$8 139 653 757
**DiDP-derived estimates**					
0–9th percentile	No exposure assumed	NA	··	··	··
10–24th percentile	−3·054	0 (ref)	NA	0 (ref)	NA
25–49th percentile	−2·753	−0·084 weeks	1·00%	1·14	1·44%
50–74th percentile	−2·452	−0·168 weeks	2·01%	1·31	3·08%
75–90th percentile	−2·209	−0·237 weeks	2·84%	1·27	2·74%
>90th percentile	−1·941	−0·311 weeks	3·77%	1·64	6·42%
Weighted mean	··	··	1·56%	··	2·18%
Number of attributable preterm births	··	··	57 017	··	79 947
Attributable cost of preterm birth‡	··	··	$3 863 771 793	··	$5 417 630 491

Costs are in US$. DEHP=di-2-ethylhexyl phthalate. DiDP=diisodecyl phthalate. DiNP=diisononyl phthalate. NA=not applicable.

* Estimates of di-n-octyl phthalate-attributable preterm birth were not possible because measurements of the main metabolite, monocarboxyheptylphthalate, were not performed in that wave of the National Health and Nutrition Examination Survey.

† Mean gestational age 38·73 weeks (SD 2·82).^43,44^

‡ Total cost per preterm birth in 2018 was $67 665.

## Data Availability

Statistical code to reproduce results are maintained by and available from the ECHO Data Analysis Center. The ECHO Program governs access to primary data through public-use and restricted-use datasets. Policies and procedures for access are available at https://echochildren.org./.

## Research in context

### Evidence before this study

Before conducting the analysis, we reviewed the existing literature using PubMed with search terms “phthalates” and “birth weight”, “gestational age”, “premature birth”, “prematurity”, and “preterm” published from database inception up until Jan 1, 2023. No language restrictions were applied to this search. This search yielded multiple cohort studies, systematic reviews, and meta-analyses, which have identified largely consistent relationships of prenatal phthalate exposures with decreased gestational length and preterm birth. Some limitations in interpretation include imprecision due to the use of only one or two spot urine samples in pregnancy to evaluate exposure, the short half-life of most phthalates, and the relatively modest number of studies that have examined effects in Hispanic, Latino, or other minority populations. Additionally, the potential effects of phthalate-based replacements of di-2-ethylhexyl phthalate (DEHP) are less well characterised. The attributable fraction of adverse birth outcomes due to these chemical exposures has not been quantified, limiting the ability to weigh the societal trade-offs involved in the ongoing use of these chemicals of concern.

### Added value of this study

We leveraged a large, diverse US cohort to study associations of phthalate metabolites, including replacements of DEHP, with fetal growth and gestational length, and estimated attributable adverse birth outcomes and associated costs.

### Implications of all the available evidence

Our results suggest substantial opportunities for prevention of preterm birth. The findings suggest adverse consequences of substitution of DEHP with chemically similar phthalates and the need to regulate chemicals with similar properties as a class.
