# Supplementary material for: Prenatal phthalate exposure and adverse birth outcomes in the USA: a prospective analysis of births and estimates of attributable burden and costs
Source: Lancet Planet Health. Author manuscript; Available in PMC 2024 Oct 1. (PMC11444077; doi:10.1016/S2542-5196(23)00270-X)
Supplement: 1 [file NIHMS1965320-supplement-1.pdf]

THE LANCET
Planetary Health

Supplementary appendix

This appendix formed part of the original submission and has been peer reviewed. We post it as supplied by the authors.

Supplement to: Trasande L, Nelson ME, Alshawabkeh A, et al. Prenatal phthalate exposure and adverse birth outcomes in the USA: a prospective analysis of births and estimates of attributable burden and costs. *Lancet Planet Health* 2024; **8:** e74–85.

Prenatal Phthalate Exposure and Adverse Birth Outcomes: a Prospective Analysis of U.S. Births and Estimates of Attributable Burden and Costs

Leonardo Trasande, MD, MPP[1-3+‡*]

Morgan E. Nelson, MS[4]

Akram Alshawabkeh, PhD[5+]

Emily S. Barrett, PhD[6]

Jessie P. Buckley, PhD[7,18]

Dana Dabelea, MD, PhD[8+]

Anne L. Dunlop, MD, MPH[9]

Julie B. Herbstman, PhD[10+]

John D. Meeker, PhD[11+]

Mrudula Naidu, MPH[1]

Craig Newschaffer, PhD[12+]

Amy M. Padula, PhD[13]

Megan E. Romano, PhD[14]

Douglas M. Ruden, PhD[15+]

Sheela Sathyanarayana, MD, MPH[16,17+]

Susan L. Schantz, PhD[18+]

Anne P. Starling, PhD[8, 19]

Ghassan B. Hamra, PhD[20]

[1] Department of Pediatrics, Division of Environmental Pediatrics, NYU Grossman School of Medicine, New York, NY, USA

[2] Department of Population Health, NYU Grossman School of Medicine, New York, NY, USA

[3] NYU Wagner School of Public Service, New York, NY, USA

[4]RTI International, Research Triangle Park, NC, USA

[5]Northeastern University, Boston, MA, USA

[6]Department of Epidemiology, Rutgers University School of Public Health, Piscataway, NJ, USA

[7]Department of Environmental Health and Engineering, Johns Hopkins Bloomberg School of Public Health, Baltimore, MD, USA

[8]Lifecourse Epidemiology Adiposity and Diabetes (LEAD) Center, University of Colorado Anschutz Medical Campus, Aurora, CO, USA

[9]Department of Gynecology and Obstetrics, Emory University School of Medicine, Atlanta, GA, USA

[10]Department of Environmental Health Sciences, Columbia University Mailman School of Public Health, New York, NY, USA

[11]Department of Environmental Health Sciences, University of Michigan School of Public Health, Ann Arbor, MI, USA

[12]College of Human Health and Development, Penn State University, Hershey, PA, USA

[13]Department of Obstetrics, Gynecology and Reproductive Sciences, University of California, San Francisco, San Francisco, CA, USA

[14]Department of Epidemiology, Geisel School of Medicine at Dartmouth, Lebanon, NH, USA

[15]Department of Obstetrics and Gynecology, Wayne State University, Detroit, MI 48201

[16]Seattle Children's Research Institute, Seattle, WA, USA

[17]Department of Pediatrics, University of Washington, Seattle, WA, USA

[18]Beckman Institute for Advanced Science and Technology, University of Illinois at Urbana-Champaign, Urbana, IL

[19]Department of Epidemiology, Gillings School of Global Public Health, University of North Carolina at Chapel Hill, Chapel Hill, NC, USA

[20]Department of Epidemiology, Johns Hopkins Bloomberg School of Public Health, Baltimore, MD, USA

on behalf of program collaborators for Environmental influences on Child Health Outcomes*

+indicates full professor status

*See Acknowledgments for full listing of collaborators

‡ Corresponding Author: Leonardo Trasande, MD, MPP, Department of Pediatrics, New York University School of Medicine, 227 East 30th Street Rm 807, New York, NY 10016, leonardo.trasande@nyulangone.org, phone 646-501-2520, fax 646-754-9688

ECHO Collaborators Acknowledgment:

The authors wish to thank our ECHO colleagues; the medical, nursing, and program staff; and the children and families participating in the ECHO cohorts. We also acknowledge the contribution of the following ECHO program collaborators:

ECHO Components—Coordinating Center: Duke Clinical Research Institute, Durham, North Carolina: Smith PB, Newby KL; Data Analysis Center: Johns Hopkins University Bloomberg School of Public Health, Baltimore, Maryland: Jacobson LP; Research Triangle Institute, Durham, North Carolina: Catellier DJ; Person-Reported Outcomes Core: Northwestern University, Evanston, Illinois: Gershon R, Cella D.

ECHO Awardees and Cohorts—University of Georgia, Athens, GA: Cordero J; University of Tennessee Health Science Center, Memphis, TN: Tylavsky F, Mason A, Zhao Q; University of California, San Francisco, San Francisco, CA: Bush N, LeWinn KZ; AJ Drexel Autism Institute, Philadelphia, PA: Lyall K; John Hopkins Bloomberg School of Public Health, Baltimore, MD: Volk H; University of California Davis Health, MIND Institute, Davis, CA: Schmidt R; Michigan State University, East Lansing, MI: Kerver JM; Henry Ford Health, Detroit, MI: Barone C; Michigan Department of Health and Human Services, Lansing, MI: Fussman C; Michigan State University, East Lansing, MI: Paneth N; University of Michigan, Ann Arbor, MI: Elliott M; University of Minnesota, Minneapolis, MN: Nguyen R; Icahn School of Medicine at Mount Sinai, New York, NY: Swan S, Columbia University, NY: Herbstman J.

NIH Funding Acknowledgment and Disclaimer:

The content is solely the responsibility of the authors and does not necessarily represent the official views of the National Institutes of Health.

Research reported in this publication was supported by the National Institute of Environmental Health Sciences under award P2CES033423 and the Environmental influences on Child Health Outcomes (ECHO) program, Office of the Director, National Institutes of Health, under Award Numbers U2COD023375 (Coordinating Center), U24OD023382 (Data Analysis Center), U24OD023319 with co-funding from the Office of Behavioral and Social Science Research (PRO Core), UH3OD023285 (Kerver), UH3OD023305 (Trasande), UH3OD023251 (Alshawabkeh), UH3OD023248 (Dabelea), UH3OD023318 (Dunlop), UH3OD023271 (Karr), UH3OD023342 (Lyall), UH3OD023272 (Schantz), UH3OD023290 (Herbstman).

Table of Contents

Supplement Table 0: Cohorts Included in Present Study.
Supplement Table 1: Equations for calculating molar sums
Supplement Table 2a: Adjusted Associations of Phthalate Exposures with Birth Outcomes (Continuously Measured)
Supplement Table 2b: Adjusted Associations of Phthalate Exposures with Categorical Birth Outcomes
Supplement Table 3a1: Sex-Stratified Adjusted Associations of Phthalate Exposures with Birth Outcomes (Continuously Measured), Male Children Only
Supplement Table 3b1: Sex-Stratified Adjusted Associations of Phthalate Exposures with Categorical Birth Outcomes, Male Children Only
Supplement Table 3a2: Sex-Stratified Adjusted Associations of Phthalate Exposures with Birth Outcomes (Continuously Measured), Female Children Only
Supplement Table 3b2: Sex-Stratified Adjusted Associations of Phthalate Exposures with Categorical Birth Outcomes, Female Children Only
Supplement Table 4a1: Race/Ethnicity-Stratified Adjusted Associations of Phthalate Exposures with Birth Outcomes (Continuously Measured), Non-Hispanic White Only
Supplement Table 4b1: Race/Ethnicity -Stratified Adjusted Associations of Phthalate Exposures with Categorical Birth Outcomes, Non-Hispanic White Only
Supplement Table 4a2: Race/Ethnicity-Stratified Adjusted Associations of Phthalate Exposures with Birth Outcomes (Continuously Measured), Non-Hispanic Black Only
Supplement Table 4b2: Race/Ethnicity -Stratified Adjusted Associations of Phthalate Exposures with Categorical Birth Outcomes, Non-Hispanic Black Only
Supplement Table 4a3: Race/Ethnicity-Stratified Adjusted Associations of Phthalate Exposures with Birth Outcomes (Continuously Measured), Hispanic Only
Supplement Table 4b3: Race/Ethnicity -Stratified Adjusted Associations of Phthalate Exposures with Categorical Birth Outcomes, Hispanic Only
Supplement Table 5a1: Education-Stratified Adjusted Associations of Phthalate Exposures with Birth Outcomes (Continuously Measured), High School Education/GED or less Only
Supplement Table 5b1: Education-Stratified Adjusted Associations of Phthalate Exposures with Categorical Birth Outcomes, High School Education/GED or less Only
Supplement Table 5a2: Education-Stratified Adjusted Associations of Phthalate Exposures with Birth Outcomes (Continuously Measured), Some College or More Only
Supplement Table 5b2: Education-Stratified Adjusted Associations of Phthalate Exposures with Categorical Birth Outcomes, Some College or More Only
Supplement Table 6a1: Parity-Stratified Adjusted Associations of Phthalate Exposures with Birth Outcomes (Continuously Measured), Parity=0 Only
Supplement Table 6b1: Parity-Stratified Adjusted Associations of Phthalate Exposures with Categorical Birth Outcomes, Parity=0 Only
Supplement Table 6a2: Parity-Stratified Adjusted Associations of Phthalate Exposures with Birth Outcomes

(Continuously Measured), Parity=1 Only
Supplement Table 6b2: Parity-Stratified Adjusted Associations of Phthalate Exposures with Categorical Birth Outcomes, Parity=1 Only
Supplement Table 6a3: Parity-Stratified Adjusted Associations of Phthalate Exposures with Birth Outcomes (Continuously Measured), Parity=2+ Only
Supplement Table 6b3: Parity-Stratified Adjusted Associations of Phthalate Exposures with Categorical Birth Outcomes, Parity=2+ Only
Supplement Table 7a: Sensitivity Analysis for Adjusted Associations of Phthalate Exposures with Birth Outcomes (Continuously Measured), Adding Tobacco as a Covariate
Supplement Table 7b: Sensitivity Analysis for Adjusted Associations of Phthalate Exposures with Categorical Birth Outcomes, Adding Tobacco as a Covariate
Supplement Table 8: Nominal Logistic Analysis for Adjusted Associations of Phthalate Exposures with 4 Category Gestational Age

Supplement Table 9: Sensitivity Analysis for Adjusted Associations of Phthalate Exposures with Birth Outcomes, Adding Pre-Pregnancy BMI as a covariate
Supplement Table 10a1: Sensitivity Analysis for Adjusted Associations of Phthalate Exposures with Birth Outcomes (Continuously Measured), Adding Gestational Diabetes (GDM) as a Covariate
Supplement Table 10b1: Sensitivity Analysis for Adjusted Associations of Phthalate Exposures with Categorical Birth Outcomes, Adding Gestational Diabetes (GDM) as a Covariate
Supplement Table 10a2: Sensitivity Analysis for Adjusted Associations of Phthalate Exposures with Birth Outcomes (Continuously Measured), Adding Gestational Hypertension (GHTN) as a Covariate
Supplement Table 10b2: Sensitivity Analysis for Adjusted Associations of Phthalate Exposures with Categorical Birth Outcomes, Adding Gestational Hypertension (GHTN) as a Covariate
Supplement Figure 1: Depicting Correlations Among Measured Phthalates
Supplement Figure 2: Distribution of Individual Analytes Across Pregnancy
Figure 3a: Leave-One-Out Analysis: Birth Gestational Age
Figure 3b: Leave-One-Out Analysis: Preterm Birth

Supplement Table 0: Cohorts Included in Present Study.

Cohort ID	Cohort Name	N	Location	Type of Cohort	Target Population	Years of birth	Trimester of Phthalates Sample	Phthalates Lab
10101	ECHO in Puerto Rico (PROTECT)	356	Puerto Rico	Clinic/hospital-based	General population<br><br>Healthy women with singleton pregnancies in northern Puerto Rico	2012-2022	1st, 2nd, 3rd	CDC
10601	Healthy Start	446	CO	Clinic/hospital-based; Community-based	General population<br><br>Healthy pregnant women without prior adverse birth outcomes	2010-2014	2nd, 3rd	CDC
10901	Atlanta ECHO Cohort of Emory University	88	Atlanta, GA	Clinic/hospital-based	General population<br><br>Healthy, African-American, pregnant women with singletons	2014-2020	1st, 2nd, 3rd	HHEAR - Emory
11901	Conditions Affecting Neurocognitive Development and Learning in Early Childhood (CANDLE)	1159	Shelby County, TN	Clinic/hospital-based; Demographically representative sample of Shelby County, TN	General population<br><br>Healthy pregnant women with singletons	2007-2011	2nd, 3rd	HHEAR - Wadsworth
11903	The Global Alliance to Prevent Prematurity and Stillbirth (GAPPS)	379	Yakima, WA Seattle, WA	Clinic/hospital-based	General population<br><br>Pregnant women with singletons	2011-2017	1st, 2nd, 3rd	HHEAR - Wadsworth

12513	University of California - Markers of Autism Risk in Babies (MARBLES)	90	Sacramento, CA	Clinic/hospital-based; Community-based	At increased likelihood of ASD due to family history<br><br>Pregnant women with at least one older child with ASD	2006-2017	2nd, 3rd	CDC
12515	Early Autism Risk Longitudinal investigation (EARLI)	90	Oakland, CA Sacramento, CA Baltimore MD	Community-based	At increased likelihood of ASD due to family history<br><br>Pregnant women with at least one older child with ASD	2002-2013	1st, 2nd, 3rd	CDC
12601	Pre- and Postnatal Exposure Periods for Child Health (Rochester)	259	Rochester, NY	Clinic/hospital-based	General population<br><br>Healthy pregnant women with singletons	2016-2020	1st, 2nd	CDC
12901	Archive for Research in Child Health (ARCH)	171	Lansing, MI	Clinic/hospital-based; Convenience sample	General population<br><br>Low-income pregnant women	2008-2017	1st, 2nd, 3rd	HHEAR - Wadsworth
13101	Illinois Kids Development Study (IKIDS)	356	Urbana-Champaign, IL	Clinic/hospital-based	General population<br><br>Healthy pregnant women with singletons	2014-2022	2nd	CDC
13301	The NYU Children's Health and Environment Study (NYU CHES)	522	New York, NY	Clinic/hospital-based	General population<br><br>Pregnant women	2016-2022	1st, 2nd, 3rd	HHEAR - Wadsworth

20101	The Infant Development and the Environment Study (TIDES)	668	Minneapolis, MN<br>Rochester, NY<br>San Francisco, CA<br>Seattle, WA	Clinic/hospital-based	General population<br><br>Pregnant women	2011-2013	1st, 2nd, 3rd	HHEAR - Wadsworth
20201	Columbia Center for Children's Environmental Health (CCCEH) - Mothers and Newborns (M and N)	422	New York, NY	Clinic/hospital-based	General population<br><br>Healthy pregnant Hispanic and African-American women with singletons	1998-2006	2nd, 3rd	CDC

Supplement Table 1: Equations for calculating molar sums

Molecular Sum	Equation
Low Molecular Weight (LMW)	$\frac{MEP/1000}{194}+\frac{MNBP/1000}{222}+\frac{MIBP/1000}{222}$
High Molecular Weight (HMW)	$\frac{MCPP/1000}{252}+\frac{MECPP/1000}{308}+\frac{MEHHP/1000}{294}+\frac{MEOHP/1000}{292}+\frac{MEHP/1000}{278}+\frac{MBZP/1000}{256}$
DEHP	$\frac{MEHP/1000}{278}+\frac{MEHHP/1000}{294}+\frac{MEOHP/1000}{292}+\frac{MECPP/1000}{308}$
DEHP (alt)	$\frac{MEHP/1000}{278}+\frac{MEHHP/1000}{294}+\frac{MEOHP/1000}{292}+\frac{MECPP/1000}{308}+\frac{MCMHP/1000}{308}$
DiNP	$\frac{MINP/1000}{292}+\frac{MCIOP/1000}{322}$
DnOP	$\frac{MCPP/1000}{252}+\frac{MCHPP/1000}{308}$

Note: No equations for PA (phthalic acid) and DiDP (MCINP) since only composed of 1 analyte

Supplement Table 2a: Adjusted Associations of Phthalate Exposures with Birth Outcomes (Continuously Measured)

Models adjusted for maternal age, race/ethnicity, parity, and education, and child sex; cohort as random effect· BW for GA model removes parity and child sex from covariates·

Outcome: Gestational Age

	Across Pregnancy		Trimester 1		Trimester 2		Trimester 3	
**Exposure**	**N**	**Beta (95% CI)**	**N**	**Beta (95% CI)**	**N**	**Beta (95% CI)**	**N**	**Beta (95% CI)**
LMW	4389	-0·095 (-0·224, 0·034)	999	0·03 (-0·177, 0·237)	3124	0·039 (-0·098, 0·176)	3097	**-0·126 (-0·249, -0·002)**
HMW	4446	**-0·23 (-0·4, -0·061)**	1168	**-0·316 (-0·554, -0·078)**	3062	0·084 (-0·089, 0·256)	2831	**-0·248 (-0·426, -0·07)**
DEHP	4454	**-0·253 (-0·414, -0·091)**	1197	**-0·258 (-0·486, -0·029)**	3069	0·064 (-0·102, 0·23)	2844	**-0·287 (-0·452, -0·121)**
PA	2607	**-0·671 (-0·868, -0·475)**	908	0·056 (-0·154, 0·265)	2079	-0·124 (-0·282, 0·034)	2143	**-0·586 (-0·737, -0·435)**
DiNP	2845	**-0·485 (-0·637, -0·334)**	832	0·033 (-0·168, 0·234)	2310	-0·035 (-0·169, 0·099)	1400	**-0·533 (-0·716, -0·349)**
DnOP	2037	**-0·632 (-0·882, -0·382)**	815	0·103 (-0·117, 0·323)	1674	-0·017 (-0·228, 0·194)	1217	**-0·53 (-0·764, -0·295)**
DiDP	3775	**-0·261 (-0·407, -0·114)**	918	0·104 (-0·073, 0·282)	2970	-0·008 (-0·139, 0·123)	2568	**-0·148 (-0·286, -0·009)**

Outcome: Birth Length

	Across Pregnancy		Trimester 1		Trimester 2		Trimester 3	
**Exposure**	**N**	**Beta (95% CI)**	**N**	**Beta (95% CI)**	**N**	**Beta (95% CI)**	**N**	**Beta (95% CI)**
LMW	3721	**-0·279 (-0·511, -0·047)**	754	**-0·387 (-0·766, -0·009)**	2749	-0·008 (-0·249, 0·234)	2657	**-0·273 (-0·496, -0·051)**
HMW	3851	-0·238 (-0·539, 0·062)	959	0·062 (-0·372, 0·496)	2710	0·055 (-0·245, 0·354)	2417	**-0·334 (-0·653, -0·016)**
DEHP	3859	-0·234 (-0·52, 0·052)	984	0·058 (-0·357, 0·474)	2717	0·111 (-0·176, 0·398)	2430	**-0·314 (-0·611, -0·017)**
PA	2054	**-0·892 (-1·284, -0·501)**	691	-0·058 (-0·439, 0·323)	1764	0·106 (-0·181, 0·392)	1772	**-0·762 (-1·063, -0·461)**
DiNP	2456	**-0·562 (-0·83, -0·295)**	710	0·071 (-0·273, 0·415)	2043	-0·016 (-0·256, 0·224)	1071	**-0·65 (-0·998, -0·302)**
DnOP	1653	**-0·594 (-1·046, -0·141)**	691	0·099 (-0·291, 0·488)	1414	0·115 (-0·256, 0·487)	887	**-0·659 (-1·109, -0·209)**
DiDP	3226	-0·234 (-0·496, 0·029)	711	0·141 (-0·17, 0·453)	2646	0·074 (-0·157, 0·305)	2198	-0·159 (-0·407, 0·088)

Outcome: Birthweight

	Across Pregnancy		Trimester 1		Trimester 2		Trimester 3	
**Exposure**	**N**	**Beta (95% CI)**	**N**	**Beta (95% CI)**	**N**	**Beta (95% CI)**	**N**	**Beta (95% CI)**
LMW	4389	-26·52 (-64·069, 11·03)	999	-4·036 (-67·75, 59·677)	3124	-7·25 (-47·938, 33·437)	3097	**-41·921 (-79·536, -4·307)**
HMW	4446	-36·316 (-86·273, 13·641)	1168	24·209 (-51·129, 99·547)	3062	-2·979 (-55·346, 49·388)	2831	-37·415 (-90·984, 16·153)
DEHP	4454	-45·72 (-93·318, 1·879)	1197	20·872 (-50·835, 92·579)	3069	-2·542 (-52·824, 47·74)	2844	**-69·053 (-119·544, -18·563)**
PA	2607	**-162·867 (-220·254, -105·479)**	908	-6·859 (-72·697, 58·98)	2079	-20·457 (-72·202, 31·287)	2143	**-133·082 (-179·494, -86·67)**
DiNP	2845	**-93·494 (-137·162, -49·827)**	832	**71·873 (9·035, 134·71)**	2310	6·513 (-33·001, 46·027)	1400	**-105·904 (-153·879, -57·929)**
DnOP	2037	**-144·028 (-214·002, -74·055)**	815	29·036 (-39·589, 97·661)	1674	-1·173 (-62·483, 60·136)	1217	**-143·537 (-211·303, -75·772)**
DiDP	3775	**-51·721 (-92·991, -10·452)**	918	32·248 (-23·251, 87·748)	2970	5·969 (-33·89, 45·829)	2568	-37·63 (-79·739, 4·479)

Outcome: Birthweight for Gestational Age Z-Score

	Across Pregnancy		Trimester 1		Trimester 2		Trimester 3	
**Exposure**	**N**	**Beta (95% CI)**	**N**	**Beta (95% CI)**	**N**	**Beta (95% CI)**	**N**	**Beta (95% CI)**
LMW	4160	-0·07 (-0·148, 0·007)	810	-0·055 (-0·202, 0·091)	2916	-0·032 (-0·121, 0·057)	2881	-0·068 (-0·148, 0·012)
HMW	4217	0·018 (-0·083, 0·119)	979	0·139 (-0·033, 0·311)	2854	-0·029 (-0·14, 0·081)	2615	0·007 (-0·106, 0·119)
DEHP	4225	0·01 (-0·087, 0·106)	1008	0·126 (-0·041, 0·292)	2861	-0·001 (-0·108, 0·106)	2628	-0·025 (-0·13, 0·079)
PA	2388	-0·058 (-0·17, 0·053)	721	-0·028 (-0·181, 0·124)	1875	-0·015 (-0·121, 0·092)	1934	-0·024 (-0·118, 0·069)
DiNP	2624	0·024 (-0·062, 0·11)	644	0·135 (-0·016, 0·287)	2104	0·062 (-0·023, 0·147)	1190	-0·024 (-0·127, 0·08)
DnOP	1818	-0·039 (-0·173, 0·094)	628	0·011 (-0·153, 0·176)	1470	0 (-0·128, 0·128)	1008	-0·09 (-0·227, 0·048)
DiDP	3553	-0·014 (-0·102, 0·075)	730	-0·031 (-0·158, 0·097)	2763	0·027 (-0·061, 0·115)	2358	-0·03 (-0·116, 0·056)

Supplement Table 2b: Adjusted Associations of Phthalate Exposures with Categorical Birth Outcomes

Models adjusted for maternal age, race/ethnicity, parity, and education, and child sex; cohort as random effect

Outcome: Preterm

	Across Pregnancy		Trimester 1		Trimester 2		Trimester 3	
**Exposure**	**N**	**OR (95% CI)**	**N**	**OR (95% CI)**	**N**	**OR (95% CI)**	**N**	**OR (95% CI)**
LMW	4389	1·02 (0·77, 1·33)	999	0·89 (0·51, 1·55)	3124	0·9 (0·65, 1·25)	3097	1·1 (0·84, 1·45)
HMW	4446	**1·52 (1·08, 2·15)**	1168	1·41 (0·74, 2·7)	3062	0·82 (0·54, 1·24)	2831	**1·69 (1·18, 2·44)**
DEHP	4454	**1·45 (1·05, 2·01)**	1197	1·2 (0·65, 2·22)	3069	0·85 (0·57, 1·28)	2844	**1·72 (1·23, 2·4)**
PA	2607	**2·71 (1·91, 3·83)**	908	0·92 (0·51, 1·68)	2079	1·26 (0·84, 1·89)	2143	**2·4 (1·74, 3·32)**
DiNP	2845	**2·25 (1·69, 3)**	832	0·88 (0·5, 1·52)	2310	1·12 (0·81, 1·55)	1400	**2·35 (1·69, 3·26)**
DnOP	2037	**2·9 (1·96, 4·29)**	815	0·75 (0·4, 1·41)	1674	1·24 (0·8, 1·91)	1217	**2·48 (1·71, 3·6)**
DiDP	3775	**1·69 (1·25, 2·28)**	918	0·65 (0·39, 1·08)	2970	1 (0·73, 1·37)	2568	**1·44 (1·06, 1·96)**

Outcome: Small for Gestational Age

	Across Pregnancy		Trimester 1		Trimester 2		Trimester 3	
**Exposure**	**N**	**OR (95% CI)**	**N**	**OR (95% CI)**	**N**	**OR (95% CI)**	**N**	**OR (95% CI)**
LMW	4382	1·01 (0·81, 1·27)	998	1·08 (0·74, 1·58)	3123	0·94 (0·72, 1·21)	3091	0·9 (0·71, 1·13)
HMW	4438	1·05 (0·78, 1·43)	1166	0·78 (0·49, 1·22)	3061	1·29 (0·94, 1·78)	2825	1·03 (0·74, 1·43)
DEHP	4446	1·07 (0·8, 1·43)	1195	0·79 (0·51, 1·22)	3068	1·21 (0·89, 1·64)	2838	1·01 (0·75, 1·37)
PA	2607	1·06 (0·75, 1·51)	908	1·2 (0·8, 1·8)	2079	1·09 (0·8, 1·49)	2143	1·02 (0·76, 1·36)
DiNP	2845	1·03 (0·79, 1·34)	832	0·73 (0·5, 1·07)	2310	1 (0·77, 1·31)	1400	1·09 (0·8, 1·47)
DnOP	2037	1·05 (0·71, 1·56)	815	0·89 (0·59, 1·35)	1674	0·95 (0·66, 1·37)	1217	1·26 (0·88, 1·81)
DiDP	3775	1·04 (0·8, 1·35)	918	1·09 (0·79, 1·5)	2970	0·97 (0·76, 1·24)	2568	1·01 (0·8, 1·28)

Outcome: Large for Gestational Age

	Across Pregnancy		Trimester 1		Trimester 2		Trimester 3	
**Exposure**	**N**	**OR (95% CI)**	**N**	**OR (95% CI)**	**N**	**OR (95% CI)**	**N**	**OR (95% CI)**
LMW	4382	0·96 (0·75, 1·23)	998	1·17 (0·77, 1·79)	3123	0·93 (0·7, 1·22)	3091	1·02 (0·8, 1·3)
HMW	4438	0·95 (0·69, 1·3)	1166	1·54 (0·94, 2·52)	3061	0·82 (0·58, 1·16)	2825	0·91 (0·64, 1·29)
DEHP	4446	0·92 (0·68, 1·24)	1195	1·53 (0·96, 2·44)	3068	0·83 (0·6, 1·16)	2838	0·82 (0·59, 1·15)
PA	2607	1·16 (0·86, 1·57)	908	1·12 (0·74, 1·71)	2079	1·33 (0·98, 1·8)	2143	1·01 (0·78, 1·31)
DiNP	2845	1·08 (0·83, 1·42)	832	**1·46 (1·01, 2·11)**	2310	1·11 (0·85, 1·44)	1400	0·89 (0·67, 1·19)
DnOP	2037	0·97 (0·64, 1·47)	815	0·94 (0·61, 1·46)	1674	0·91 (0·61, 1·36)	1217	0·88 (0·6, 1·28)
DiDP	3775	1·2 (0·91, 1·57)	918	1·41 (0·98, 2·02)	2970	1·08 (0·84, 1·41)	2568	1·12 (0·86, 1·48)

Outcome: Low Birthweight

	Across Pregnancy		Trimester 1		Trimester 2		Trimester 3	
**Exposure**	**N**	**OR (95% CI)**	**N**	**OR (95% CI)**	**N**	**OR (95% CI)**	**N**	**OR (95% CI)**
LMW	4389	1·26 (0·94, 1·69)	999	1 (0·57, 1·76)	3124	1·02 (0·71, 1·45)	3097	1·16 (0·87, 1·57)
HMW	4446	**1·52 (1·03, 2·24)**	1168	1·4 (0·72, 2·73)	3062	0·95 (0·61, 1·5)	2831	1·34 (0·89, 2·01)
DEHP	4454	**1·55 (1·08, 2·23)**	1197	1·27 (0·68, 2·36)	3069	0·97 (0·63, 1·51)	2844	**1·55 (1·07, 2·25)**
PA	2607	**2·78 (1·91, 4·04)**	908	1·25 (0·68, 2·33)	2079	1·42 (0·91, 2·21)	2143	**2·07 (1·47, 2·92)**
DiNP	2845	**2·13 (1·55, 2·93)**	832	0·76 (0·41, 1·39)	2310	1·01 (0·7, 1·46)	1400	**1·99 (1·4, 2·82)**
DnOP	2037	**2·05 (1·36, 3·11)**	815	0·68 (0·35, 1·33)	1674	0·86 (0·54, 1·39)	1217	**1·99 (1·34, 2·94)**
DiDP	3775	**1·65 (1·19, 2·29)**	918	0·89 (0·55, 1·45)	2970	0·91 (0·63, 1·3)	2568	1·3 (0·94, 1·8)

Outcome: Low Birthweight (Preterms Only)

	Across Pregnancy		Trimester 1		Trimester 2		Trimester 3	
Exposure	N	OR (95% CI)	N	OR (95% CI)	N	OR (95% CI)	N	OR (95% CI)
LMW	345	1·25 (0·75, 2·11)	60	1·21 (0·31, 4·75)	219	1·23 (0·61, 2·48)	230	0·92 (0·55, 1·56)
HMW	344	0·91 (0·42, 1·96)	59	0·26 (0·03, 2·37)	216	0·78 (0·29, 2·13)	217	0·88 (0·41, 1·87)
DEHP	346	0·91 (0·44, 1·88)	66	0·19 (0·03, 1·06)	217	0·75 (0·27, 2·1)	218	1·14 (0·56, 2·31)
PA	226	1·61 (0·91, 2·84)	49	0·95 (0·21, 4·24)	144	0·87 (0·36, 2·12)	169	1·66 (0·93, 2·98)
DiNP	233	1·47 (0·8, 2·71)	42	0·45 (0·06, 3·35)	159	1·08 (0·49, 2·39)	128	1·1 (0·59, 2·04)
DnOP	191	0·96 (0·46, 2·02)	42	0·3 (0·03, 2·86)	122	0·5 (0·19, 1·28)	123	1·14 (0·52, 2·5)
DiDP	295	1·33 (0·75, 2·33)	48	1·09 (0·26, 4·56)	205	0·99 (0·5, 1·95)	191	0·78 (0·42, 1·45)

Outcome: Low Birthweight (Terms only)

	Across Pregnancy		Trimester 1		Trimester 2		Trimester 3	
Exposure	N	OR (95% CI)	N	OR (95% CI)	N	OR (95% CI)	N	OR (95% CI)
LMW	4044	1·13 (0·7, 1·81)	939	1·21 (0·58, 2·53)	2905	0·95 (0·56, 1·62)	2867	1·04 (0·66, 1·66)
HMW	4102	1·2 (0·65, 2·2)	1109	1·76 (0·78, 3·99)	2846	1·04 (0·57, 1·92)	2614	0·91 (0·48, 1·72)
DEHP	4108	1·41 (0·81, 2·46)	1131	**2·15 (1·03, 4·48)**	2852	1·13 (0·64, 1·98)	2626	1·15 (0·65, 2·04)
PA	2381	1·81 (0·91, 3·57)	859	1·61 (0·72, 3·62)	1935	1·87 (0·97, 3·64)	1974	0·97 (0·55, 1·71)
DiNP	2612	1·09 (0·66, 1·82)	790	0·64 (0·29, 1·38)	2151	0·89 (0·52, 1·53)	1272	1·07 (0·63, 1·79)
DnOP	1846	1·21 (0·57, 2·59)	773	0·67 (0·29, 1·54)	1552	0·77 (0·36, 1·68)	1094	1·38 (0·7, 2·71)
DiDP	3480	1·11 (0·67, 1·83)	870	1·14 (0·6, 2·16)	2765	0·71 (0·44, 1·14)	2377	1·26 (0·79, 2·02)

Supplement Table 3a1: Sex-Stratified Adjusted Associations of Phthalate Exposures with Birth Outcomes (Continuously Measured), Male Children Only

Models adjusted for maternal age, race/ethnicity, parity, and education· BW for GA model removes parity from covariates·

Outcome: Gestational Age

	Across Pregnancy		Trimester 1		Trimester 2		Trimester 3	
**Exposure**	**N**	**Beta (95% CI)**	**N**	**Beta (95% CI)**	**N**	**Beta (95% CI)**	**N**	**Beta (95% CI)**
LMW	2227	-0·12 (-0·295, 0·054)	506	-0·26 (-0·57, 0·051)	1580	-0·162 (-0·349, 0·024)	1563	-0·082 (-0·252, 0·088)
HMW	2231	-0·141 (-0·355, 0·073)	574	-0·244 (-0·593, 0·106)	1537	0·078 (-0·141, 0·296)	1432	-0·161 (-0·398, 0·076)
DEHP	2237	-0·111 (-0·314, 0·092)	594	-0·276 (-0·617, 0·065)	1542	0·059 (-0·148, 0·265)	1440	-0·17 (-0·39, 0·05)
PA	1306	**-0·653 (-0·908, -0·399)**	453	0·147 (-0·152, 0·446)	1035	-0·111 (-0·335, 0·113)	1075	**-0·532 (-0·73, -0·334)**
DiNP	1436	**-0·433 (-0·624, -0·241)**	417	0·121 (-0·173, 0·415)	1145	-0·031 (-0·206, 0·145)	715	**-0·611 (-0·84, -0·382)**
DnOP	1031	**-0·493 (-0·861, -0·126)**	408	0·194 (-0·131, 0·518)	828	-0·004 (-0·306, 0·299)	618	**-0·569 (-0·915, -0·223)**
DiDP	1912	**-0·209 (-0·405, -0·013)**	457	**0·303 (0·039, 0·568)**	1492	0·071 (-0·103, 0·245)	1301	**-0·241 (-0·423, -0·06)**

Outcome: Birth Length

	Across Pregnancy		Trimester 1		Trimester 2		Trimester 3	
**Exposure**	**N**	**Beta (95% CI)**	**N**	**Beta (95% CI)**	**N**	**Beta (95% CI)**	**N**	**Beta (95% CI)**
LMW	1891	-0·181 (-0·497, 0·134)	388	-0·496 (-1·086, 0·093)	1401	-0·148 (-0·472, 0·177)	1362	-0·12 (-0·43, 0·19)
HMW	1942	-0·135 (-0·509, 0·239)	479	-0·06 (-0·67, 0·549)	1376	-0·003 (-0·377, 0·371)	1244	-0·079 (-0·504, 0·345)
DEHP	1948	-0·036 (-0·393, 0·32)	496	-0·22 (-0·806, 0·365)	1381	0·059 (-0·294, 0·412)	1252	-0·097 (-0·491, 0·296)
PA	1037	**-0·914 (-1·429, -0·399)**	352	0·108 (-0·421, 0·638)	890	0·025 (-0·383, 0·433)	912	**-0·918 (-1·316, -0·519)**
DiNP	1254	**-0·837 (-1·171, -0·504)**	363	-0·063 (-0·568, 0·441)	1030	-0·189 (-0·494, 0·115)	573	**-1·075 (-1·501, -0·65)**
DnOP	850	-0·46 (-1·135, 0·215)	352	-0·039 (-0·597, 0·519)	716	0·159 (-0·369, 0·688)	476	**-0·933 (-1·602, -0·264)**
DiDP	1645	**-0·538 (-0·909, -0·167)**	363	0·281 (-0·175, 0·738)	1340	0·048 (-0·263, 0·359)	1138	**-0·566 (-0·894, -0·237)**

Outcome: Birthweight

	Across Pregnancy		Trimester 1		Trimester 2		Trimester 3	
**Exposure**	**N**	**Beta (95% CI)**	**N**	**Beta (95% CI)**	**N**	**Beta (95% CI)**	**N**	**Beta (95% CI)**
LMW	2227	-42·114 (-93·107, 8·879)	506	-17·304 (-115·974, 81·366)	1580	**-64·699 (-121·401, -7·997)**	1563	-19·965 (-71·779, 31·85)
HMW	2231	-17·716 (-80·046, 44·614)	574	8·395 (-103·281, 120·072)	1537	-51·984 (-118·259, 14·291)	1432	-4·057 (-75·438, 67·324)
DEHP	2237	-20·911 (-80·09, 38·267)	594	-33·124 (-141·414, 75·165)	1542	-45·094 (-107·817, 17·629)	1440	-22·729 (-88·967, 43·509)
PA	1306	**-143·412 (-218·168, -68·656)**	453	65·305 (-30·671, 161·282)	1035	-25·723 (-94·152, 42·706)	1075	**-130·788 (-191·952, -69·624)**
DiNP	1436	**-89·16 (-144·864, -33·456)**	417	83·173 (-10·698, 177·045)	1145	-0·518 (-53·746, 52·71)	715	**-131·015 (-198·173, -63·857)**
DnOP	1031	**-106·172 (-209·306, -3·037)**	408	75·383 (-28·59, 179·356)	828	19·629 (-69·004, 108·263)	618	**-160·323 (-261·879, -58·766)**
DiDP	1912	-21·933 (-79·75, 35·884)	457	**109·349 (24·58, 194·118)**	1492	10·73 (-42·556, 64·015)	1301	-19·864 (-76·071, 36·343)

Outcome: Birthweight for Gestational Age Z-Score

	Across Pregnancy		Trimester 1		Trimester 2		Trimester 3	
**Exposure**	**N**	**Beta (95% CI)**	**N**	**Beta (95% CI)**	**N**	**Beta (95% CI)**	**N**	**Beta (95% CI)**
LMW	2120	-0·052 (-0·155, 0·051)	419	-0·03 (-0·248, 0·187)	1482	-0·093 (-0·211, 0·026)	1464	-0·012 (-0·122, 0·098)
HMW	2123	0·022 (-0·103, 0·146)	486	0·187 (-0·06, 0·434)	1439	**-0·189 (-0·325, -0·054)**	1333	0·101 (-0·044, 0·246)
DEHP	2129	0·002 (-0·117, 0·121)	506	0·123 (-0·119, 0·366)	1444	**-0·159 (-0·288, -0·029)**	1341	0·047 (-0·088, 0·183)
PA	1204	-0·004 (-0·145, 0·137)	367	0·202 (-0·008, 0·412)	939	0·015 (-0·125, 0·154)	979	-0·03 (-0·151, 0·092)
DiNP	1333	-0·009 (-0·114, 0·096)	330	**0·231 (0·02, 0·442)**	1048	-0·005 (-0·112, 0·102)	619	-0·007 (-0·134, 0·119)
DnOP	929	-0·028 (-0·216, 0·161)	322	0·055 (-0·192, 0·301)	732	-0·012 (-0·187, 0·163)	522	-0·106 (-0·305, 0·092)
DiDP	1808	0·032 (-0·083, 0·147)	370	0·061 (-0·126, 0·248)	1394	-0·029 (-0·141, 0·083)	1205	0·067 (-0·045, 0·179)

Supplement Table 3b1: Sex-Stratified Adjusted Associations of Phthalate Exposures with Categorical Birth Outcomes, Male Children Only

Models adjusted for maternal age, race/ethnicity, parity, and education·

Outcome: Preterm

	Across Pregnancy		Trimester 1		Trimester 2		Trimester 3	
**Exposure**	**N**	**OR (95% CI)**	**N**	**OR (95% CI)**	**N**	**OR (95% CI)**	**N**	**OR (95% CI)**
LMW	2227	0·98 (0·69, 1·39)	506	0·92 (0·43, 1·98)	1580	0·96 (0·62, 1·48)	1563	1·11 (0·77, 1·59)
HMW	2231	1·23 (0·82, 1·87)	574	0·9 (0·35, 2·33)	1537	0·7 (0·42, 1·16)	1432	**1·64 (1·03, 2·6)**
DEHP	2237	1·08 (0·73, 1·6)	594	0·84 (0·35, 2)	1542	0·71 (0·43, 1·15)	1440	1·52 (1, 2·33)
PA	1306	**2·82 (1·8, 4·42)**	453	0·84 (0·4, 1·78)	1035	1·21 (0·72, 2·04)	1075	**2·81 (1·79, 4·41)**
DiNP	1436	**1·84 (1·31, 2·59)**	417	0·79 (0·36, 1·72)	1145	0·87 (0·58, 1·31)	715	**2·54 (1·72, 3·77)**
DnOP	1031	**2·26 (1·3, 3·91)**	408	0·49 (0·2, 1·24)	828	1·09 (0·6, 2)	618	**2·82 (1·66, 4·77)**
DiDP	1912	1·35 (0·91, 2)	457	**0·34 (0·16, 0·72)**	1492	0·89 (0·59, 1·35)	1301	**1·56 (1·07, 2·28)**

Outcome: Small for Gestational Age

	Across Pregnancy		Trimester 1		Trimester 2		Trimester 3	
**Exposure**	**N**	**OR (95% CI)**	**N**	**OR (95% CI)**	**N**	**OR (95% CI)**	**N**	**OR (95% CI)**
LMW	2224	0·9 (0·66, 1·22)	506	0·99 (0·54, 1·79)	1580	0·92 (0·64, 1·32)	1560	0·82 (0·6, 1·11)
HMW	2227	1·01 (0·7, 1·47)	573	0·78 (0·38, 1·58)	1537	**1·51 (1·02, 2·25)**	1429	0·95 (0·63, 1·44)
DEHP	2233	1·02 (0·71, 1·45)	593	0·84 (0·43, 1·64)	1542	1·36 (0·94, 1·99)	1437	0·94 (0·64, 1·37)
PA	1306	0·96 (0·63, 1·46)	453	0·97 (0·54, 1·74)	1035	1·02 (0·69, 1·53)	1075	1·16 (0·8, 1·69)
DiNP	1436	1·11 (0·8, 1·55)	417	0·81 (0·44, 1·48)	1145	1·17 (0·84, 1·62)	715	1·19 (0·81, 1·75)
DnOP	1031	1·06 (0·6, 1·87)	408	0·8 (0·41, 1·54)	828	1 (0·6, 1·68)	618	1·49 (0·88, 2·53)
DiDP	1912	0·89 (0·62, 1·27)	457	0·97 (0·58, 1·63)	1492	1·03 (0·73, 1·45)	1301	0·91 (0·65, 1·25)

Outcome: Large for Gestational Age

	Across Pregnancy		Trimester 1		Trimester 2		Trimester 3	
**Exposure**	**N**	**OR (95% CI)**	**N**	**OR (95% CI)**	**N**	**OR (95% CI)**	**N**	**OR (95% CI)**
LMW	2224	0·8 (0·57, 1·13)	506	1·07 (0·59, 1·93)	1580	0·76 (0·52, 1·12)	1560	0·92 (0·65, 1·29)
HMW	2227	0·92 (0·61, 1·38)	573	1·15 (0·58, 2·26)	1537	0·65 (0·41, 1·01)	1429	1·08 (0·68, 1·73)
DEHP	2233	0·81 (0·55, 1·2)	593	0·94 (0·48, 1·82)	1542	**0·64 (0·42, 0·99)**	1437	0·9 (0·58, 1·42)
PA	1306	0·97 (0·63, 1·48)	453	1·39 (0·78, 2·47)	1035	1·14 (0·74, 1·74)	1075	0·93 (0·66, 1·31)
DiNP	1436	1·05 (0·75, 1·46)	417	**1·73 (1·04, 2·87)**	1145	1·1 (0·79, 1·53)	715	0·92 (0·62, 1·35)
DnOP	1031	0·96 (0·53, 1·72)	408	1·09 (0·61, 1·95)	828	1·01 (0·59, 1·73)	618	0·88 (0·51, 1·53)
DiDP	1912	1·27 (0·88, 1·82)	457	**1·77 (1·08, 2·92)**	1492	1·17 (0·83, 1·65)	1301	1·22 (0·85, 1·77)

Outcome: Low Birthweight

	Across Pregnancy		Trimester 1		Trimester 2		Trimester 3	
**Exposure**	**N**	**OR (95% CI)**	**N**	**OR (95% CI)**	**N**	**OR (95% CI)**	**N**	**OR (95% CI)**
LMW	2227	1·26 (0·83, 1·92)	506	1·74 (0·75, 4·03)	1580	1·15 (0·68, 1·95)	1563	1·11 (0·72, 1·7)
HMW	2231	1·58 (0·96, 2·6)	574	1·67 (0·65, 4·3)	1537	1·22 (0·67, 2·23)	1432	1·19 (0·68, 2·11)
DEHP	2237	1·37 (0·86, 2·2)	594	1·6 (0·67, 3·79)	1542	1·02 (0·57, 1·82)	1440	1·29 (0·77, 2·15)
PA	1306	**2·89 (1·75, 4·77)**	453	1·05 (0·46, 2·42)	1035	1·37 (0·76, 2·48)	1075	**3·44 (2·07, 5·73)**
DiNP	1436	**2·2 (1·48, 3·28)**	417	0·82 (0·32, 2·13)	1145	1·01 (0·62, 1·66)	715	**2·62 (1·67, 4·1)**
DnOP	1031	**2·57 (1·37, 4·81)**	408	0·33 (0·11, 1·05)	828	1·08 (0·54, 2·19)	618	**2·84 (1·59, 5·05)**
DiDP	1912	1·46 (0·9, 2·35)	457	**0·46 (0·21, 0·99)**	1492	0·9 (0·54, 1·5)	1301	**1·63 (1·05, 2·52)**

Outcome: Low Birthweight (Preterms only)

	Across Pregnancy		Trimester 1		Trimester 2		Trimester 3	
**Exposure**	**N**	**OR (95% CI)**	**N**	**OR (95% CI)**	**N**	**OR (95% CI)**	**N**	**OR (95% CI)**
LMW	188	1·37 (0·71, 2·62)	37	5·73 (0·45, 73·23)	116	1·39 (0·53, 3·61)	123	0·87 (0·43, 1·74)
HMW	186	1·85 (0·8, 4·28)	33	34·92 (0·55, 2221·7)	115	**4 (1·1, 14·51)**	116	0·7 (0·27, 1·81)
DEHP	188	1·63 (0·74, 3·62)	39	4·4 (0·24, 81·51)	116	2·85 (0·88, 9·28)	117	0·92 (0·37, 2·28)
PA	121	1·84 (0·8, 4·24)	29	2·42 (0·41, 14·19)	76	1·27 (0·33, 4·92)	88	**2·64 (1·07, 6·51)**
DiNP	125	**2·79 (1·37, 5·66)**	25	2·96 (0·33, 26·32)	82	1·62 (0·56, 4·66)	68	1·53 (0·76, 3·11)
DnOP	107	1·96 (0·59, 6·55)	25	5·87 (0·26, 131·92)	67	1·24 (0·3, 5·13)	65	1·35 (0·48, 3·78)
DiDP	159	1·45 (0·71, 2·93)	28	1·94 (0·18, 21)	107	1·04 (0·41, 2·63)	102	1·14 (0·56, 2·32)

Outcome: Low Birthweight (Terms only)

	Across Pregnancy		Trimester 1		Trimester 2		Trimester 3	
**Exposure**	**N**	**OR (95% CI)**	**N**	**OR (95% CI)**	**N**	**OR (95% CI)**	**N**	**OR (95% CI)**
LMW	2039	1·19 (0·57, 2·47)	469	2·15 (0·63, 7·33)	1464	1·16 (0·47, 2·85)	1440	1·05 (0·51, 2·17)
HMW	2045	1·36 (0·58, 3·2)	541	1·5 (0·39, 5·79)	1422	1·25 (0·45, 3·42)	1316	1·02 (0·38, 2·72)
DEHP	2049	1·45 (0·67, 3·16)	555	2·19 (0·72, 6·63)	1426	1·16 (0·45, 3)	1323	1·15 (0·49, 2·69)
PA	1185	1·05 (0·37, 3)	424	0·81 (0·25, 2·66)	959	1·49 (0·55, 4·03)	987	1·38 (0·51, 3·72)
DiNP	1311	0·94 (0·41, 2·19)	392	0·36 (0·07, 1·91)	1063	0·82 (0·36, 1·85)	647	1·31 (0·48, 3·53)
DnOP	924	1·53 (0·39, 6·07)	383	**0·07 (0·01, 0·61)**	761	0·97 (0·27, 3·47)	553	2·56 (0·78, 8·36)
DiDP	1753	1 (0·4, 2·53)	429	0·57 (0·17, 1·91)	1385	0·71 (0·3, 1·68)	1199	1·37 (0·62, 3·07)

Supplement Table 3a2: Sex-Stratified Adjusted Associations of Phthalate Exposures with Birth Outcomes (Continuously Measured), Female Children Only

Models adjusted for maternal age, race/ethnicity, parity, and education· BW for GA model removes parity from covariates·

Outcome: Gestational Age

	Across Pregnancy		Trimester 1		Trimester 2		Trimester 3	
**Exposure**	**N**	**Beta (95% CI)**	**N**	**Beta (95% CI)**	**N**	**Beta (95% CI)**	**N**	**Beta (95% CI)**
LMW	2162	0·082 (-0·088, 0·252)	493	0·093 (-0·174, 0·36)	1544	**0·257 (0·073, 0·441)**	1534	-0·007 (-0·178, 0·164)
HMW	2215	-0·165 (-0·371, 0·042)	594	**-0·376 (-0·688, -0·064)**	1525	0·16 (-0·057, 0·377)	1399	-0·128 (-0·359, 0·104)
DEHP	2217	-0·186 (-0·38, 0·009)	603	**-0·317 (-0·616, -0·017)**	1527	0·121 (-0·085, 0·327)	1404	-0·138 (-0·353, 0·076)
PA	1301	**-0·693 (-0·947, -0·439)**	455	-0·074 (-0·364, 0·217)	1044	-0·128 (-0·351, 0·094)	1068	**-0·52 (-0·736, -0·303)**
DiNP	1409	**-0·492 (-0·674, -0·309)**	415	-0·042 (-0·296, 0·212)	1165	-0·009 (-0·18, 0·163)	685	**-0·514 (-0·737, -0·291)**
DnOP	1006	**-0·863 (-1·208, -0·517)**	407	-0·06 (-0·358, 0·237)	846	0 (-0·293, 0·294)	599	**-0·621 (-0·949, -0·292)**
DiDP	1863	**-0·35 (-0·533, -0·166)**	461	-0·07 (-0·301, 0·162)	1478	-0·056 (-0·23, 0·118)	1267	**-0·256 (-0·431, -0·082)**

Outcome: Birth Length

	Across Pregnancy		Trimester 1		Trimester 2		Trimester 3	
**Exposure**	**N**	**Beta (95% CI)**	**N**	**Beta (95% CI)**	**N**	**Beta (95% CI)**	**N**	**Beta (95% CI)**
LMW	1830	0·058 (-0·251, 0·367)	366	-0·265 (-0·751, 0·221)	1348	0·327 (-0·001, 0·654)	1295	0·052 (-0·259, 0·362)
HMW	1909	-0·103 (-0·471, 0·264)	480	0·065 (-0·529, 0·66)	1334	**0·424 (0·045, 0·802)**	1173	-0·049 (-0·469, 0·37)
DEHP	1911	-0·079 (-0·426, 0·268)	488	0·123 (-0·448, 0·694)	1336	**0·437 (0·078, 0·796)**	1178	0·07 (-0·319, 0·459)
PA	1017	**-0·822 (-1·314, -0·33)**	339	-0·242 (-0·776, 0·292)	874	0·161 (-0·239, 0·561)	860	**-0·428 (-0·852, -0·005)**
DiNP	1202	**-0·866 (-1·184, -0·547)**	347	0·06 (-0·404, 0·525)	1013	-0·013 (-0·31, 0·285)	498	**-0·938 (-1·354, -0·522)**
DnOP	803	**-1·171 (-1·797, -0·546)**	339	0·161 (-0·371, 0·693)	698	0·044 (-0·475, 0·564)	411	**-0·786 (-1·426, -0·146)**
DiDP	1581	**-0·7 (-1·041, -0·359)**	348	-0·019 (-0·438, 0·399)	1306	0·042 (-0·262, 0·347)	1060	**-0·758 (-1·067, -0·449)**

Outcome: Birthweight

	Across Pregnancy		Trimester 1		Trimester 2		Trimester 3	
**Exposure**	**N**	**Beta (95% CI)**	**N**	**Beta (95% CI)**	**N**	**Beta (95% CI)**	**N**	**Beta (95% CI)**
LMW	2162	10·137 (-39·147, 59·421)	493	-9·24 (-89·53, 71·051)	1544	47·215 (-7·876, 102·306)	1534	-7·207 (-57·214, 42·8)
HMW	2215	39·264 (-20·449, 98·977)	594	67·071 (-27·637, 161·779)	1525	**75·935 (10·847, 141·024)**	1399	22·675 (-44·718, 90·068)
DEHP	2217	31·314 (-25·048, 87·676)	603	80·267 (-10·605, 171·139)	1527	**64·567 (2·671, 126·463)**	1404	15·506 (-46·9, 77·912)
PA	1301	**-124·926 (-196·407, -53·445)**	455	-67·076 (-155·351, 21·199)	1044	-6·409 (-71·111, 58·293)	1068	**-77·339 (-140·336, -14·343)**
DiNP	1409	**-72·184 (-124·063, -20·306)**	415	60·129 (-16·971, 137·229)	1165	39·907 (-10·687, 90·501)	685	**-77·154 (-139·527, -14·782)**
DnOP	1006	**-181·169 (-275·67, -86·669)**	407	-26·592 (-116·657, 63·474)	846	-17·949 (-102·556, 66·658)	599	**-122·988 (-212·568, -33·408)**
DiDP	1863	**-81·17 (-134·584, -27·757)**	461	-22·187 (-93·061, 48·688)	1478	21·831 (-30·323, 73·985)	1267	**-83·748 (-134·867, -32·628)**

Outcome: Birthweight for Gestational Age Z-Score

	Across Pregnancy		Trimester 1		Trimester 2		Trimester 3	
**Exposure**	**N**	**Beta (95% CI)**	**N**	**Beta (95% CI)**	**N**	**Beta (95% CI)**	**N**	**Beta (95% CI)**
LMW	2040	-0·002 (-0·107, 0·102)	391	-0·052 (-0·246, 0·141)	1434	-0·005 (-0·129, 0·119)	1417	-0·007 (-0·114, 0·101)
HMW	2093	**0·19 (0·065, 0·314)**	492	0·198 (-0·022, 0·417)	1415	0·141 (-0·001, 0·283)	1282	**0·152 (0·008, 0·296)**
DEHP	2095	**0·18 (0·061, 0·298)**	501	0·196 (-0·02, 0·412)	1417	**0·142 (0·005, 0·278)**	1287	0·13 (-0·004, 0·264)
PA	1184	0·011 (-0·132, 0·155)	354	-0·161 (-0·376, 0·053)	936	0·032 (-0·109, 0·173)	955	0·06 (-0·071, 0·191)
DiNP	1291	0·079 (-0·027, 0·186)	314	0·175 (-0·006, 0·357)	1056	**0·142 (0·031, 0·253)**	571	0·075 (-0·055, 0·206)
DnOP	889	-0·02 (-0·208, 0·169)	306	0·004 (-0·218, 0·225)	738	0·007 (-0·177, 0·192)	486	0·008 (-0·182, 0·198)
DiDP	1745	-0·052 (-0·166, 0·063)	360	-0·045 (-0·209, 0·119)	1369	0·091 (-0·03, 0·212)	1153	**-0·121 (-0·229, -0·013)**

Supplement Table 3b2: Sex-Stratified Adjusted Associations of Phthalate Exposures with Categorical Birth Outcomes, Female Children Only

Models adjusted for maternal age, race/ethnicity, parity, and education·

Outcome: Preterm

	Across Pregnancy		Trimester 1		Trimester 2		Trimester 3	
**Exposure**	**N**	**OR (95% CI)**	**N**	**OR (95% CI)**	**N**	**OR (95% CI)**	**N**	**OR (95% CI)**
LMW	2162	0·84 (0·58, 1·23)	493	0·84 (0·36, 1·96)	1544	0·64 (0·4, 1·03)	1534	0·85 (0·57, 1·27)
HMW	2215	1·53 (0·99, 2·37)	594	2·42 (0·94, 6·24)	1525	0·64 (0·38, 1·1)	1399	1·52 (0·93, 2·48)
DEHP	2217	**1·52 (1·01, 2·28)**	603	1·92 (0·79, 4·67)	1527	0·66 (0·4, 1·11)	1404	1·48 (0·95, 2·31)
PA	1301	**2·4 (1·48, 3·89)**	455	1·07 (0·38, 2·98)	1044	0·91 (0·52, 1·58)	1068	**1·95 (1·21, 3·15)**
DiNP	1409	**2·43 (1·7, 3·46)**	415	1·2 (0·53, 2·74)	1165	1 (0·66, 1·51)	685	**2·01 (1·36, 2·98)**
DnOP	1006	**4·84 (2·75, 8·54)**	407	1·27 (0·51, 3·16)	846	1·44 (0·76, 2·72)	599	**2·72 (1·62, 4·57)**
DiDP	1863	**1·84 (1·25, 2·72)**	461	1·41 (0·67, 2·98)	1478	0·85 (0·56, 1·3)	1267	**1·84 (1·26, 2·71)**

Outcome: Small for Gestational Age

	Across Pregnancy		Trimester 1		Trimester 2		Trimester 3	
**Exposure**	**N**	**OR (95% CI)**	**N**	**OR (95% CI)**	**N**	**OR (95% CI)**	**N**	**OR (95% CI)**
LMW	2158	0·95 (0·7, 1·29)	492	1·15 (0·71, 1·89)	1543	0·91 (0·64, 1·3)	1531	0·86 (0·62, 1·18)
HMW	2211	0·73 (0·5, 1·07)	593	0·79 (0·44, 1·44)	1524	0·89 (0·58, 1·35)	1396	0·81 (0·53, 1·24)
DEHP	2213	0·76 (0·53, 1·08)	602	0·76 (0·43, 1·35)	1526	0·87 (0·58, 1·3)	1401	0·83 (0·56, 1·23)
PA	1301	0·93 (0·6, 1·43)	455	1·43 (0·82, 2·48)	1044	0·96 (0·63, 1·44)	1068	0·76 (0·52, 1·13)
DiNP	1409	0·89 (0·64, 1·23)	415	0·67 (0·41, 1·09)	1165	0·75 (0·53, 1·05)	685	0·89 (0·62, 1·27)
DnOP	1006	0·99 (0·57, 1·72)	407	0·94 (0·55, 1·6)	846	0·91 (0·55, 1·52)	599	0·96 (0·58, 1·59)
DiDP	1863	1·07 (0·77, 1·48)	461	1·17 (0·77, 1·78)	1478	0·86 (0·62, 1·18)	1267	1·1 (0·8, 1·5)

Outcome: Large for Gestational Age

	Across Pregnancy		Trimester 1		Trimester 2		Trimester 3	
**Exposure**	**N**	**OR (95% CI)**	**N**	**OR (95% CI)**	**N**	**OR (95% CI)**	**N**	**OR (95% CI)**
LMW	2158	1·23 (0·91, 1·67)	492	1·17 (0·64, 2·12)	1543	0·99 (0·69, 1·42)	1531	1·29 (0·94, 1·77)
HMW	2211	1·38 (0·95, 2·01)	593	**2·52 (1·26, 5)**	1524	1·03 (0·68, 1·57)	1396	1·18 (0·77, 1·8)
DEHP	2213	**1·43 (1·01, 2·02)**	602	**2·86 (1·49, 5·5)**	1526	1·03 (0·69, 1·54)	1401	1·17 (0·79, 1·73)
PA	1301	1·46 (0·94, 2·24)	455	0·9 (0·49, 1·66)	1044	**1·59 (1·03, 2·48)**	1068	1·18 (0·79, 1·75)
DiNP	1409	1·06 (0·77, 1·47)	415	1·34 (0·79, 2·26)	1165	1·12 (0·8, 1·57)	685	0·9 (0·61, 1·32)
DnOP	1006	1·01 (0·56, 1·84)	407	0·79 (0·4, 1·55)	846	0·81 (0·44, 1·48)	599	0·93 (0·55, 1·57)
DiDP	1863	1 (0·71, 1·4)	461	1·17 (0·7, 1·97)	1478	1·01 (0·72, 1·42)	1267	0·98 (0·69, 1·38)

Outcome: Low Birthweight

	Across Pregnancy		Trimester 1		Trimester 2		Trimester 3	
**Exposure**	**N**	**OR (95% CI)**	**N**	**OR (95% CI)**	**N**	**OR (95% CI)**	**N**	**OR (95% CI)**
LMW	2162	0·97 (0·66, 1·43)	493	0·74 (0·37, 1·48)	1544	0·87 (0·55, 1·39)	1534	0·87 (0·58, 1·3)
HMW	2215	1 (0·62, 1·6)	594	1·09 (0·44, 2·71)	1525	0·69 (0·4, 1·19)	1399	1 (0·6, 1·69)
DEHP	2217	1·1 (0·71, 1·7)	603	1·01 (0·42, 2·41)	1527	0·81 (0·48, 1·36)	1404	1·12 (0·7, 1·79)
PA	1301	**2·24 (1·37, 3·66)**	455	1·66 (0·68, 4·08)	1044	1·04 (0·61, 1·79)	1068	1·16 (0·73, 1·86)
DiNP	1409	**1·71 (1·18, 2·48)**	415	0·69 (0·32, 1·48)	1165	0·83 (0·54, 1·27)	685	1·48 (1, 2·2)
DnOP	1006	**1·95 (1·1, 3·47)**	407	1·13 (0·51, 2·49)	846	0·69 (0·35, 1·34)	599	1·53 (0·9, 2·61)
DiDP	1863	**1·62 (1·08, 2·42)**	461	1·39 (0·72, 2·66)	1478	0·81 (0·53, 1·23)	1267	**1·47 (1, 2·15)**

Outcome: Low Birthweight (Preterms only)

	Across Pregnancy		Trimester 1		Trimester 2		Trimester 3	
**Exposure**	**N**	**OR (95% CI)**	**N**	**OR (95% CI)**	**N**	**OR (95% CI)**	**N**	**OR (95% CI)**
LMW	157	1·31 (0·66, 2·57)	23	0·68 (0·04, 11·12)	103	1·71 (0·72, 4·09)	107	0·96 (0·46, 2·01)
HMW	158	0·46 (0·17, 1·21)			101	0·51 (0·15, 1·68)	101	0·85 (0·31, 2·35)
DEHP	158	0·58 (0·24, 1·38)	27	0 (0, 17937·78)	101	0·75 (0·24, 2·35)	101	0·94 (0·38, 2·37)
PA	105	1·32 (0·59, 2·94)			68	0·55 (0·15, 2·07)	81	0·98 (0·42, 2·28)
DiNP	108	1·09 (0·56, 2·14)			77	0·64 (0·26, 1·62)	60	1·01 (0·46, 2·2)
DnOP	84	0·48 (0·17, 1·38)			55	**0·17 (0·03, 0·81)**	58	0·86 (0·28, 2·62)
DiDP	136	1·34 (0·66, 2·7)			98	0·98 (0·4, 2·35)	89	0·78 (0·34, 1·81)

Outcome: Low Birthweight (Terms only)

	Across Pregnancy		Trimester 1		Trimester 2		Trimester 3	
**Exposure**	**N**	**OR (95% CI)**	**N**	**OR (95% CI)**	**N**	**OR (95% CI)**	**N**	**OR (95% CI)**
LMW	2005	0·96 (0·54, 1·7)	470	0·87 (0·37, 2·06)	1441	0·81 (0·41, 1·57)	1427	0·88 (0·5, 1·56)
HMW	2057	0·92 (0·46, 1·85)	568	1·94 (0·64, 5·86)	1424	0·88 (0·41, 1·9)	1298	0·67 (0·31, 1·43)
DEHP	2059	1·03 (0·54, 1·97)	576	2·18 (0·77, 6·18)	1426	1·02 (0·49, 2·11)	1303	0·82 (0·41, 1·65)
PA	1196	1·51 (0·7, 3·27)	435	2·52 (0·85, 7·47)	976	1·31 (0·61, 2·79)	987	0·58 (0·3, 1·11)
DiNP	1301	1·04 (0·58, 1·87)	398	0·69 (0·28, 1·73)	1088	0·78 (0·42, 1·42)	625	0·92 (0·51, 1·67)
DnOP	922	0·95 (0·36, 2·5)	390	1·29 (0·5, 3·28)	791	0·61 (0·24, 1·55)	541	0·89 (0·38, 2·06)
DiDP	1727	1·09 (0·59, 1·99)	441	1·5 (0·69, 3·24)	1380	0·69 (0·38, 1·23)	1178	1·29 (0·75, 2·22)

Supplement Table 4a1: Race/Ethnicity-Stratified Adjusted Associations of Phthalate Exposures with Birth Outcomes (Continuously Measured), Non-Hispanic White Only

Models adjusted for maternal parity, and education, and child sex· BW for GA model removes parity from covariates·

Outcome: Gestational Age

	Across Pregnancy		Trimester 1		Trimester 2		Trimester 3	
**Exposure**	**N**	**Beta (95% CI)**	**N**	**Beta (95% CI)**	**N**	**Beta (95% CI)**	**N**	**Beta (95% CI)**
LMW	1794	**-0·275 (-0·457, -0·092)**	483	-0·19 (-0·435, 0·056)	1354	0·023 (-0·139, 0·186)	1169	**-0·334 (-0·533, -0·134)**
HMW	1821	**-0·477 (-0·708, -0·246)**	599	**-0·408 (-0·69, -0·126)**	1311	0·068 (-0·123, 0·259)	1031	**-0·506 (-0·81, -0·202)**
DEHP	1822	**-0·425 (-0·647, -0·203)**	599	**-0·369 (-0·652, -0·086)**	1314	0·039 (-0·143, 0·222)	1033	**-0·501 (-0·791, -0·21)**
PA	1154	**-0·899 (-1·125, -0·673)**	470	-0·116 (-0·349, 0·116)	864	**-0·21 (-0·379, -0·041)**	964	**-0·645 (-0·837, -0·453)**
DiNP	1432	**-0·573 (-0·742, -0·404)**	419	-0·007 (-0·238, 0·224)	1155	-0·017 (-0·158, 0·123)	751	**-0·776 (-0·993, -0·558)**
DnOP	913	**-0·954 (-1·294, -0·615)**	409	-0·083 (-0·351, 0·184)	720	0·038 (-0·202, 0·279)	657	**-0·833 (-1·154, -0·511)**
DiDP	1733	**-0·32 (-0·497, -0·144)**	476	-0·002 (-0·223, 0·219)	1311	0·092 (-0·058, 0·242)	1116	**-0·391 (-0·581, -0·2)**

Outcome: Birth Length

	Across Pregnancy		Trimester 1		Trimester 2		Trimester 3	
**Exposure**	**N**	**Beta (95% CI)**	**N**	**Beta (95% CI)**	**N**	**Beta (95% CI)**	**N**	**Beta (95% CI)**
LMW	1423	**-0·511 (-0·884, -0·139)**	347	**-0·624 (-1·184, -0·065)**	1143	0·051 (-0·275, 0·377)	920	**-0·398 (-0·794, -0·003)**
HMW	1503	**-0·669 (-1·115, -0·224)**	488	0·129 (-0·462, 0·721)	1115	0·2 (-0·174, 0·573)	797	**-0·689 (-1·292, -0·087)**
DEHP	1504	**-0·608 (-1·039, -0·178)**	488	-0·079 (-0·67, 0·512)	1118	0·204 (-0·15, 0·558)	799	**-0·657 (-1·233, -0·082)**
PA	807	**-1·411 (-1·944, -0·878)**	335	-0·279 (-0·786, 0·229)	669	0·196 (-0·177, 0·568)	731	**-1·074 (-1·509, -0·639)**
DiNP	1187	**-1·175 (-1·502, -0·849)**	347	0·075 (-0·42, 0·57)	984	-0·066 (-0·353, 0·221)	537	**-1·552 (-1·996, -1·109)**
DnOP	670	**-1·33 (-2·07, -0·59)**	335	0·038 (-0·523, 0·599)	552	0·373 (-0·157, 0·902)	444	**-1·538 (-2·244, -0·833)**
DiDP	1396	**-1·011 (-1·382, -0·639)**	347	-0·018 (-0·497, 0·461)	1116	-0·005 (-0·313, 0·303)	886	**-1·225 (-1·59, -0·86)**

Outcome: Birthweight

	Across Pregnancy		Trimester 1		Trimester 2		Trimester 3	
**Exposure**	**N**	**Beta (95% CI)**	**N**	**Beta (95% CI)**	**N**	**Beta (95% CI)**	**N**	**Beta (95% CI)**
LMW	1794	**-76·495 (-132·726, -20·264)**	483	-8·549 (-96·282, 79·185)	1354	3·213 (-51·781, 58·207)	1169	**-67·766 (-127·895, -7·637)**
HMW	1821	-25·556 (-96·301, 45·189)	599	16·023 (-83·715, 115·76)	1311	44·028 (-20·932, 108·989)	1031	-85·146 (-175·885, 5·593)
DEHP	1822	-18·68 (-86·735, 49·374)	599	15·427 (-84·46, 115·314)	1314	33·548 (-28·401, 95·498)	1033	-78·198 (-164·938, 8·543)
PA	1154	**-164·163 (-233·982, -94·345)**	470	-27·616 (-110·737, 55·506)	864	-7·531 (-67·126, 52·064)	964	**-125·167 (-183·599, -66·734)**
DiNP	1432	**-123·87 (-175·625, -72·116)**	419	41·074 (-41·412, 123·56)	1155	14·993 (-32·977, 62·963)	751	**-161·617 (-225·241, -97·993)**
DnOP	913	**-196·136 (-297·125, -95·148)**	409	-53·122 (-148·867, 42·622)	720	1·184 (-82·398, 84·766)	657	**-210·676 (-303·48, -117·873)**
DiDP	1733	**-55·582 (-109·891, -1·273)**	476	14·186 (-64·385, 92·756)	1311	36·923 (-14·312, 88·159)	1116	**-92·955 (-150·516, -35·394)**

Outcome: Birthweight for Gestational Age Z-Score

	Across Pregnancy		Trimester 1		Trimester 2		Trimester 3	
**Exposure**	**N**	**Beta (95% CI)**	**N**	**Beta (95% CI)**	**N**	**Beta (95% CI)**	**N**	**Beta (95% CI)**
LMW	1648	**-0·119 (-0·234, -0·004)**	361	0·004 (-0·219, 0·227)	1219	-0·065 (-0·191, 0·06)	1030	-0·09 (-0·211, 0·031)
HMW	1674	0·137 (-0·005, 0·279)	476	0·069 (-0·176, 0·313)	1176	0·095 (-0·049, 0·239)	892	0·042 (-0·134, 0·217)
DEHP	1675	**0·138 (0·001, 0·276)**	476	0·069 (-0·184, 0·321)	1179	0·099 (-0·039, 0·237)	894	0·037 (-0·131, 0·206)
PA	1010	0·015 (-0·121, 0·15)	349	-0·016 (-0·229, 0·198)	731	0·084 (-0·048, 0·216)	826	-0·019 (-0·131, 0·092)
DiNP	1286	-0·007 (-0·113, 0·098)	297	0·1 (-0·114, 0·314)	1020	0·069 (-0·04, 0·178)	612	0·008 (-0·115, 0·131)
DnOP	769	-0·039 (-0·233, 0·155)	288	-0·143 (-0·398, 0·112)	587	-0·026 (-0·212, 0·159)	519	-0·127 (-0·312, 0·059)
DiDP	1587	-0·009 (-0·122, 0·105)	354	-0·033 (-0·227, 0·162)	1176	0·014 (-0·108, 0·136)	977	-0·06 (-0·173, 0·054)

Supplement Table 4b1: Race/Ethnicity -Stratified Adjusted Associations of Phthalate Exposures with Categorical Birth Outcomes, Non-Hispanic White Only

Models adjusted for maternal parity, and education, and child sex·

Outcome: Preterm

	Across Pregnancy		Trimester 1		Trimester 2		Trimester 3	
**Exposure**	**N**	**OR (95% CI)**	**N**	**OR (95% CI)**	**N**	**OR (95% CI)**	**N**	**OR (95% CI)**
LMW	1794	1·06 (0·69, 1·63)	483	1·63 (0·55, 4·88)	1354	0·65 (0·37, 1·14)	1169	1·38 (0·91, 2·09)
HMW	1821	1·65 (0·97, 2·79)	599	1·96 (0·57, 6·75)	1311	0·61 (0·32, 1·17)	1031	**1·89 (1·04, 3·45)**
DEHP	1822	1·4 (0·84, 2·32)	599	1·74 (0·5, 6·05)	1314	0·63 (0·34, 1·18)	1033	1·76 (1, 3·09)
PA	1154	**3·91 (2·45, 6·24)**	470	2·45 (0·82, 7·39)	864	1·13 (0·61, 2·08)	964	**2·95 (1·91, 4·58)**
DiNP	1432	**3·15 (2·2, 4·49)**	419	0·84 (0·28, 2·55)	1155	1·01 (0·64, 1·6)	751	**3·23 (2·2, 4·75)**
DnOP	913	**4·26 (2·33, 7·77)**	409	0·53 (0·13, 2·14)	720	0·78 (0·32, 1·87)	657	**3·01 (1·83, 4·94)**
DiDP	1733	**1·89 (1·25, 2·85)**	476	0·71 (0·25, 1·99)	1311	**0·53 (0·33, 0·85)**	1116	**2·65 (1·77, 3·95)**

Outcome: Small for Gestational Age

	Across Pregnancy		Trimester 1		Trimester 2		Trimester 3	
**Exposure**	**N**	**OR (95% CI)**	**N**	**OR (95% CI)**	**N**	**OR (95% CI)**	**N**	**OR (95% CI)**
LMW	1794	1·08 (0·74, 1·59)	483	1·1 (0·61, 1·99)	1354	1·11 (0·73, 1·67)	1169	0·89 (0·59, 1·34)
HMW	1820	0·94 (0·58, 1·54)	598	1·08 (0·54, 2·16)	1311	0·93 (0·56, 1·55)	1031	1·44 (0·81, 2·55)
DEHP	1821	0·91 (0·56, 1·46)	598	1·04 (0·51, 2·1)	1314	0·93 (0·57, 1·51)	1033	1·14 (0·65, 2)
PA	1154	1·03 (0·66, 1·61)	470	1·06 (0·59, 1·88)	864	1 (0·64, 1·56)	964	1·27 (0·85, 1·89)
DiNP	1432	1·33 (0·94, 1·89)	419	0·8 (0·43, 1·47)	1155	1·04 (0·71, 1·52)	751	1·19 (0·82, 1·75)
DnOP	913	1·44 (0·77, 2·72)	409	0·87 (0·42, 1·79)	720	1·56 (0·86, 2·84)	657	1·58 (0·94, 2·66)
DiDP	1733	1·11 (0·77, 1·61)	476	0·94 (0·54, 1·63)	1311	1·06 (0·72, 1·58)	1116	1·34 (0·92, 1·95)

Outcome: Large for Gestational Age

	Across Pregnancy		Trimester 1		Trimester 2		Trimester 3	
**Exposure**	**N**	**OR (95% CI)**	**N**	**OR (95% CI)**	**N**	**OR (95% CI)**	**N**	**OR (95% CI)**
LMW	1794	0·93 (0·68, 1·29)	483	1·28 (0·76, 2·16)	1354	0·99 (0·71, 1·38)	1169	1·01 (0·72, 1·4)
HMW	1820	1·05 (0·7, 1·57)	598	1·45 (0·78, 2·67)	1311	0·83 (0·56, 1·24)	1031	0·9 (0·55, 1·47)
DEHP	1821	1·06 (0·72, 1·56)	598	1·52 (0·84, 2·76)	1314	0·83 (0·57, 1·22)	1033	0·92 (0·57, 1·47)
PA	1154	1·31 (0·91, 1·88)	470	0·99 (0·61, 1·63)	864	**1·62 (1·1, 2·38)**	964	1·07 (0·79, 1·44)
DiNP	1432	0·97 (0·73, 1·28)	419	1·15 (0·71, 1·87)	1155	1·08 (0·81, 1·45)	751	0·81 (0·59, 1·13)
DnOP	913	0·92 (0·55, 1·54)	409	0·59 (0·33, 1·06)	720	1 (0·6, 1·66)	657	0·78 (0·5, 1·21)
DiDP	1733	1·21 (0·89, 1·65)	476	1·44 (0·9, 2·29)	1311	1·17 (0·85, 1·59)	1116	1·08 (0·78, 1·48)

Outcome: Low Birthweight

	Across Pregnancy		Trimester 1		Trimester 2		Trimester 3	
**Exposure**	**N**	**OR (95% CI)**	**N**	**OR (95% CI)**	**N**	**OR (95% CI)**	**N**	**OR (95% CI)**
LMW	1794	**1·78 (1·12, 2·85)**	483	1·14 (0·36, 3·61)	1354	0·92 (0·46, 1·85)	1169	**1·58 (1·02, 2·44)**
HMW	1821	1·73 (0·93, 3·2)	599	2·45 (0·74, 8·12)	1311	0·74 (0·32, 1·72)	1031	1·75 (0·93, 3·28)
DEHP	1822	1·61 (0·9, 2·89)	599	2·88 (0·91, 9·18)	1314	0·9 (0·41, 2·01)	1033	1·6 (0·88, 2·9)
PA	1154	**4·01 (2·42, 6·64)**	470	1·68 (0·52, 5·42)	864	1·04 (0·54, 2·01)	964	**2·97 (1·86, 4·76)**
DiNP	1432	**3·26 (2·14, 4·98)**	419	0·51 (0·13, 1·99)	1155	0·91 (0·48, 1·71)	751	**2·79 (1·86, 4·18)**
DnOP	913	**3·84 (2, 7·38)**	409	1·09 (0·26, 4·55)	720	0·79 (0·3, 2·08)	657	**2·76 (1·62, 4·69)**
DiDP	1733	**1·66 (1·01, 2·73)**	476	1·46 (0·56, 3·76)	1311	0·56 (0·3, 1·05)	1116	**2·08 (1·36, 3·21)**

Outcome: Low Birthweight (Preterms only)

	Across Pregnancy		Trimester 1		Trimester 2		Trimester 3	
Exposure	N	OR (95% CI)	N	OR (95% CI)	N	OR (95% CI)	N	OR (95% CI)
LMW	116	2·17 (0·98, 4·81)	16	0·4 (0·02, 6·5)	68	0·83 (0·23, 2·93)	73	1·16 (0·51, 2·64)
HMW	115	1·79 (0·54, 5·87)			66	1·4 (0·34, 5·73)	71	0·8 (0·2, 3·21)
DEHP	115	1·76 (0·58, 5·33)			66	1·75 (0·46, 6·67)	71	0·99 (0·26, 3·8)
PA	81	1·82 (0·83, 4)	16	0·14 (0, 4·98)	36	0·46 (0·09, 2·47)	66	1·82 (0·83, 4·03)
DiNP	99	1·93 (0·98, 3·79)			57	0·79 (0·24, 2·6)	65	1·19 (0·62, 2·29)
DnOP	69	1·13 (0·38, 3·31)			30	0·19 (0·02, 2·02)	61	1·4 (0·52, 3·79)
DiDP	114	1·27 (0·64, 2·52)	15	0·07 (0, 31·34)	65	0·92 (0·25, 3·47)	72	0·63 (0·28, 1·4)

Outcome: Low Birthweight (Terms only)

	Across Pregnancy		Trimester 1		Trimester 2		Trimester 3	
Exposure	N	OR (95% CI)	N	OR (95% CI)	N	OR (95% CI)	N	OR (95% CI)
LMW	1678	1·7 (0·77, 3·76)	467	1·39 (0·38, 5·14)	1286	1·18 (0·42, 3·32)	1096	1·52 (0·71, 3·25)
HMW	1706	1·25 (0·45, 3·47)	582	2·73 (0·74, 10·04)	1245	0·73 (0·19, 2·89)	960	1·7 (0·59, 4·84)
DEHP	1707	1·32 (0·5, 3·47)	582	3·19 (0·92, 11·04)	1248	0·97 (0·27, 3·56)	962	1·41 (0·51, 3·9)
PA	1073	1·76 (0·64, 4·82)	454	1·5 (0·37, 6·02)	828	1·29 (0·45, 3·69)	898	1·28 (0·55, 3)
DiNP	1333	1·91 (0·9, 4·06)	408	0·54 (0·11, 2·67)	1098	1·37 (0·57, 3·31)	686	1·63 (0·81, 3·28)
DnOP	844	1·73 (0·45, 6·67)	398	1·41 (0·27, 7·23)	690	1·54 (0·42, 5·67)	596	1·31 (0·47, 3·68)
DiDP	1619	1·15 (0·47, 2·77)	461	1·57 (0·5, 4·9)	1246	0·84 (0·3, 2·36)	1044	1·85 (0·88, 3·87)

Supplement Table 4a2: Race/Ethnicity-Stratified Adjusted Associations of Phthalate Exposures with Birth Outcomes (Continuously Measured), Non-Hispanic Black Only

Models adjusted for maternal parity, and education, and child sex· BW for GA model removes parity from covariates·

Outcome: Gestational Age

	Across Pregnancy		Trimester 1		Trimester 2		Trimester 3	
**Exposure**	**N**	**Beta (95% CI)**	**N**	**Beta (95% CI)**	**N**	**Beta (95% CI)**	**N**	**Beta (95% CI)**
LMW	1115	0·209 (-0·077, 0·494)	134	0·252 (-0·46, 0·965)	803	0·202 (-0·136, 0·54)	919	0·194 (-0·036, 0·424)
HMW	1143	-0·036 (-0·36, 0·287)	153	0·387 (-0·428, 1·203)	794	0·319 (-0·037, 0·675)	827	-0·058 (-0·352, 0·236)
DEHP	1150	-0·058 (-0·356, 0·24)	182	0·178 (-0·525, 0·881)	798	0·27 (-0·059, 0·6)	838	-0·11 (-0·369, 0·149)
PA	814	-0·15 (-0·591, 0·29)	64	0·552 (-0·494, 1·598)	690	-0·08 (-0·499, 0·338)	712	0·091 (-0·249, 0·432)
DiNP	669	**-0·451 (-0·815, -0·087)**	53	0·423 (-0·488, 1·333)	556	0·023 (-0·315, 0·361)	164	-0·044 (-0·582, 0·494)
DnOP	545	-0·533 (-1·181, 0·115)	52	0·33 (-0·583, 1·243)	459	0·009 (-0·6, 0·618)	152	-0·159 (-0·836, 0·518)
DiDP	881	**-0·668 (-1·039, -0·298)**	62	0·509 (-0·424, 1·443)	721	-0·027 (-0·392, 0·338)	754	-0·179 (-0·426, 0·069)

Outcome: Birth Length

	Across Pregnancy		Trimester 1		Trimester 2		Trimester 3	
**Exposure**	**N**	**Beta (95% CI)**	**N**	**Beta (95% CI)**	**N**	**Beta (95% CI)**	**N**	**Beta (95% CI)**
LMW	1024	0·362 (-0·098, 0·822)	82	-0·329 (-1·572, 0·915)	740	0·212 (-0·32, 0·745)	874	**0·431 (0·047, 0·815)**
HMW	1056	0·497 (-0·026, 1·019)	106	0·301 (-1·234, 1·836)	734	**0·613 (0·045, 1·181)**	787	0·46 (-0·028, 0·947)
DEHP	1063	**0·489 (0·007, 0·972)**	131	0·292 (-1·025, 1·609)	738	**0·607 (0·083, 1·132)**	798	0·404 (-0·028, 0·836)
PA	756	-0·064 (-0·779, 0·651)	39	1·053 (-0·24, 2·345)	652	-0·029 (-0·685, 0·626)	676	0·035 (-0·539, 0·608)
DiNP	633	-0·561 (-1·125, 0·003)	40	0·302 (-0·9, 1·504)	527	-0·013 (-0·526, 0·501)	143	0·273 (-0·657, 1·203)
DnOP	513	-0·509 (-1·495, 0·476)	39	0·36 (-0·907, 1·627)	434	0·152 (-0·758, 1·063)	130	0·392 (-0·682, 1·465)
DiDP	827	**-0·998 (-1·596, -0·4)**	40	-0·163 (-1·374, 1·048)	684	0·15 (-0·423, 0·723)	718	-0·344 (-0·762, 0·074)

Outcome: Birthweight

	Across Pregnancy		Trimester 1		Trimester 2		Trimester 3	
**Exposure**	**N**	**Beta (95% CI)**	**N**	**Beta (95% CI)**	**N**	**Beta (95% CI)**	**N**	**Beta (95% CI)**
LMW	1115	45·66 (-31·155, 122·476)	134	-53·713 (-242·193, 134·767)	803	9·272 (-79·937, 98·481)	919	**73·397 (4·353, 142·441)**
HMW	1143	25·018 (-62·433, 112·469)	153	32·441 (-194·687, 259·569)	794	30·961 (-63·021, 124·943)	827	59·286 (-28·008, 146·579)
DEHP	1150	1·261 (-79·308, 81·829)	182	1·848 (-195·549, 199·246)	798	27·166 (-59·727, 114·059)	838	16·566 (-60·404, 93·536)
PA	814	-64·509 (-182·818, 53·8)	64	102·334 (-168·574, 373·242)	690	-48·244 (-159·355, 62·868)	712	-18·254 (-122·369, 85·861)
DiNP	669	-86·746 (-182·217, 8·725)	53	152·773 (-92·954, 398·5)	556	2·455 (-84·64, 89·55)	164	39·294 (-107·697, 186·285)
DnOP	545	-121·53 (-285·735, 42·675)	52	131·548 (-107·794, 370·89)	459	13·165 (-140·623, 166·952)	152	-38·728 (-216·457, 139·002)
DiDP	881	**-156·965 (-257·612, -56·317)**	62	214·052 (-29·487, 457·591)	721	18·839 (-78·314, 115·992)	754	-72·469 (-148·418, 3·48)

Outcome: Birthweight for Gestational Age Z-Score

	Across Pregnancy		Trimester 1		Trimester 2		Trimester 3	
**Exposure**	**N**	**Beta (95% CI)**	**N**	**Beta (95% CI)**	**N**	**Beta (95% CI)**	**N**	**Beta (95% CI)**
LMW	1080	0·044 (-0·113, 0·202)	106	-0·408 (-0·841, 0·024)	772	-0·074 (-0·251, 0·102)	887	0·142 (-0·013, 0·296)
HMW	1108	0·096 (-0·083, 0·275)	125	0·028 (-0·478, 0·533)	763	-0·116 (-0·302, 0·069)	795	**0·216 (0·02, 0·413)**
DEHP	1115	0·071 (-0·095, 0·236)	154	0·043 (-0·421, 0·506)	767	-0·077 (-0·25, 0·096)	806	0·135 (-0·039, 0·308)
PA	783	-0·112 (-0·343, 0·119)	37	0·046 (-0·486, 0·579)	660	-0·141 (-0·356, 0·075)	683	-0·05 (-0·28, 0·179)
DiNP	638	0·002 (-0·177, 0·181)	26	0·342 (-0·242, 0·926)	526	-0·015 (-0·179, 0·15)	135	0·051 (-0·279, 0·381)
DnOP	514	-0·024 (-0·336, 0·288)	25	-0·04 (-0·705, 0·625)	429	-0·023 (-0·321, 0·275)	123	0·03 (-0·385, 0·444)
DiDP	850	-0·076 (-0·272, 0·119)	35	0·045 (-0·464, 0·554)	691	0·041 (-0·154, 0·237)	725	-0·131 (-0·295, 0·033)

Supplement Table 4b2: Race/Ethnicity -Stratified Adjusted Associations of Phthalate Exposures with Categorical Birth Outcomes, Non-Hispanic Black Only

Models adjusted for maternal parity, and education, and child sex·

Outcome: Preterm

	Across Pregnancy		Trimester 1		Trimester 2		Trimester 3	
**Exposure**	**N**	**OR (95% CI)**	**N**	**OR (95% CI)**	**N**	**OR (95% CI)**	**N**	**OR (95% CI)**
LMW	1115	0·68 (0·41, 1·1)	134	0·39 (0·11, 1·41)	803	**0·51 (0·28, 0·94)**	919	0·74 (0·45, 1·22)
HMW	1143	1·19 (0·69, 2·05)	153	0·45 (0·07, 2·96)	794	**0·51 (0·27, 0·94)**	827	1·57 (0·89, 2·78)
DEHP	1150	1·16 (0·71, 1·91)	182	0·46 (0·12, 1·81)	798	**0·54 (0·3, 0·96)**	838	1·54 (0·94, 2·53)
PA	814	1·19 (0·58, 2·46)	64	0·75 (0·05, 11·16)	690	0·79 (0·39, 1·6)	712	1·12 (0·52, 2·41)
DiNP	669	1·5 (0·86, 2·63)	53	2·6 (0·38, 17·82)	556	0·8 (0·47, 1·36)	164	0·99 (0·44, 2·25)
DnOP	545	**3·1 (1·35, 7·13)**	52	0·74 (0·12, 4·43)	459	1·23 (0·55, 2·77)	152	1·71 (0·72, 4·06)
DiDP	881	1·54 (0·82, 2·88)	62	0·18 (0·02, 1·23)	721	0·73 (0·4, 1·34)	754	1·09 (0·62, 1·91)

Outcome: Small for Gestational Age

	Across Pregnancy		Trimester 1		Trimester 2		Trimester 3	
**Exposure**	**N**	**OR (95% CI)**	**N**	**OR (95% CI)**	**N**	**OR (95% CI)**	**N**	**OR (95% CI)**
LMW	1111	**0·64 (0·42, 0·98)**	133	1·02 (0·39, 2·64)	802	**0·59 (0·36, 0·98)**	916	0·68 (0·46, 1·01)
HMW	1139	0·67 (0·42, 1·07)	152	0·64 (0·19, 2·23)	793	0·96 (0·59, 1·56)	824	**0·59 (0·35, 0·98)**
DEHP	1146	0·81 (0·53, 1·25)	181	0·7 (0·25, 1·97)	797	0·97 (0·61, 1·53)	835	0·75 (0·48, 1·17)
PA	814	0·78 (0·42, 1·44)	64	2·31 (0·57, 9·3)	690	0·8 (0·45, 1·41)	712	0·79 (0·44, 1·43)
DiNP	669	0·83 (0·51, 1·35)	53	1·68 (0·53, 5·3)	556	0·97 (0·63, 1·5)	164	0·79 (0·39, 1·62)
DnOP	545	0·76 (0·34, 1·69)	52	1·28 (0·43, 3·83)	459	0·72 (0·34, 1·5)	152	1·01 (0·43, 2·35)
DiDP	881	0·83 (0·49, 1·41)	62	0·95 (0·28, 3·17)	721	0·89 (0·53, 1·47)	754	0·95 (0·62, 1·45)

Outcome: Large for Gestational Age

	Across Pregnancy		Trimester 1		Trimester 2		Trimester 3	
**Exposure**	**N**	**OR (95% CI)**	**N**	**OR (95% CI)**	**N**	**OR (95% CI)**	**N**	**OR (95% CI)**
LMW	1111	1·08 (0·59, 1·99)	133	0·19 (0·02, 1·46)	802	0·61 (0·28, 1·33)	916	1·38 (0·78, 2·45)
HMW	1139	1·29 (0·65, 2·56)	152	0·81 (0·1, 6·83)	793	0·67 (0·31, 1·44)	824	1·3 (0·65, 2·63)
DEHP	1146	1·13 (0·6, 2·14)	181	0·68 (0·12, 4)	797	0·65 (0·32, 1·34)	835	1·07 (0·56, 2·04)
PA	814	0·99 (0·4, 2·48)	64	5·85 (0·21, 166·73)	690	0·87 (0·36, 2·08)	712	0·85 (0·35, 2·08)
DiNP	669	1·18 (0·54, 2·61)	53	6·08 (0·81, 45·94)	556	1·01 (0·5, 2·07)	164	0·67 (0·19, 2·35)
DnOP	545	0·85 (0·21, 3·43)	52	2·15 (0·3, 15·57)	459	0·69 (0·18, 2·56)	152	0·7 (0·15, 3·25)
DiDP	881	1·31 (0·57, 3)	62	**857·59 (1·98, 371026·27)**	721	1·33 (0·62, 2·84)	754	0·86 (0·43, 1·73)

Outcome: Low Birthweight

	Across Pregnancy		Trimester 1		Trimester 2		Trimester 3	
**Exposure**	**N**	**OR (95% CI)**	**N**	**OR (95% CI)**	**N**	**OR (95% CI)**	**N**	**OR (95% CI)**
LMW	1115	0·66 (0·39, 1·11)	134	0·88 (0·27, 2·9)	803	0·85 (0·47, 1·54)	919	**0·55 (0·31, 0·97)**
HMW	1143	0·99 (0·55, 1·78)	153	0·4 (0·06, 2·79)	794	0·78 (0·42, 1·45)	827	0·85 (0·43, 1·68)
DEHP	1150	1·11 (0·65, 1·89)	182	0·37 (0·08, 1·72)	798	0·78 (0·44, 1·38)	838	1·18 (0·67, 2·09)
PA	814	1·17 (0·56, 2·46)	64	0·94 (0·22, 4·02)	690	1·33 (0·66, 2·68)	712	0·7 (0·31, 1·58)
DiNP	669	1·59 (0·91, 2·77)	53	0·23 (0·02, 2·2)	556	0·87 (0·52, 1·46)	164	0·76 (0·34, 1·69)
DnOP	545	1·52 (0·65, 3·57)	52	0·32 (0·05, 1·84)	459	0·71 (0·3, 1·66)	152	1·51 (0·61, 3·75)
DiDP	881	**2·54 (1·37, 4·71)**	62	0·29 (0·06, 1·43)	721	0·74 (0·41, 1·36)	754	1·64 (0·93, 2·89)

Outcome: Low Birthweight (Preterms only)

	Across Pregnancy		Trimester 1		Trimester 2		Trimester 3	
**Exposure**	**N**	**OR (95% CI)**	**N**	**OR (95% CI)**	**N**	**OR (95% CI)**	**N**	**OR (95% CI)**
LMW	117	0·78 (0·32, 1·9)	18	1·3 (0, 599·5)	79	3·02 (0·77, 11·81)	84	0·54 (0·21, 1·41)
HMW	115	1·34 (0·49, 3·62)			78	1·59 (0·43, 5·93)	76	1·17 (0·42, 3·28)
DEHP	117	1·27 (0·49, 3·31)	20	0·01 (0, 51·53)	79	1·15 (0·34, 3·89)	77	1·46 (0·56, 3·85)
PA	83	1·18 (0·3, 4·63)			65	3·12 (0·61, 15·87)	61	0·96 (0·24, 3·89)
DiNP	70	3 (0·96, 9·35)			57	1·77 (0·51, 6·09)	24	2·22 (0·42, 11·84)
DnOP	63	0·71 (0·16, 3·2)			50	0·65 (0·15, 2·81)	25	1·16 (0·17, 7·95)
DiDP	86	**4·37 (1·43, 13·35)**			68	1·62 (0·54, 4·84)	63	2·92 (0·94, 9·05)

Outcome: Low Birthweight (Terms only)

	Across Pregnancy		Trimester 1		Trimester 2		Trimester 3	
**Exposure**	**N**	**OR (95% CI)**	**N**	**OR (95% CI)**	**N**	**OR (95% CI)**	**N**	**OR (95% CI)**
LMW	998	0·68 (0·3, 1·52)	116	1·76 (0·37, 8·43)	724	0·77 (0·3, 1·97)	835	0·61 (0·28, 1·35)
HMW	1028	0·61 (0·24, 1·56)	140	0·74 (0·07, 7·92)	716	0·88 (0·33, 2·32)	751	0·36 (0·12, 1·09)
DEHP	1033	0·9 (0·39, 2·08)	162	1·3 (0·22, 7·75)	719	1·06 (0·44, 2·58)	761	0·72 (0·3, 1·73)
PA	731	1·03 (0·33, 3·25)	57	1·57 (0·25, 9·77)	625	1·52 (0·52, 4·45)	651	0·44 (0·14, 1·39)
DiNP	599	0·83 (0·33, 2·08)	47	0·21 (0·01, 4·05)	499	0·69 (0·3, 1·58)	140	0·36 (0·1, 1·21)
DnOP	482	0·73 (0·16, 3·29)	46	0·18 (0·01, 2·54)	409	0·49 (0·12, 1·97)	127	1·1 (0·24, 4·91)
DiDP	795	1·08 (0·39, 3)	55	0·41 (0·05, 3·07)	653	0·42 (0·17, 1·05)	691	1·26 (0·56, 2·82)

Supplement Table 4a3: Race/Ethnicity-Stratified Adjusted Associations of Phthalate Exposures with Birth Outcomes (Continuously Measured), Hispanic Only

Models adjusted for maternal parity, and education, and child sex· BW for GA model removes parity from covariates·

Outcome: Gestational Age

	Across Pregnancy		Trimester 1		Trimester 2		Trimester 3	
Exposure	N	Beta (95% CI)	N	Beta (95% CI)	N	Beta (95% CI)	N	Beta (95% CI)
LMW	1156	0·174 (-0·038, 0·385)	276	-0·095 (-0·493, 0·304)	733	0·012 (-0·268, 0·293)	784	0·164 (-0·047, 0·375)
HMW	1154	0·18 (-0·076, 0·435)	296	-0·246 (-0·742, 0·249)	726	0·078 (-0·298, 0·453)	767	0·088 (-0·177, 0·353)
DEHP	1154	0·131 (-0·112, 0·375)	296	-0·186 (-0·665, 0·292)	726	0·103 (-0·267, 0·472)	767	0·094 (-0·156, 0·345)
PA	418	-0·021 (-0·474, 0·433)	273	0·142 (-0·295, 0·58)	363	0·267 (-0·156, 0·69)	296	-0·225 (-0·593, 0·144)
DiNP	487	-0·158 (-0·441, 0·126)	264	0·055 (-0·313, 0·422)	400	-0·033 (-0·351, 0·284)	336	-0·111 (-0·382, 0·159)
DnOP	392	-0·318 (-0·809, 0·172)	262	0·145 (-0·271, 0·562)	352	-0·144 (-0·589, 0·301)	282	-0·43 (-0·869, 0·008)
DiDP	859	0·147 (-0·132, 0·426)	275	0·11 (-0·219, 0·438)	717	-0·041 (-0·295, 0·214)	494	-0·069 (-0·321, 0·184)

Outcome: Birth Length

	Across Pregnancy		Trimester 1		Trimester 2		Trimester 3	
Exposure	N	Beta (95% CI)	N	Beta (95% CI)	N	Beta (95% CI)	N	Beta (95% CI)
LMW	1012	0·182 (-0·195, 0·558)	241	-0·201 (-0·833, 0·431)	666	0·261 (-0·22, 0·743)	678	0·002 (-0·396, 0·4)
HMW	1018	-0·032 (-0·486, 0·422)	263	-0·128 (-0·953, 0·696)	661	-0·121 (-0·756, 0·514)	665	-0·044 (-0·543, 0·455)
DEHP	1018	0·083 (-0·351, 0·517)	263	0·285 (-0·497, 1·068)	661	0·053 (-0·573, 0·679)	665	0·121 (-0·354, 0·597)
PA	328	0·727 (-0·126, 1·581)	238	0·339 (-0·372, 1·05)	312	0·354 (-0·352, 1·061)	231	0·418 (-0·24, 1·075)
DiNP	418	**-0·59 (-1·049, -0·13)**	240	0·143 (-0·459, 0·746)	356	-0·315 (-0·83, 0·201)	275	**-0·475 (-0·93, -0·02)**
DnOP	322	-0·352 (-1·146, 0·443)	238	0·179 (-0·494, 0·853)	308	-0·39 (-1·094, 0·315)	220	-0·302 (-1·043, 0·439)
DiDP	758	0·09 (-0·401, 0·581)	241	0·316 (-0·202, 0·835)	656	-0·035 (-0·464, 0·395)	426	-0·182 (-0·644, 0·281)

Outcome: Birthweight

	Across Pregnancy		Trimester 1		Trimester 2		Trimester 3	
**Exposure**	**N**	**Beta (95% CI)**	**N**	**Beta (95% CI)**	**N**	**Beta (95% CI)**	**N**	**Beta (95% CI)**
LMW	1156	43·961 (-17·811, 105·733)	276	-31·865 (-145·554, 81·823)	733	25·907 (-58·409, 110·222)	784	20·077 (-43·495, 83·649)
HMW	1154	35·32 (-39·331, 109·97)	296	65·168 (-74·503, 204·84)	726	-36·803 (-149·803, 76·197)	767	28·251 (-51·565, 108·067)
DEHP	1154	42·237 (-28·8, 113·274)	296	79·646 (-55·118, 214·409)	726	-6·275 (-117·56, 105·01)	767	39·827 (-35·597, 115·251)
PA	418	71·734 (-57·941, 201·409)	273	44·461 (-80·107, 169·028)	363	54·531 (-68·866, 177·928)	296	36·461 (-73·443, 146·365)
DiNP	487	8·14 (-72·692, 88·972)	264	85·437 (-18·15, 189·025)	400	38·089 (-54·483, 130·661)	336	-26·34 (-105·909, 53·23)
DnOP	392	-51·801 (-189·062, 85·46)	262	62·177 (-55·46, 179·815)	352	-32·704 (-159·381, 93·973)	282	-57·488 (-187·566, 72·59)
DiDP	859	21·867 (-60·424, 104·157)	275	34·646 (-59·182, 128·474)	717	-6·78 (-82·399, 68·84)	494	-13·432 (-91·998, 65·134)

Outcome: Birthweight for Gestational Age Z-Score

	Across Pregnancy		Trimester 1		Trimester 2		Trimester 3	
**Exposure**	**N**	**Beta (95% CI)**	**N**	**Beta (95% CI)**	**N**	**Beta (95% CI)**	**N**	**Beta (95% CI)**
LMW	1127	0·072 (-0·061, 0·205)	252	-0·032 (-0·259, 0·196)	710	0·119 (-0·058, 0·296)	758	0·02 (-0·121, 0·162)
HMW	1125	0·042 (-0·115, 0·2)	272	**0·298 (0·018, 0·579)**	703	-0·114 (-0·349, 0·121)	741	0·077 (-0·096, 0·25)
DEHP	1125	0·065 (-0·086, 0·216)	272	**0·28 (0·01, 0·55)**	703	-0·076 (-0·309, 0·157)	741	0·101 (-0·064, 0·265)
PA	393	0·232 (-0·012, 0·477)	249	0·088 (-0·158, 0·335)	341	0·033 (-0·208, 0·275)	273	**0·301 (0·079, 0·523)**
DiNP	462	0·079 (-0·076, 0·235)	240	**0·229 (0·019, 0·439)**	378	0·084 (-0·096, 0·263)	313	0·001 (-0·165, 0·167)
DnOP	367	0·036 (-0·225, 0·298)	238	0·101 (-0·147, 0·35)	330	0·004 (-0·243, 0·252)	259	0·058 (-0·212, 0·329)
DiDP	833	-0·035 (-0·208, 0·137)	251	0·029 (-0·159, 0·217)	694	0·02 (-0·139, 0·179)	471	-0·008 (-0·172, 0·156)

Supplement Table 4b3: Race/Ethnicity-Stratified Adjusted Associations of Phthalate Exposures with Categorical Birth Outcomes, Hispanic Only

Models adjusted for maternal parity, and education, and child sex·

Outcome: Preterm

	Across Pregnancy		Trimester 1		Trimester 2		Trimester 3	
**Exposure**	**N**	**OR (95% CI)**	**N**	**OR (95% CI)**	**N**	**OR (95% CI)**	**N**	**OR (95% CI)**
LMW	1156	0·96 (0·59, 1·58)	276	1·6 (0·63, 4·07)	733	1·45 (0·8, 2·61)	784	0·79 (0·45, 1·38)
HMW	1154	1·3 (0·73, 2·3)	296	1·6 (0·59, 4·32)	726	1·23 (0·55, 2·78)	767	1·71 (0·89, 3·26)
DEHP	1154	1·16 (0·66, 2·01)	296	1·28 (0·46, 3·51)	726	1·02 (0·45, 2·33)	767	1·38 (0·74, 2·56)
PA	418	1·39 (0·61, 3·17)	273	0·66 (0·28, 1·58)	363	1·2 (0·51, 2·85)	296	1·62 (0·66, 3·96)
DiNP	487	1·29 (0·73, 2·27)	264	1·08 (0·49, 2·42)	400	1·01 (0·51, 2·01)	336	1·47 (0·78, 2·78)
DnOP	392	**2·6 (1·14, 5·98)**	262	1·18 (0·5, 2·81)	352	1·83 (0·82, 4·11)	282	**4·87 (1·83, 12·98)**
DiDP	859	1·06 (0·57, 1·95)	275	0·91 (0·44, 1·91)	717	1·61 (0·92, 2·83)	494	1·2 (0·62, 2·34)

Outcome: Small for Gestational Age

	Across Pregnancy		Trimester 1		Trimester 2		Trimester 3	
**Exposure**	**N**	**OR (95% CI)**	**N**	**OR (95% CI)**	**N**	**OR (95% CI)**	**N**	**OR (95% CI)**
LMW	1153	0·81 (0·54, 1·22)	276	0·96 (0·51, 1·79)	733	0·78 (0·46, 1·3)	781	0·73 (0·48, 1·13)
HMW	1151	0·96 (0·59, 1·56)	296	0·43 (0·18, 1·02)	726	1·75 (0·93, 3·29)	764	0·94 (0·55, 1·59)
DEHP	1151	0·85 (0·53, 1·36)	296	0·49 (0·22, 1·11)	726	1·36 (0·72, 2·58)	764	0·84 (0·51, 1·41)
PA	418	0·79 (0·39, 1·63)	273	1·08 (0·53, 2·22)	363	1·15 (0·59, 2·25)	296	0·53 (0·27, 1·03)
DiNP	487	0·96 (0·59, 1·56)	264	0·52 (0·27, 1)	400	0·78 (0·45, 1·37)	336	1·27 (0·77, 2·09)
DnOP	392	1·2 (0·55, 2·61)	262	0·89 (0·44, 1·78)	352	1·05 (0·52, 2·12)	282	1·09 (0·48, 2·43)
DiDP	859	1·33 (0·81, 2·17)	275	1·41 (0·82, 2·42)	717	1·05 (0·67, 1·65)	494	1·11 (0·69, 1·76)

Outcome: Large for Gestational Age

	Across Pregnancy		Trimester 1		Trimester 2		Trimester 3	
**Exposure**	**N**	**OR (95% CI)**	**N**	**OR (95% CI)**	**N**	**OR (95% CI)**	**N**	**OR (95% CI)**
LMW	1153	1·22 (0·81, 1·84)	276	1·5 (0·57, 3·9)	733	1·27 (0·73, 2·19)	781	1·21 (0·76, 1·92)
HMW	1151	1·13 (0·69, 1·84)	296	1·98 (0·75, 5·25)	726	1·16 (0·56, 2·41)	764	1·14 (0·65, 2·02)
DEHP	1151	1·17 (0·74, 1·86)	296	2·14 (0·84, 5·44)	726	1·28 (0·63, 2·61)	764	1·13 (0·66, 1·93)
PA	418	1·88 (0·75, 4·73)	273	2·33 (0·78, 6·96)	363	2·17 (0·9, 5·24)	296	1·34 (0·48, 3·73)
DiNP	487	1·14 (0·63, 2·04)	264	2·04 (0·97, 4·29)	400	1·19 (0·63, 2·25)	336	0·99 (0·47, 2·1)
DnOP	392	1·35 (0·52, 3·54)	262	1·98 (0·83, 4·73)	352	1·02 (0·41, 2·56)	282	1·89 (0·59, 6)
DiDP	859	0·76 (0·42, 1·38)	275	1·07 (0·49, 2·32)	717	0·84 (0·5, 1·41)	494	0·87 (0·44, 1·7)

Outcome: Low Birthweight

	Across Pregnancy		Trimester 1		Trimester 2		Trimester 3	
**Exposure**	**N**	**OR (95% CI)**	**N**	**OR (95% CI)**	**N**	**OR (95% CI)**	**N**	**OR (95% CI)**
LMW	1156	0·98 (0·55, 1·73)	276	1·8 (0·68, 4·76)	733	1·05 (0·55, 2·01)	784	0·73 (0·39, 1·37)
HMW	1154	1·28 (0·65, 2·49)	296	1·94 (0·77, 4·87)	726	1·3 (0·56, 3·03)	767	1·02 (0·47, 2·2)
DEHP	1154	1·07 (0·56, 2·06)	296	1·77 (0·73, 4·3)	726	1·04 (0·44, 2·45)	767	0·96 (0·46, 2·01)
PA	418	0·87 (0·35, 2·21)	273	1·18 (0·45, 3·12)	363	0·93 (0·37, 2·34)	296	0·6 (0·25, 1·47)
DiNP	487	0·92 (0·47, 1·79)	264	1·09 (0·49, 2·44)	400	0·98 (0·49, 1·96)	336	0·97 (0·46, 2·06)
DnOP	392	1·36 (0·54, 3·44)	262	0·81 (0·31, 2·13)	352	1·2 (0·5, 2·91)	282	1·54 (0·56, 4·26)
DiDP	859	0·87 (0·45, 1·68)	275	1·08 (0·52, 2·25)	717	1·24 (0·7, 2·19)	494	0·78 (0·4, 1·49)

Outcome: Low Birthweight (Preterms only)

	Across Pregnancy		Trimester 1		Trimester 2		Trimester 3	
**Exposure**	**N**	**OR (95% CI)**	**N**	**OR (95% CI)**	**N**	**OR (95% CI)**	**N**	**OR (95% CI)**
LMW	80	2·02 (0·67, 6·05)	18	2·2 (0·05, 95·24)	54	1·82 (0·5, 6·61)	49	2·04 (0·51, 8·21)
HMW	81	0·68 (0·14, 3·34)	20	0·6 (0·01, 26·45)	54	0·39 (0·02, 6·2)	48	1·48 (0·26, 8·41)
DEHP	81	0·5 (0·11, 2·26)	20	0·35 (0·01, 23·45)	54	0·31 (0·02, 4·86)	48	1·39 (0·26, 7·49)
PA	35	0·63 (0·12, 3·33)	18	0·93 (0·16, 5·34)	27	1·62 (0·08, 34·97)	21	0·42 (0·04, 4·63)
DiNP	36	1·16 (0·27, 5·03)	17	0·76 (0·11, 5·21)	28	2·57 (0·31, 20·96)	22	0·78 (0·13, 4·79)
DnOP	34	0·78 (0·08, 7·17)	17	0·43 (0·05, 4·04)	27	3·86 (0·34, 43·92)	21	0·01 (0, 1·47)
DiDP	65	0·25 (0·05, 1·29)	18	0·08 (0, 3·32)	54	1·32 (0·32, 5·49)	34	0·15 (0·02, 1·25)

Outcome: Low Birthweight (Terms only)

	Across Pregnancy		Trimester 1		Trimester 2		Trimester 3	
**Exposure**	**N**	**OR (95% CI)**	**N**	**OR (95% CI)**	**N**	**OR (95% CI)**	**N**	**OR (95% CI)**
LMW	1076	0·64 (0·26, 1·55)	258	1·38 (0·42, 4·61)	679	0·55 (0·19, 1·59)	735	0·57 (0·23, 1·38)
HMW	1073	1·38 (0·53, 3·58)	276	1·89 (0·61, 5·88)	672	1·43 (0·44, 4·64)	719	0·65 (0·21, 2·01)
DEHP	1073	1·26 (0·5, 3·14)	276	2·25 (0·77, 6·53)	672	1·12 (0·34, 3·67)	719	0·71 (0·24, 2·06)
PA	383	0·39 (0·08, 1·83)	255	1·47 (0·38, 5·69)	336	0·95 (0·25, 3·57)	275	**0·2 (0·06, 0·69)**
DiNP	451	0·49 (0·15, 1·58)	247	0·88 (0·3, 2·6)	372	0·8 (0·3, 2·13)	314	0·51 (0·16, 1·64)
DnOP	358	0·82 (0·17, 3·87)	245	0·76 (0·22, 2·57)	325	0·69 (0·18, 2·7)	261	1·15 (0·26, 5·13)
DiDP	794	1·14 (0·43, 3·02)	257	1·6 (0·6, 4·28)	663	0·88 (0·39, 2)	460	0·87 (0·34, 2·22)

Supplement Table 5a1: Education-Stratified Adjusted Associations of Phthalate Exposures with Birth Outcomes (Continuously Measured), High School Education/GED or less Only

Models adjusted for maternal age, race/ethnicity and parity, and child sex· BW for GA model removes parity from covariates·

Outcome: Gestational Age

	Across Pregnancy		Trimester 1		Trimester 2		Trimester 3	
**Exposure**	**N**	**Beta (95% CI)**	**N**	**Beta (95% CI)**	**N**	**Beta (95% CI)**	**N**	**Beta (95% CI)**
LMW	1592	**0·215 (0·004, 0·426)**	285	-0·155 (-0·61, 0·3)	1012	**0·286 (0·014, 0·558)**	1283	0·163 (-0·024, 0·35)
HMW	1618	-0·006 (-0·254, 0·241)	341	-0·278 (-0·781, 0·224)	991	0·288 (-0·03, 0·606)	1171	0·082 (-0·162, 0·326)
DEHP	1622	-0·038 (-0·267, 0·192)	354	-0·268 (-0·763, 0·227)	993	0·229 (-0·065, 0·524)	1177	-0·017 (-0·238, 0·203)
PA	1040	-0·137 (-0·479, 0·205)	240	-0·049 (-0·575, 0·478)	867	0·164 (-0·173, 0·501)	842	-0·117 (-0·402, 0·168)
DiNP	904	**-0·428 (-0·695, -0·16)**	212	0·036 (-0·412, 0·484)	743	0·005 (-0·263, 0·273)	359	-0·258 (-0·588, 0·073)
DnOP	765	**-0·677 (-1·134, -0·221)**	207	0·183 (-0·349, 0·714)	655	-0·123 (-0·536, 0·291)	321	**-0·568 (-1·033, -0·104)**
DiDP	1211	**-0·271 (-0·528, -0·014)**	242	0·034 (-0·358, 0·426)	952	0·08 (-0·183, 0·344)	951	**-0·247 (-0·458, -0·036)**

Outcome: Birth Length

	Across Pregnancy		Trimester 1		Trimester 2		Trimester 3	
**Exposure**	**N**	**Beta (95% CI)**	**N**	**Beta (95% CI)**	**N**	**Beta (95% CI)**	**N**	**Beta (95% CI)**
LMW	1377	0·329 (-0·037, 0·694)	205	**-0·826 (-1·609, -0·044)**	899	**0·622 (0·175, 1·068)**	1155	0·15 (-0·174, 0·474)
HMW	1430	0·253 (-0·16, 0·666)	271	-0·377 (-1·239, 0·484)	889	**0·672 (0·152, 1·192)**	1056	0·318 (-0·094, 0·731)
DEHP	1434	0·315 (-0·07, 0·7)	282	-0·065 (-0·909, 0·779)	891	**0·707 (0·228, 1·186)**	1062	0·322 (-0·055, 0·699)
PA	883	0·053 (-0·561, 0·667)	179	0·037 (-0·814, 0·889)	781	0·381 (-0·184, 0·947)	748	-0·05 (-0·536, 0·437)
DiNP	816	**-0·538 (-0·968, -0·107)**	184	0·284 (-0·432, 1)	683	-0·046 (-0·475, 0·383)	292	-0·213 (-0·739, 0·312)
DnOP	678	-0·3 (-1·04, 0·44)	179	0·174 (-0·68, 1·028)	597	0·002 (-0·647, 0·651)	252	-0·04 (-0·763, 0·683)
DiDP	1061	-0·437 (-0·886, 0·012)	184	0·21 (-0·406, 0·826)	869	0·217 (-0·213, 0·648)	858	**-0·479 (-0·83, -0·128)**

Outcome: Birthweight

	Across Pregnancy		Trimester 1		Trimester 2		Trimester 3	
**Exposure**	**N**	**Beta (95% CI)**	**N**	**Beta (95% CI)**	**N**	**Beta (95% CI)**	**N**	**Beta (95% CI)**
LMW	1592	40·007 (-18·676, 98·691)	285	-69·821 (-197·89, 58·249)	1012	33·03 (-41·71, 107·77)	1283	25·678 (-29·489, 80·846)
HMW	1618	33·584 (-35·196, 102·363)	341	60·059 (-82·191, 202·309)	991	22·909 (-64·64, 110·458)	1171	53·274 (-18·154, 124·702)
DEHP	1622	24·647 (-39·262, 88·557)	354	68·327 (-73·055, 209·709)	993	33·648 (-47·335, 114·631)	1177	17·889 (-46·88, 82·659)
PA	1040	-73·365 (-168·065, 21·335)	240	-10·28 (-161·319, 140·758)	867	12·804 (-80·945, 106·552)	842	-38·438 (-122·546, 45·669)
DiNP	904	-72·011 (-145·586, 1·565)	212	91·353 (-35·525, 218·231)	743	14·8 (-59·186, 88·786)	359	-27·168 (-119·942, 65·606)
DnOP	765	**-142·419 (-265·26, -19·578)**	207	41·429 (-109·815, 192·674)	655	1·017 (-111·53, 113·564)	321	**-144·31 (-272·333, -16·288)**
DiDP	1211	**-103·819 (-174·81, -32·828)**	242	-0·048 (-112·542, 112·446)	952	12·328 (-60·329, 84·985)	951	**-84·102 (-146·635, -21·568)**

Outcome: Birthweight for Gestational Age Z-Score

	Across Pregnancy		Trimester 1		Trimester 2		Trimester 3	
**Exposure**	**N**	**Beta (95% CI)**	**N**	**Beta (95% CI)**	**N**	**Beta (95% CI)**	**N**	**Beta (95% CI)**
LMW	1558	0·056 (-0·067, 0·178)	258	-0·075 (-0·344, 0·193)	982	0·015 (-0·137, 0·167)	1252	0·062 (-0·061, 0·186)
HMW	1583	0·117 (-0·027, 0·26)	313	0·247 (-0·044, 0·537)	961	-0·088 (-0·266, 0·089)	1140	**0·175 (0·015, 0·334)**
DEHP	1587	0·113 (-0·021, 0·248)	326	0·242 (-0·052, 0·537)	963	-0·012 (-0·177, 0·154)	1146	0·121 (-0·025, 0·266)
PA	1009	-0·089 (-0·27, 0·092)	213	-0·034 (-0·33, 0·261)	837	-0·054 (-0·236, 0·128)	814	0·041 (-0·137, 0·219)
DiNP	873	0·049 (-0·09, 0·189)	185	0·21 (-0·046, 0·465)	713	0·059 (-0·083, 0·201)	331	0·14 (-0·055, 0·336)
DnOP	734	-0·047 (-0·281, 0·188)	180	-0·013 (-0·34, 0·313)	625	0·019 (-0·2, 0·238)	293	-0·083 (-0·361, 0·195)
DiDP	1180	-0·132 (-0·273, 0·008)	215	-0·028 (-0·248, 0·191)	922	0·04 (-0·108, 0·187)	923	-0·097 (-0·23, 0·036)

Supplement Table 5b1: Education-Stratified Adjusted Associations of Phthalate Exposures with Categorical Birth Outcomes, High School Education/GED or less Only

Models adjusted for maternal age, race/ethnicity and parity, and child sex·

Outcome: Preterm

	Across Pregnancy		Trimester 1		Trimester 2		Trimester 3	
**Exposure**	**N**	**OR (95% CI)**	**N**	**OR (95% CI)**	**N**	**OR (95% CI)**	**N**	**OR (95% CI)**
LMW	1592	0·75 (0·5, 1·13)	285	1·09 (0·47, 2·55)	1012	**0·57 (0·33, 0·99)**	1283	0·82 (0·54, 1·26)
HMW	1618	1·22 (0·77, 1·94)	341	1·05 (0·38, 2·89)	991	**0·45 (0·25, 0·83)**	1171	1·66 (0·99, 2·77)
DEHP	1622	1·26 (0·82, 1·92)	354	0·84 (0·31, 2·23)	993	**0·53 (0·3, 0·93)**	1177	**1·73 (1·11, 2·72)**
PA	1040	1·11 (0·61, 2·02)	240	0·8 (0·3, 2·11)	867	0·62 (0·33, 1·19)	842	1·29 (0·68, 2·46)
DiNP	904	1·49 (0·94, 2·36)	212	0·98 (0·42, 2·3)	743	0·65 (0·38, 1·09)	359	1·47 (0·79, 2·71)
DnOP	765	**2·8 (1·44, 5·44)**	207	0·57 (0·19, 1·7)	655	1·1 (0·54, 2·22)	321	**3·03 (1·43, 6·4)**
DiDP	1211	1·1 (0·67, 1·8)	242	0·5 (0·22, 1·15)	952	0·79 (0·47, 1·34)	951	1·47 (0·91, 2·37)

Outcome: Small for Gestational Age

	Across Pregnancy		Trimester 1		Trimester 2		Trimester 3	
**Exposure**	**N**	**OR (95% CI)**	**N**	**OR (95% CI)**	**N**	**OR (95% CI)**	**N**	**OR (95% CI)**
LMW	1589	0·76 (0·54, 1·07)	285	1·17 (0·57, 2·4)	1012	0·65 (0·42, 1·02)	1280	0·72 (0·52, 1·02)
HMW	1614	0·79 (0·53, 1·18)	340	**0·38 (0·16, 0·89)**	991	1·2 (0·74, 1·94)	1168	0·84 (0·54, 1·29)
DEHP	1618	0·78 (0·54, 1·13)	353	**0·39 (0·17, 0·92)**	993	0·98 (0·63, 1·54)	1174	0·89 (0·61, 1·32)
PA	1040	1 (0·59, 1·7)	240	1·29 (0·56, 2·98)	867	0·92 (0·54, 1·54)	842	0·7 (0·43, 1·14)
DiNP	904	0·81 (0·54, 1·22)	212	0·65 (0·3, 1·42)	743	0·92 (0·61, 1·38)	359	0·77 (0·46, 1·28)
DnOP	765	0·81 (0·41, 1·59)	207	0·77 (0·33, 1·8)	655	0·88 (0·48, 1·63)	321	0·96 (0·48, 1·93)
DiDP	1211	1·15 (0·77, 1·7)	242	1·09 (0·59, 2·03)	952	0·99 (0·65, 1·5)	951	1·17 (0·81, 1·68)

Outcome: Large for Gestational Age

	Across Pregnancy		Trimester 1		Trimester 2		Trimester 3	
**Exposure**	**N**	**OR (95% CI)**	**N**	**OR (95% CI)**	**N**	**OR (95% CI)**	**N**	**OR (95% CI)**
LMW	1589	1·1 (0·73, 1·64)	285	1·41 (0·59, 3·37)	1012	0·73 (0·41, 1·31)	1280	0·94 (0·62, 1·43)
HMW	1614	1·26 (0·79, 2)	340	2·03 (0·82, 5·05)	991	0·76 (0·4, 1·46)	1168	1·31 (0·78, 2·2)
DEHP	1618	1·11 (0·71, 1·71)	353	2·01 (0·85, 4·75)	993	0·71 (0·39, 1·31)	1174	1·04 (0·64, 1·69)
PA	1040	1 (0·53, 1·9)	240	2·05 (0·72, 5·78)	867	1·15 (0·59, 2·26)	842	0·76 (0·4, 1·44)
DiNP	904	1·06 (0·62, 1·79)	212	**2·24 (1·13, 4·43)**	743	0·93 (0·54, 1·62)	359	0·8 (0·41, 1·58)
DnOP	765	1·31 (0·57, 2·98)	207	2·24 (0·93, 5·42)	655	1·08 (0·49, 2·35)	321	1·13 (0·45, 2·85)
DiDP	1211	0·64 (0·38, 1·08)	242	1·42 (0·67, 2·97)	952	0·76 (0·46, 1·26)	951	0·81 (0·47, 1·39)

Outcome: Low Birthweight

	Across Pregnancy		Trimester 1		Trimester 2		Trimester 3	
**Exposure**	**N**	**OR (95% CI)**	**N**	**OR (95% CI)**	**N**	**OR (95% CI)**	**N**	**OR (95% CI)**
LMW	1592	0·68 (0·43, 1·1)	285	1·78 (0·74, 4·28)	1012	0·73 (0·42, 1·3)	1283	0·65 (0·39, 1·08)
HMW	1618	1 (0·59, 1·69)	341	1·53 (0·61, 3·87)	991	0·8 (0·43, 1·51)	1171	0·84 (0·45, 1·59)
DEHP	1622	1 (0·62, 1·62)	354	1·41 (0·58, 3·4)	993	0·78 (0·43, 1·4)	1177	1·14 (0·66, 1·96)
PA	1040	1·53 (0·81, 2·91)	240	1·84 (0·64, 5·3)	867	1·11 (0·58, 2·11)	842	0·82 (0·42, 1·6)
DiNP	904	1·58 (0·98, 2·53)	212	0·77 (0·3, 1·93)	743	0·82 (0·49, 1·36)	359	1·29 (0·69, 2·41)
DnOP	765	1·66 (0·83, 3·32)	207	0·74 (0·26, 2·14)	655	0·68 (0·32, 1·43)	321	**2·26 (1·07, 4·8)**
DiDP	1211	**1·97 (1·17, 3·32)**	242	1·25 (0·59, 2·65)	952	0·7 (0·42, 1·18)	951	**2·12 (1·28, 3·51)**

Outcome: Low Birthweight (Preterms only)

	Across Pregnancy		Trimester 1		Trimester 2		Trimester 3	
**Exposure**	**N**	**OR (95% CI)**	**N**	**OR (95% CI)**	**N**	**OR (95% CI)**	**N**	**OR (95% CI)**
LMW	146	0·95 (0·44, 2·06)	29	0·89 (0·05, 16·89)	90	3·21 (0·86, 12·01)	104	1·02 (0·46, 2·28)
HMW	145	1·24 (0·53, 2·92)	29	5·52 (0·09, 353·47)	89	1·87 (0·51, 6·84)	95	1·39 (0·5, 3·85)
DEHP	147	1·1 (0·49, 2·46)	33	0·59 (0·01, 35·96)	90	1·23 (0·38, 3·98)	96	1·82 (0·69, 4·79)
PA	99	**3·62 (1·03, 12·75)**	21	2·55 (0·1, 65·99)	73	2·89 (0·47, 17·7)	71	**4·97 (1·1, 22·43)**
DiNP	83	**2·59 (1, 6·69)**			64	2·11 (0·62, 7·19)	36	3·08 (0·88, 10·77)
DnOP	80	1·48 (0·4, 5·49)			61	0·96 (0·24, 3·79)	37	2·99 (0·65, 13·7)
DiDP	108	**3·89 (1·47, 10·32)**	20	4·58 (0·06, 348·53)	80	1·49 (0·52, 4·25)	76	2·87 (0·96, 8·64)

Outcome: Low Birthweight (Terms only)

	Across Pregnancy		Trimester 1		Trimester 2		Trimester 3	
**Exposure**	**N**	**OR (95% CI)**	**N**	**OR (95% CI)**	**N**	**OR (95% CI)**	**N**	**OR (95% CI)**
LMW	1446	0·6 (0·3, 1·22)	256	1·94 (0·6, 6·21)	922	0·65 (0·28, 1·53)	1179	**0·46 (0·22, 0·96)**
HMW	1473	0·73 (0·33, 1·6)	312	1·66 (0·51, 5·43)	902	1·09 (0·44, 2·7)	1076	0·43 (0·17, 1·08)
DEHP	1475	0·84 (0·41, 1·72)	321	2·1 (0·74, 5·98)	903	1·19 (0·52, 2·73)	1081	0·65 (0·29, 1·43)
PA	941	1·17 (0·44, 3·07)	219	2·64 (0·64, 10·85)	794	1·67 (0·67, 4·17)	771	**0·27 (0·11, 0·64)**
DiNP	821	0·92 (0·42, 2·02)			679	0·77 (0·37, 1·61)	323	0·59 (0·24, 1·44)
DnOP	685	0·76 (0·23, 2·55)			594	0·43 (0·13, 1·37)	284	1·12 (0·36, 3·52)
DiDP	1103	1·39 (0·63, 3·07)	222	1·58 (0·59, 4·22)	872	**0·44 (0·21, 0·92)**	875	1·54 (0·75, 3·14)

Supplement Table 5a2: Education-Stratified Adjusted Associations of Phthalate Exposures with Birth Outcomes (Continuously Measured), Some College or more Only

Models adjusted for maternal age, race/ethnicity and parity, and child sex· BW for GA model removes parity from covariates·

Outcome: Gestational Age

	Across Pregnancy		Trimester 1		Trimester 2		Trimester 3	
**Exposure**	**N**	**Beta (95% CI)**	**N**	**Beta (95% CI)**	**N**	**Beta (95% CI)**	**N**	**Beta (95% CI)**
LMW	2797	**-0·153 (-0·302, -0·004)**	714	-0·025 (-0·248, 0·197)	2112	-0·043 (-0·189, 0·103)	1814	**-0·172 (-0·33, -0·014)**
HMW	2828	**-0·254 (-0·441, -0·068)**	827	**-0·327 (-0·585, -0·069)**	2071	0·058 (-0·114, 0·23)	1660	**-0·312 (-0·536, -0·088)**
DEHP	2832	**-0·231 (-0·409, -0·052)**	843	**-0·297 (-0·545, -0·049)**	2076	0·04 (-0·126, 0·205)	1667	**-0·269 (-0·48, -0·057)**
PA	1567	**-0·878 (-1·088, -0·668)**	668	0·059 (-0·161, 0·28)	1212	**-0·211 (-0·382, -0·04)**	1301	**-0·645 (-0·818, -0·472)**
DiNP	1941	**-0·468 (-0·62, -0·317)**	620	0·062 (-0·147, 0·271)	1567	-0·019 (-0·153, 0·115)	1041	**-0·639 (-0·821, -0·456)**
DnOP	1272	**-0·658 (-0·962, -0·355)**	608	0·037 (-0·199, 0·274)	1019	0·044 (-0·192, 0·279)	896	**-0·575 (-0·853, -0·297)**
DiDP	2564	**-0·283 (-0·44, -0·125)**	676	0·116 (-0·077, 0·309)	2018	-0·022 (-0·157, 0·114)	1617	**-0·249 (-0·407, -0·091)**

Outcome: Birth Length

	Across Pregnancy		Trimester 1		Trimester 2		Trimester 3	
**Exposure**	**N**	**Beta (95% CI)**	**N**	**Beta (95% CI)**	**N**	**Beta (95% CI)**	**N**	**Beta (95% CI)**
LMW	2344	**-0·303 (-0·581, -0·025)**	549	-0·212 (-0·641, 0·217)	1850	-0·124 (-0·394, 0·146)	1502	-0·188 (-0·486, 0·111)
HMW	2421	**-0·367 (-0·707, -0·028)**	688	0·148 (-0·343, 0·639)	1821	0·042 (-0·268, 0·352)	1361	-0·381 (-0·803, 0·041)
DEHP	2425	-0·322 (-0·648, 0·004)	702	-0·02 (-0·489, 0·449)	1826	0·064 (-0·233, 0·361)	1368	-0·31 (-0·708, 0·087)
PA	1171	**-1·282 (-1·726, -0·839)**	512	-0·05 (-0·471, 0·371)	983	-0·001 (-0·33, 0·327)	1024	**-0·967 (-1·335, -0·6)**
DiNP	1640	**-0·958 (-1·232, -0·684)**	526	0·041 (-0·348, 0·43)	1360	-0·106 (-0·351, 0·139)	779	**-1·237 (-1·592, -0·883)**
DnOP	975	**-1·042 (-1·634, -0·451)**	512	0·108 (-0·326, 0·542)	817	0·172 (-0·275, 0·619)	635	**-1·093 (-1·664, -0·521)**
DiDP	2165	**-0·681 (-0·986, -0·376)**	527	0·112 (-0·247, 0·471)	1777	-0·006 (-0·258, 0·247)	1340	**-0·742 (-1·036, -0·447)**

Outcome: Birthweight

	Across Pregnancy		Trimester 1		Trimester 2		Trimester 3	
**Exposure**	**N**	**Beta (95% CI)**	**N**	**Beta (95% CI)**	**N**	**Beta (95% CI)**	**N**	**Beta (95% CI)**
LMW	2797	**-53·66 (-98·12, -9·2)**	714	10·182 (-61·461, 81·826)	2112	-22·714 (-69·484, 24·056)	1814	-45·02 (-92·389, 2·349)
HMW	2828	-9·067 (-64·417, 46·283)	827	28·261 (-55·667, 112·188)	2071	12·975 (-42·046, 67·995)	1660	-29·879 (-96·582, 36·824)
DEHP	2832	-12·11 (-65·192, 40·972)	843	8·985 (-71·154, 89·124)	2076	3·783 (-49·086, 56·651)	1667	-29·547 (-92·523, 33·43)
PA	1567	**-160·18 (-222·057, -98·302)**	668	7·576 (-63·552, 78·705)	1212	-26·55 (-80·708, 27·609)	1301	**-130·13 (-182·356, -77·905)**
DiNP	1941	**-82·657 (-127·079, -38·236)**	620	**71·593 (4·668, 138·518)**	1567	23·829 (-18·42, 66·079)	1041	**-127·871 (-180·518, -75·224)**
DnOP	1272	**-133·029 (-218·442, -47·616)**	608	22·83 (-52·994, 98·654)	1019	4·244 (-68·172, 76·66)	896	**-139·22 (-218·183, -60·258)**
DiDP	2564	-25·892 (-73·261, 21·477)	676	48·65 (-13·647, 110·948)	2018	22·717 (-20·719, 66·154)	1617	-39·667 (-87·589, 8·256)

Outcome: Birthweight for Gestational Age Z-Score

	Across Pregnancy		Trimester 1		Trimester 2		Trimester 3	
**Exposure**	**N**	**Beta (95% CI)**	**N**	**Beta (95% CI)**	**N**	**Beta (95% CI)**	**N**	**Beta (95% CI)**
LMW	2602	**-0·094 (-0·185, -0·002)**	552	-0·036 (-0·209, 0·137)	1934	-0·077 (-0·181, 0·027)	1629	-0·078 (-0·176, 0·019)
HMW	2633	0·087 (-0·024, 0·198)	665	0·149 (-0·05, 0·348)	1893	0·012 (-0·106, 0·131)	1475	0·065 (-0·067, 0·198)
DEHP	2637	0·068 (-0·04, 0·175)	681	0·105 (-0·088, 0·299)	1898	-0·001 (-0·115, 0·114)	1482	0·033 (-0·093, 0·159)
PA	1379	0·03 (-0·091, 0·15)	508	0·029 (-0·146, 0·203)	1038	0·041 (-0·078, 0·16)	1120	-0·013 (-0·116, 0·09)
DiNP	1751	0·031 (-0·058, 0·119)	459	**0·212 (0·048, 0·377)**	1391	0·074 (-0·018, 0·167)	859	-0·017 (-0·119, 0·084)
DnOP	1084	0·003 (-0·16, 0·165)	448	0·044 (-0·148, 0·236)	845	-0·005 (-0·161, 0·15)	715	-0·049 (-0·204, 0·107)
DiDP	2373	0·051 (-0·049, 0·15)	515	0·009 (-0·142, 0·16)	1841	0·035 (-0·064, 0·135)	1435	-0·002 (-0·099, 0·094)

Supplement Table 5b2: Education-Stratified Adjusted Associations of Phthalate Exposures with Categorical Birth Outcomes, Some College or more Only

Models adjusted for maternal age, race/ethnicity and parity, and child sex·

Outcome: Preterm

	Across Pregnancy		Trimester 1		Trimester 2		Trimester 3	
**Exposure**	**N**	**OR (95% CI)**	**N**	**OR (95% CI)**	**N**	**OR (95% CI)**	**N**	**OR (95% CI)**
LMW	2797	1·06 (0·76, 1·47)	714	0·77 (0·37, 1·61)	2112	0·94 (0·63, 1·4)	1814	1·13 (0·8, 1·58)
HMW	2828	1·47 (0·99, 2·19)	827	1·83 (0·75, 4·42)	2071	0·83 (0·52, 1·33)	1660	**1·58 (1·01, 2·46)**
DEHP	2832	1·27 (0·87, 1·87)	843	1·53 (0·68, 3·45)	2076	0·79 (0·5, 1·26)	1667	1·38 (0·9, 2·1)
PA	1567	**3·48 (2·37, 5·13)**	668	0·98 (0·46, 2·09)	1212	1·35 (0·84, 2·16)	1301	**2·83 (1·95, 4·11)**
DiNP	1941	**2·42 (1·81, 3·23)**	620	0·78 (0·38, 1·6)	1567	1·11 (0·78, 1·58)	1041	**2·56 (1·87, 3·5)**
DnOP	1272	**3·56 (2·16, 5·84)**	608	0·84 (0·38, 1·87)	1019	1·35 (0·77, 2·38)	896	**2·56 (1·67, 3·94)**
DiDP	2564	**1·76 (1·26, 2·46)**	676	0·77 (0·4, 1·47)	2018	0·9 (0·63, 1·29)	1617	**1·75 (1·26, 2·44)**

Outcome: Small for Gestational Age

	Across Pregnancy		Trimester 1		Trimester 2		Trimester 3	
**Exposure**	**N**	**OR (95% CI)**	**N**	**OR (95% CI)**	**N**	**OR (95% CI)**	**N**	**OR (95% CI)**
LMW	2793	1·09 (0·82, 1·44)	713	1·05 (0·67, 1·64)	2111	1·09 (0·8, 1·49)	1811	0·98 (0·73, 1·32)
HMW	2824	0·93 (0·65, 1·33)	826	1·06 (0·62, 1·83)	2070	1·12 (0·78, 1·61)	1657	0·95 (0·64, 1·42)
DEHP	2828	0·98 (0·7, 1·38)	842	1·06 (0·64, 1·78)	2075	1·16 (0·82, 1·64)	1664	0·9 (0·62, 1·33)
PA	1567	0·95 (0·65, 1·37)	668	1·16 (0·73, 1·82)	1212	1·04 (0·73, 1·47)	1301	1·14 (0·82, 1·58)
DiNP	1941	1·1 (0·83, 1·46)	620	0·74 (0·48, 1·15)	1567	0·92 (0·69, 1·23)	1041	1·14 (0·84, 1·55)
DnOP	1272	1·19 (0·73, 1·94)	608	0·92 (0·57, 1·47)	1019	0·96 (0·61, 1·52)	896	1·37 (0·89, 2·11)
DiDP	2564	0·9 (0·66, 1·22)	676	1·1 (0·75, 1·61)	2018	0·87 (0·65, 1·16)	1617	0·9 (0·68, 1·21)

Outcome: Large for Gestational Age

	Across Pregnancy		Trimester 1		Trimester 2		Trimester 3	
**Exposure**	**N**	**OR (95% CI)**	**N**	**OR (95% CI)**	**N**	**OR (95% CI)**	**N**	**OR (95% CI)**
LMW	2793	0·95 (0·72, 1·25)	713	1·05 (0·65, 1·7)	2111	0·93 (0·69, 1·24)	1811	1·14 (0·86, 1·5)
HMW	2824	1·05 (0·75, 1·47)	826	1·5 (0·86, 2·61)	2070	0·87 (0·62, 1·23)	1657	0·99 (0·66, 1·47)
DEHP	2828	1·07 (0·77, 1·48)	842	1·41 (0·82, 2·4)	2075	0·89 (0·64, 1·24)	1664	0·98 (0·67, 1·43)
PA	1567	1·22 (0·87, 1·73)	668	1·02 (0·64, 1·61)	1212	1·38 (0·98, 1·94)	1301	1·06 (0·79, 1·4)
DiNP	1941	1·06 (0·82, 1·37)	620	1·31 (0·86, 2)	1567	1·15 (0·89, 1·49)	1041	0·91 (0·67, 1·23)
DnOP	1272	0·92 (0·57, 1·49)	608	0·72 (0·43, 1·2)	1019	0·87 (0·55, 1·4)	896	0·86 (0·57, 1·3)
DiDP	2564	**1·32 (1, 1·74)**	676	1·42 (0·95, 2·13)	2018	1·22 (0·93, 1·59)	1617	1·17 (0·88, 1·55)

Outcome: Low Birthweight

	Across Pregnancy		Trimester 1		Trimester 2		Trimester 3	
**Exposure**	**N**	**OR (95% CI)**	**N**	**OR (95% CI)**	**N**	**OR (95% CI)**	**N**	**OR (95% CI)**
LMW	2797	**1·52 (1·07, 2·17)**	714	0·72 (0·34, 1·54)	2112	1·19 (0·76, 1·85)	1814	1·27 (0·89, 1·82)
HMW	2828	1·5 (0·95, 2·34)	827	1·2 (0·47, 3·1)	2071	0·96 (0·57, 1·64)	1660	1·34 (0·83, 2·15)
DEHP	2832	1·46 (0·95, 2·24)	843	1·2 (0·49, 2·91)	2076	1·01 (0·61, 1·69)	1667	1·3 (0·83, 2·03)
PA	1567	**3·16 (2·09, 4·79)**	668	1·01 (0·48, 2·13)	1212	1·24 (0·74, 2·06)	1301	**2·64 (1·77, 3·93)**
DiNP	1941	**2·17 (1·55, 3·04)**	620	0·59 (0·27, 1·3)	1567	0·95 (0·63, 1·44)	1041	**2·2 (1·57, 3·09)**
DnOP	1272	**2·62 (1·53, 4·51)**	608	0·59 (0·25, 1·37)	1019	0·99 (0·52, 1·87)	896	**2·01 (1·26, 3·19)**
DiDP	2564	1·34 (0·91, 1·98)	676	0·64 (0·32, 1·28)	2018	0·95 (0·62, 1·43)	1617	1·3 (0·91, 1·85)

Outcome: Low Birthweight (Preterms only)

	Across Pregnancy		Trimester 1		Trimester 2		Trimester 3	
Exposure	N	OR (95% CI)	N	OR (95% CI)	N	OR (95% CI)	N	OR (95% CI)
LMW	199	1·59 (0·87, 2·9)	31	1·82 (0·22, 15·05)	129	1·12 (0·5, 2·49)	126	0·87 (0·45, 1·65)
HMW	199	0·88 (0·35, 2·24)	30	0 (0, 50690·65)	127	1·08 (0·36, 3·27)	122	0·58 (0·22, 1·54)
DEHP	199	1·04 (0·43, 2·49)	33	0 (0, 1·15)	127	1·59 (0·52, 4·87)	122	0·73 (0·3, 1·81)
PA	127	1·39 (0·73, 2·65)			71	0·63 (0·2, 2·02)	98	1·39 (0·74, 2·64)
DiNP	150	1·65 (0·93, 2·93)	25	0·18 (0, 8·19)	95	0·8 (0·31, 2·03)	92	1·07 (0·6, 1·92)
DnOP	111	0·77 (0·3, 2·02)			61	0·32 (0·07, 1·58)	86	0·81 (0·34, 1·93)
DiDP	187	0·95 (0·52, 1·74)			125	0·67 (0·28, 1·61)	115	0·62 (0·32, 1·19)

Outcome: Low Birthweight (Terms only)

	Across Pregnancy		Trimester 1		Trimester 2		Trimester 3	
Exposure	N	OR (95% CI)	N	OR (95% CI)	N	OR (95% CI)	N	OR (95% CI)
LMW	2598	1·71 (0·97, 3·01)	683	0·86 (0·3, 2·43)	1983	1·29 (0·65, 2·57)	1688	1·67 (0·96, 2·93)
HMW	2629	1·59 (0·78, 3·21)	797	1·91 (0·62, 5·89)	1944	1·06 (0·47, 2·38)	1538	1·38 (0·64, 2·95)
DEHP	2633	1·69 (0·88, 3·24)	810	2·42 (0·84, 6·94)	1949	1·12 (0·52, 2·41)	1545	1·4 (0·69, 2·83)
PA	1440	1·74 (0·76, 3·97)	640	1·18 (0·45, 3·11)	1141	1·36 (0·61, 3·04)	1203	1·69 (0·82, 3·48)
DiNP	1791	1·16 (0·63, 2·14)	595	0·48 (0·17, 1·39)	1472	0·87 (0·47, 1·62)	949	1·43 (0·78, 2·61)
DnOP	1161	1·47 (0·52, 4·16)	583	0·56 (0·19, 1·63)	958	1 (0·37, 2·67)	810	1·47 (0·63, 3·44)
DiDP	2377	0·9 (0·47, 1·76)	648	0·9 (0·36, 2·24)	1893	0·97 (0·52, 1·81)	1502	1·19 (0·67, 2·13)

Supplement Table 6a1: Parity-Stratified Adjusted Associations of Phthalate Exposures with Birth Outcomes (Continuously Measured), Parity=0 Only

Models adjusted for maternal age, race/ethnicity and education, and child sex· BW for GA model removes parity from covariates·

Outcome: Gestational Age

	Across Pregnancy		Trimester 1		Trimester 2		Trimester 3	
**Exposure**	**N**	**Beta (95% CI)**	**N**	**Beta (95% CI)**	**N**	**Beta (95% CI)**	**N**	**Beta (95% CI)**
LMW	1801	0·02 (-0·172, 0·211)	364	-0·121 (-0·442, 0·2)	1251	0·144 (-0·069, 0·356)	1214	-0·168 (-0·369, 0·034)
HMW	1810	-0·145 (-0·389, 0·099)	418	**-0·489 (-0·885, -0·092)**	1226	0·12 (-0·134, 0·374)	1094	-0·143 (-0·41, 0·123)
DEHP	1812	-0·086 (-0·319, 0·146)	432	-0·376 (-0·77, 0·018)	1228	0·133 (-0·112, 0·378)	1097	-0·096 (-0·347, 0·155)
PA	1000	**-0·733 (-1·044, -0·422)**	326	0·161 (-0·153, 0·474)	792	-0·174 (-0·444, 0·095)	803	**-0·711 (-0·975, -0·447)**
DiNP	1160	**-0·512 (-0·725, -0·298)**	282	0·104 (-0·221, 0·429)	933	-0·094 (-0·295, 0·108)	504	**-0·667 (-0·939, -0·395)**
DnOP	761	**-0·862 (-1·317, -0·408)**	279	-0·021 (-0·393, 0·351)	633	0·053 (-0·317, 0·424)	405	**-0·897 (-1·349, -0·444)**
DiDP	1573	**-0·31 (-0·528, -0·091)**	328	0·226 (-0·044, 0·496)	1218	-0·058 (-0·264, 0·148)	1019	**-0·215 (-0·425, -0·006)**

Outcome: Birth Length

	Across Pregnancy		Trimester 1		Trimester 2		Trimester 3	
**Exposure**	**N**	**Beta (95% CI)**	**N**	**Beta (95% CI)**	**N**	**Beta (95% CI)**	**N**	**Beta (95% CI)**
LMW	1566	-0·116 (-0·44, 0·209)	274	-0·134 (-0·768, 0·501)	1134	-0·083 (-0·434, 0·268)	1075	-0·157 (-0·489, 0·176)
HMW	1601	-0·128 (-0·531, 0·275)	346	-0·045 (-0·767, 0·677)	1116	-0·001 (-0·415, 0·412)	964	0·074 (-0·365, 0·514)
DEHP	1603	-0·038 (-0·423, 0·347)	357	-0·239 (-0·945, 0·466)	1118	0·082 (-0·317, 0·482)	967	0·144 (-0·269, 0·558)
PA	811	**-1·121 (-1·693, -0·549)**	250	0·004 (-0·583, 0·591)	694	-0·061 (-0·511, 0·389)	691	**-1·02 (-1·494, -0·547)**
DiNP	1039	**-0·826 (-1·17, -0·482)**	254	0·128 (-0·475, 0·731)	849	-0·135 (-0·467, 0·196)	414	**-0·987 (-1·441, -0·532)**
DnOP	645	**-1·059 (-1·823, -0·296)**	250	-0·032 (-0·658, 0·595)	554	0·353 (-0·263, 0·969)	314	**-1·195 (-1·984, -0·405)**
DiDP	1380	**-0·716 (-1·106, -0·326)**	255	-0·036 (-0·524, 0·451)	1114	-0·095 (-0·433, 0·242)	905	**-0·492 (-0·835, -0·149)**

Outcome: Birthweight

	Across Pregnancy		Trimester 1		Trimester 2		Trimester 3	
**Exposure**	**N**	**Beta (95% CI)**	**N**	**Beta (95% CI)**	**N**	**Beta (95% CI)**	**N**	**Beta (95% CI)**
LMW	1801	-4·399 (-56·703, 47·905)	364	-1·105 (-97·237, 95·027)	1251	-8·357 (-67·74, 51·026)	1214	-26·399 (-83·126, 30·328)
HMW	1810	10·159 (-56·414, 76·732)	418	8·529 (-111·176, 128·235)	1226	22·101 (-48·671, 92·874)	1094	11·976 (-62·924, 86·877)
DEHP	1812	22·126 (-41·357, 85·61)	432	16·019 (-102·629, 134·667)	1228	25·787 (-42·537, 94·11)	1097	18·357 (-52·132, 88·846)
PA	1000	**-143·371 (-224·675, -62·067)**	326	18·082 (-76·835, 112·999)	792	-18·384 (-91·161, 54·394)	803	**-149·644 (-222·472, -76·817)**
DiNP	1160	**-95·822 (-152·182, -39·462)**	282	92·979 (-5·24, 191·197)	933	5·552 (-49·462, 60·566)	504	**-128·083 (-199·852, -56·315)**
DnOP	761	**-174·569 (-288·327, -60·811)**	279	31·724 (-80·785, 144·234)	633	25·757 (-71·372, 122·886)	405	**-180·587 (-296·856, -64·318)**
DiDP	1573	**-80·307 (-139·694, -20·919)**	328	-13·388 (-96·042, 69·266)	1218	-12·65 (-70·17, 44·871)	1019	-40·555 (-99·223, 18·114)

Outcome: Birthweight for Gestational Age Z-Score

	Across Pregnancy		Trimester 1		Trimester 2		Trimester 3	
**Exposure**	**N**	**Beta (95% CI)**	**N**	**Beta (95% CI)**	**N**	**Beta (95% CI)**	**N**	**Beta (95% CI)**
LMW	1796	-0·015 (-0·122, 0·092)	363	0·045 (-0·165, 0·254)	1249	-0·084 (-0·207, 0·039)	1211	0·006 (-0·11, 0·122)
HMW	1804	0·1 (-0·034, 0·235)	416	0·2 (-0·052, 0·452)	1224	0 (-0·146, 0·146)	1091	0·113 (-0·038, 0·264)
DEHP	1806	0·102 (-0·026, 0·231)	430	0·17 (-0·083, 0·423)	1226	0·004 (-0·137, 0·145)	1094	0·106 (-0·037, 0·248)
PA	1000	-0·004 (-0·155, 0·147)	326	-0·05 (-0·254, 0·154)	792	0·016 (-0·128, 0·16)	803	-0·035 (-0·175, 0·105)
DiNP	1160	0·022 (-0·086, 0·13)	282	0·189 (-0·022, 0·4)	933	0·063 (-0·049, 0·175)	504	0·014 (-0·113, 0·142)
DnOP	761	0·019 (-0·181, 0·219)	279	0·083 (-0·158, 0·323)	633	0·075 (-0·109, 0·26)	405	0·013 (-0·182, 0·209)
DiDP	1572	-0·04 (-0·158, 0·077)	328	-0·15 (-0·327, 0·028)	1217	0·009 (-0·109, 0·128)	1019	0·017 (-0·097, 0·131)

Supplement Table 6b1: Parity-Stratified Adjusted Associations of Phthalate Exposures with Categorical Birth Outcomes, Parity=0 Only

Models adjusted for maternal age, race/ethnicity and education, and child sex·

Outcome: Preterm

	Across Pregnancy		Trimester 1		Trimester 2		Trimester 3	
**Exposure**	**N**	**OR (95% CI)**	**N**	**OR (95% CI)**	**N**	**OR (95% CI)**	**N**	**OR (95% CI)**
LMW	1801	0·79 (0·52, 1·19)	364	1·17 (0·38, 3·59)	1251	**0·55 (0·33, 0·93)**	1214	1·04 (0·68, 1·61)
HMW	1810	1·41 (0·88, 2·26)	418	2·21 (0·67, 7·3)	1226	0·74 (0·42, 1·31)	1094	1·7 (0·99, 2·9)
DEHP	1812	1·25 (0·8, 1·97)	432	1·32 (0·41, 4·23)	1228	0·71 (0·41, 1·23)	1097	1·52 (0·92, 2·5)
PA	1000	**3·29 (1·9, 5·7)**	326	0·5 (0·18, 1·37)	792	0·99 (0·51, 1·92)	803	**3·31 (1·88, 5·83)**
DiNP	1160	**2·73 (1·88, 3·95)**	282	0·78 (0·38, 1·6)	933	1·4 (0·92, 2·14)	504	**3·02 (1·89, 4·83)**
DnOP	761	**6·15 (3·06, 12·35)**	279	0·84 (0·38, 1·87)	633	1·6 (0·72, 3·55)	405	**4·54 (2·33, 8·83)**
DiDP	1573	**2·37 (1·55, 3·61)**	328	0·44 (0·15, 1·26)	1218	1·48 (0·95, 2·33)	1019	**1·85 (1·21, 2·82)**

Outcome: Small for Gestational Age

	Across Pregnancy		Trimester 1		Trimester 2		Trimester 3	
**Exposure**	**N**	**OR (95% CI)**	**N**	**OR (95% CI)**	**N**	**OR (95% CI)**	**N**	**OR (95% CI)**
LMW	1797	0·95 (0·7, 1·29)	363	1·07 (0·62, 1·86)	1250	1·01 (0·7, 1·44)	1211	0·88 (0·64, 1·22)
HMW	1805	0·87 (0·59, 1·28)	416	0·89 (0·46, 1·75)	1225	1·07 (0·71, 1·63)	1091	0·95 (0·63, 1·44)
DEHP	1807	0·88 (0·61, 1·27)	430	0·89 (0·46, 1·72)	1227	1·06 (0·71, 1·58)	1094	0·93 (0·63, 1·37)
PA	1000	1·05 (0·67, 1·63)	326	1·42 (0·8, 2·52)	792	1·16 (0·76, 1·79)	803	1·13 (0·75, 1·72)
DiNP	1160	0·89 (0·64, 1·25)	282	0·64 (0·34, 1·19)	933	0·96 (0·68, 1·35)	504	0·95 (0·64, 1·42)
DnOP	761	0·87 (0·47, 1·61)	279	0·96 (0·5, 1·84)	633	0·9 (0·51, 1·58)	405	0·96 (0·52, 1·77)
DiDP	1573	0·9 (0·64, 1·27)	328	1·13 (0·7, 1·81)	1218	0·97 (0·68, 1·36)	1019	0·84 (0·6, 1·16)

Outcome: Large for Gestational Age

	Across Pregnancy		Trimester 1		Trimester 2		Trimester 3	
**Exposure**	**N**	**OR (95% CI)**	**N**	**OR (95% CI)**	**N**	**OR (95% CI)**	**N**	**OR (95% CI)**
LMW	1797	1·36 (0·93, 1·98)	363	1·7 (0·81, 3·56)	1250	1·09 (0·69, 1·7)	1211	**1·61 (1·04, 2·49)**
HMW	1805	1·17 (0·71, 1·93)	416	**3·32 (1·28, 8·58)**	1225	0·98 (0·57, 1·68)	1091	1·21 (0·68, 2·17)
DEHP	1807	1·26 (0·78, 2·01)	430	**3·08 (1·23, 7·69)**	1227	1·07 (0·64, 1·8)	1094	1·14 (0·66, 1·98)
PA	1000	1·16 (0·63, 2·16)	326	2·11 (0·9, 4·97)	792	1·69 (0·92, 3·1)	803	0·84 (0·46, 1·54)
DiNP	1160	0·86 (0·57, 1·32)	282	**2·29 (1·1, 4·76)**	933	1·13 (0·76, 1·7)	504	0·7 (0·38, 1·32)
DnOP	761	0·56 (0·22, 1·42)	279	1·53 (0·62, 3·8)	633	1·15 (0·53, 2·49)	405	0·54 (0·2, 1·43)
DiDP	1573	0·85 (0·54, 1·34)	328	1·23 (0·64, 2·35)	1218	1·17 (0·76, 1·8)	1019	0·82 (0·48, 1·38)

Outcome: Low Birthweight

	Across Pregnancy		Trimester 1		Trimester 2		Trimester 3	
**Exposure**	**N**	**OR (95% CI)**	**N**	**OR (95% CI)**	**N**	**OR (95% CI)**	**N**	**OR (95% CI)**
LMW	1801	1·32 (0·89, 1·98)	364	1·58 (0·67, 3·72)	1251	1·25 (0·76, 2·07)	1214	1·25 (0·82, 1·91)
HMW	1810	1·5 (0·92, 2·47)	418	2·29 (0·85, 6·16)	1226	1·11 (0·61, 2)	1094	1·29 (0·74, 2·23)
DEHP	1812	1·39 (0·87, 2·22)	432	1·97 (0·77, 5·06)	1228	1·02 (0·58, 1·8)	1097	1·3 (0·78, 2·17)
PA	1000	**3·08 (1·78, 5·31)**	326	1·57 (0·63, 3·94)	792	1·32 (0·7, 2·48)	803	**2·27 (1·31, 3·91)**
DiNP	1160	**1·94 (1·28, 2·94)**	282	0·34 (0·11, 1·11)	933	0·96 (0·59, 1·57)	504	**2·24 (1·41, 3·55)**
DnOP	761	**2·99 (1·49, 6·01)**	279	0·61 (0·19, 1·97)	633	0·85 (0·37, 1·92)	405	**2·53 (1·33, 4·84)**
DiDP	1573	1·28 (0·81, 2·03)	328	0·68 (0·31, 1·48)	1218	0·91 (0·56, 1·47)	1019	1·11 (0·72, 1·71)

Outcome: Low Birthweight (Preterms only)

	Across Pregnancy		Trimester 1		Trimester 2		Trimester 3	
**Exposure**	**N**	**OR (95% CI)**	**N**	**OR (95% CI)**	**N**	**OR (95% CI)**	**N**	**OR (95% CI)**
LMW	136	**3·64 (1·56, 8·5)**			91	**5·17 (1·32, 20·26)**	85	1·59 (0·63, 3·99)
HMW	141	1·13 (0·39, 3·25)			91	2·66 (0·49, 14·34)	78	0·56 (0·17, 1·85)
DEHP	141	1·06 (0·39, 2·89)	20	0·02 (0, 9·46)	91	1·98 (0·38, 10·45)	78	0·86 (0·27, 2·74)
PA	84	2·71 (0·88, 8·32)			51	5·35 (0·46, 61·59)	65	2·58 (0·8, 8·35)
DiNP	96	2·34 (0·93, 5·9)			66	2·13 (0·46, 9·85)	44	1·07 (0·4, 2·81)
DnOP	67	0·55 (0·11, 2·82)			39	0·54 (0·04, 7·42)	42	0·9 (0·21, 3·84)
DiDP	126	0·81 (0·34, 1·95)			88	1·02 (0·28, 3·77)	78	**0·3 (0·11, 0·87)**

Outcome: Low Birthweight (Terms only)

	Across Pregnancy		Trimester 1		Trimester 2		Trimester 3	
**Exposure**	**N**	**OR (95% CI)**	**N**	**OR (95% CI)**	**N**	**OR (95% CI)**	**N**	**OR (95% CI)**
LMW	1665	1·16 (0·61, 2·18)	350	1·33 (0·47, 3·75)	1160	1·31 (0·62, 2·81)	1129	1·18 (0·62, 2·27)
HMW	1669	1·58 (0·74, 3·37)	400	2·84 (0·88, 9·18)	1135	1·13 (0·45, 2·81)	1016	1·27 (0·55, 2·9)
DEHP	1671	1·55 (0·76, 3·16)	412	**3·17 (1·04, 9·61)**	1137	1·09 (0·45, 2·63)	1019	1·29 (0·6, 2·76)
PA	916	1·16 (0·43, 3·09)	315	1·85 (0·6, 5·7)	741	1·41 (0·54, 3·65)	738	0·78 (0·34, 1·78)
DiNP	1064	0·74 (0·34, 1·6)	274	0·25 (0·06, 1·08)	867	0·57 (0·26, 1·25)	460	1·18 (0·53, 2·62)
DnOP	694	0·55 (0·13, 2·35)	271	0·7 (0·19, 2·61)	594	0·34 (0·09, 1·37)	363	0·89 (0·27, 2·94)
DiDP	1447	0·57 (0·27, 1·19)	317	1·11 (0·44, 2·81)	1130	**0·49 (0·25, 0·97)**	941	0·85 (0·42, 1·73)

Supplement Table 6a2: Parity-Stratified Adjusted Associations of Phthalate Exposures with Birth Outcomes (Continuously Measured), Parity=1 Only

Models adjusted for maternal age, race/ethnicity and education, and child sex· BW for GA model removes parity from covariates·

Outcome: Gestational Age

	Across Pregnancy		Trimester 1		Trimester 2		Trimester 3	
**Exposure**	**N**	**Beta (95% CI)**	**N**	**Beta (95% CI)**	**N**	**Beta (95% CI)**	**N**	**Beta (95% CI)**
LMW	1620	-0·05 (-0·248, 0·148)	483	-0·116 (-0·407, 0·175)	1211	-0·093 (-0·296, 0·11)	1160	-0·012 (-0·198, 0·173)
HMW	1659	-0·121 (-0·359, 0·118)	557	-0·222 (-0·543, 0·098)	1192	0·152 (-0·086, 0·39)	1070	-0·122 (-0·393, 0·15)
DEHP	1661	-0·094 (-0·319, 0·13)	565	-0·241 (-0·544, 0·062)	1196	0·135 (-0·088, 0·357)	1075	-0·118 (-0·369, 0·133)
PA	1044	**-0·573 (-0·837, -0·31)**	457	-0·044 (-0·342, 0·253)	855	-0·117 (-0·339, 0·105)	860	**-0·393 (-0·601, -0·186)**
DiNP	1115	**-0·383 (-0·582, -0·184)**	435	0·047 (-0·227, 0·321)	928	-0·025 (-0·206, 0·155)	621	**-0·386 (-0·618, -0·154)**
DnOP	868	-0·237 (-0·583, 0·108)	429	0·228 (-0·076, 0·533)	725	0·014 (-0·268, 0·295)	578	-0·174 (-0·488, 0·139)
DiDP	1428	-0·11 (-0·307, 0·087)	458	0·073 (-0·177, 0·323)	1159	0·074 (-0·099, 0·246)	989	-0·162 (-0·347, 0·023)

Outcome: Birth Length

	Across Pregnancy		Trimester 1		Trimester 2		Trimester 3	
**Exposure**	**N**	**Beta (95% CI)**	**N**	**Beta (95% CI)**	**N**	**Beta (95% CI)**	**N**	**Beta (95% CI)**
LMW	1380	0·117 (-0·264, 0·499)	389	-0·447 (-0·957, 0·063)	1071	0·229 (-0·148, 0·605)	992	0·095 (-0·27, 0·46)
HMW	1438	-0·037 (-0·488, 0·413)	472	0·058 (-0·524, 0·639)	1057	0·312 (-0·119, 0·742)	909	-0·07 (-0·604, 0·465)
DEHP	1440	0·086 (-0·34, 0·512)	479	0·145 (-0·41, 0·7)	1061	0·358 (-0·047, 0·763)	914	0·07 (-0·423, 0·563)
PA	840	-0·148 (-0·702, 0·405)	371	-0·108 (-0·633, 0·418)	735	0·315 (-0·111, 0·742)	712	-0·193 (-0·622, 0·235)
DiNP	958	**-0·847 (-1·218, -0·476)**	377	0·046 (-0·425, 0·516)	820	-0·184 (-0·52, 0·152)	482	**-0·881 (-1·332, -0·43)**
DnOP	712	-0·329 (-0·982, 0·325)	371	0·211 (-0·325, 0·748)	618	-0·214 (-0·738, 0·31)	439	-0·456 (-1·075, 0·164)
DiDP	1225	**-0·455 (-0·842, -0·068)**	377	0·344 (-0·086, 0·774)	1034	0·061 (-0·266, 0·388)	842	**-0·747 (-1·106, -0·388)**

Outcome: Birthweight

	Across Pregnancy		Trimester 1		Trimester 2		Trimester 3	
**Exposure**	**N**	**Beta (95% CI)**	**N**	**Beta (95% CI)**	**N**	**Beta (95% CI)**	**N**	**Beta (95% CI)**
LMW	1620	-16·634 (-76·791, 43·523)	483	-41·979 (-135·165, 51·208)	1211	0·388 (-65·101, 65·878)	1160	-20·204 (-77·403, 36·994)
HMW	1659	26·646 (-45·522, 98·815)	557	35·5 (-66·942, 137·943)	1192	-8·499 (-85·057, 68·059)	1070	13·018 (-69·658, 95·694)
DEHP	1661	24·163 (-43·85, 92·175)	565	30·915 (-67·051, 128·88)	1196	-3·612 (-75·354, 68·13)	1075	3·064 (-73·211, 79·34)
PA	1044	**-101·196 (-182·401, -19·99)**	457	9·392 (-85·515, 104·299)	855	-16·971 (-90·044, 56·103)	860	**-79·549 (-144·515, -14·583)**
DiNP	1115	-53·494 (-114·331, 7·343)	435	58·683 (-27·496, 144·862)	928	8·649 (-50·544, 67·842)	621	-51·247 (-119·933, 17·438)
DnOP	868	-79·112 (-181·381, 23·157)	429	55·388 (-40·667, 151·443)	725	-33·196 (-123·506, 57·114)	578	-86·01 (-178·188, 6·169)
DiDP	1428	-13·33 (-74·028, 47·367)	458	68·981 (-10·325, 148·286)	1159	18·34 (-37·883, 74·564)	989	-39·669 (-97·58, 18·241)

Outcome: Birthweight for Gestational Age Z-Score

	Across Pregnancy		Trimester 1		Trimester 2		Trimester 3	
**Exposure**	**N**	**Beta (95% CI)**	**N**	**Beta (95% CI)**	**N**	**Beta (95% CI)**	**N**	**Beta (95% CI)**
LMW	1471	-0·014 (-0·142, 0·115)	360	-0·106 (-0·33, 0·117)	1073	0·041 (-0·106, 0·188)	1019	-0·035 (-0·163, 0·092)
HMW	1510	0·112 (-0·038, 0·262)	434	0·099 (-0·141, 0·339)	1054	-0·096 (-0·263, 0·071)	929	0·13 (-0·048, 0·308)
DEHP	1512	0·098 (-0·044, 0·24)	442	0·111 (-0·124, 0·346)	1058	-0·061 (-0·218, 0·096)	934	0·089 (-0·075, 0·253)
PA	897	0·054 (-0·112, 0·221)	334	0·101 (-0·132, 0·334)	717	0·038 (-0·13, 0·205)	721	0·071 (-0·067, 0·209)
DiNP	968	0·056 (-0·067, 0·178)	312	0·147 (-0·052, 0·346)	790	0·054 (-0·073, 0·181)	482	0·052 (-0·093, 0·198)
DnOP	721	-0·063 (-0·269, 0·143)	306	0·035 (-0·198, 0·268)	587	-0·107 (-0·302, 0·089)	439	-0·156 (-0·364, 0·052)
DiDP	1281	-0·008 (-0·138, 0·123)	335	0·099 (-0·082, 0·279)	1021	0 (-0·131, 0·131)	850	-0·076 (-0·2, 0·048)

Supplement Table 6b2: Parity-Stratified Adjusted Associations of Phthalate Exposures with Categorical Birth Outcomes, Parity=1 Only

Models adjusted for maternal age, race/ethnicity and education, and child sex·

Outcome: Preterm

	Across Pregnancy		Trimester 1		Trimester 2		Trimester 3	
**Exposure**	**N**	**OR (95% CI)**	**N**	**OR (95% CI)**	**N**	**OR (95% CI)**	**N**	**OR (95% CI)**
LMW	1620	1·13 (0·74, 1·74)	483	1·17 (0·54, 2·53)	1211	1·31 (0·8, 2·17)	1160	1·06 (0·69, 1·63)
HMW	1659	1·24 (0·73, 2·09)	557	1·26 (0·52, 3·05)	1192	0·66 (0·35, 1·24)	1070	1·38 (0·77, 2·47)
DEHP	1661	1·04 (0·63, 1·7)	565	1·07 (0·46, 2·49)	1196	0·59 (0·32, 1·08)	1075	1·28 (0·75, 2·19)
PA	1044	**2·04 (1·2, 3·47)**	457	1·2 (0·52, 2·81)	855	1·35 (0·76, 2·4)	860	**1·91 (1·14, 3·21)**
DiNP	1115	**1·62 (1·07, 2·46)**	435	1 (0·49, 2·06)	928	0·82 (0·5, 1·35)	621	**1·82 (1·15, 2·88)**
DnOP	868	1·67 (0·88, 3·18)	429	0·51 (0·22, 1·21)	725	1·15 (0·59, 2·25)	578	1·52 (0·85, 2·72)
DiDP	1428	1 (0·64, 1·57)	458	0·75 (0·39, 1·45)	1159	**0·64 (0·41, 1)**	989	1·46 (0·94, 2·27)

Outcome: Small for Gestational Age

	Across Pregnancy		Trimester 1		Trimester 2		Trimester 3	
**Exposure**	**N**	**OR (95% CI)**	**N**	**OR (95% CI)**	**N**	**OR (95% CI)**	**N**	**OR (95% CI)**
LMW	1618	1·02 (0·7, 1·48)	483	1·14 (0·64, 2·05)	1211	0·9 (0·59, 1·38)	1158	0·89 (0·62, 1·29)
HMW	1657	1·1 (0·71, 1·72)	557	0·86 (0·43, 1·7)	1192	1·52 (0·96, 2·42)	1068	1·06 (0·64, 1·74)
DEHP	1659	0·98 (0·64, 1·49)	565	0·83 (0·43, 1·59)	1196	1·26 (0·81, 1·95)	1073	0·94 (0·59, 1·5)
PA	1044	0·83 (0·51, 1·36)	457	0·96 (0·52, 1·76)	855	0·9 (0·58, 1·42)	860	0·81 (0·54, 1·21)
DiNP	1115	1·16 (0·8, 1·69)	435	0·76 (0·44, 1·32)	928	1 (0·69, 1·47)	621	1·12 (0·75, 1·68)
DnOP	868	1·2 (0·66, 2·18)	429	0·74 (0·4, 1·36)	725	1·11 (0·65, 1·91)	578	1·63 (0·96, 2·75)
DiDP	1428	1·01 (0·69, 1·47)	458	1·06 (0·65, 1·73)	1159	0·96 (0·67, 1·38)	989	1·11 (0·77, 1·6)

Outcome: Large for Gestational Age

	Across Pregnancy		Trimester 1		Trimester 2		Trimester 3	
**Exposure**	**N**	**OR (95% CI)**	**N**	**OR (95% CI)**	**N**	**OR (95% CI)**	**N**	**OR (95% CI)**
LMW	1618	1·04 (0·73, 1·48)	483	0·91 (0·51, 1·62)	1211	0·88 (0·59, 1·31)	1158	1·16 (0·83, 1·62)
HMW	1657	1·14 (0·74, 1·75)	557	1·3 (0·69, 2·44)	1192	0·63 (0·39, 1·04)	1068	1·09 (0·66, 1·8)
DEHP	1659	1·09 (0·72, 1·64)	565	1·33 (0·73, 2·4)	1196	0·67 (0·42, 1·06)	1073	1 (0·63, 1·61)
PA	1044	1·28 (0·83, 1·99)	457	0·95 (0·55, 1·65)	855	1·14 (0·74, 1·75)	860	1·16 (0·8, 1·68)
DiNP	1115	1·29 (0·92, 1·81)	435	1·41 (0·84, 2·35)	928	1·1 (0·78, 1·56)	621	1·18 (0·8, 1·72)
DnOP	868	1·04 (0·58, 1·87)	429	0·88 (0·48, 1·61)	725	0·7 (0·39, 1·24)	578	1·11 (0·67, 1·82)
DiDP	1428	1·26 (0·89, 1·78)	458	1·48 (0·9, 2·42)	1159	1·01 (0·72, 1·41)	989	1·33 (0·94, 1·88)

Outcome: Low Birthweight

	Across Pregnancy		Trimester 1		Trimester 2		Trimester 3	
**Exposure**	**N**	**OR (95% CI)**	**N**	**OR (95% CI)**	**N**	**OR (95% CI)**	**N**	**OR (95% CI)**
LMW	1620	0·99 (0·59, 1·64)	483	1·09 (0·49, 2·45)	1211	0·84 (0·46, 1·53)	1160	0·96 (0·58, 1·6)
HMW	1659	0·97 (0·53, 1·79)	557	1·41 (0·58, 3·43)	1192	0·74 (0·37, 1·48)	1070	0·85 (0·43, 1·71)
DEHP	1661	0·92 (0·52, 1·62)	565	1·48 (0·65, 3·34)	1196	0·77 (0·4, 1·47)	1075	0·96 (0·51, 1·81)
PA	1044	**1·99 (1·12, 3·54)**	457	1·16 (0·46, 2·91)	855	1·17 (0·62, 2·19)	860	1·73 (0·99, 3·01)
DiNP	1115	**1·95 (1·25, 3·05)**	435	1·41 (0·69, 2·88)	928	1·21 (0·73, 2)	621	1·64 (0·99, 2·72)
DnOP	868	**2 (1·02, 3·92)**	429	0·75 (0·31, 1·8)	725	1·32 (0·66, 2·62)	578	**2·04 (1·09, 3·8)**
DiDP	1428	1·61 (0·98, 2·63)	458	1·29 (0·66, 2·52)	1159	1·08 (0·66, 1·78)	989	**1·64 (1·01, 2·65)**

Outcome: Low Birthweight (Preterms only)

	Across Pregnancy		Trimester 1		Trimester 2		Trimester 3	
**Exposure**	**N**	**OR (95% CI)**	**N**	**OR (95% CI)**	**N**	**OR (95% CI)**	**N**	**OR (95% CI)**
LMW	122	0·83 (0·36, 1·94)	29	1·62 (0·31, 8·29)	80	0·91 (0·34, 2·42)	81	0·67 (0·28, 1·64)
HMW	119	0·84 (0·29, 2·41)	27	7·25 (0, 15381·73)	77	1·42 (0·4, 4·99)	80	0·78 (0·24, 2·49)
DEHP	120	0·74 (0·28, 1·95)	29	0·16 (0·01, 4·12)	78	1·34 (0·41, 4·32)	80	0·93 (0·32, 2·7)
PA	79	1·13 (0·41, 3·17)	26	1·2 (0·1, 14·51)	55	0·7 (0·18, 2·71)	55	1·18 (0·42, 3·33)
DiNP	81	1·81 (0·83, 3·93)			57	3·08 (0·92, 10·35)	48	1·2 (0·52, 2·75)
DnOP	69	1·42 (0·36, 5·54)			49	3·32 (0·44, 24·93)	44	0·71 (0·15, 3·34)
DiDP	99	1·69 (0·77, 3·7)	26	2·54 (0·23, 27·73)	73	1·97 (0·75, 5·2)	62	0·93 (0·38, 2·28)

Outcome: Low Birthweight (Terms only)

	Across Pregnancy		Trimester 1		Trimester 2		Trimester 3	
**Exposure**	**N**	**OR (95% CI)**	**N**	**OR (95% CI)**	**N**	**OR (95% CI)**	**N**	**OR (95% CI)**
LMW	1498	0·97 (0·41, 2·32)	454	1·13 (0·38, 3·33)	1131	0·5 (0·18, 1·38)	1079	1·24 (0·55, 2·8)
HMW	1540	0·8 (0·29, 2·21)	530	2·06 (0·7, 6·04)	1115	0·83 (0·28, 2·46)	990	0·5 (0·16, 1·61)
DEHP	1541	0·97 (0·39, 2·42)	536	2·57 (0·98, 6·74)	1118	0·99 (0·37, 2·68)	995	0·65 (0·23, 1·83)
PA	965	1·48 (0·52, 4·21)	431	1·25 (0·36, 4·35)	800	1·29 (0·48, 3·44)	805	1·03 (0·4, 2·61)
DiNP	1034	1·44 (0·67, 3·09)	410	1·17 (0·44, 3·07)	871	1·27 (0·61, 2·64)	573	0·95 (0·39, 2·3)
DnOP	799	2·1 (0·72, 6·11)	404	0·52 (0·16, 1·67)	676	1·47 (0·54, 4)	534	**2·87 (1·06, 7·78)**
DiDP	1329	1·96 (0·84, 4·58)	432	1·37 (0·55, 3·46)	1086	1·33 (0·6, 2·95)	927	2·02 (0·94, 4·37)

Supplement Table 6a3: Parity-Stratified Adjusted Associations of Phthalate Exposures with Birth Outcomes (Continuously Measured), Parity=2+ Only

Models adjusted for maternal age, race/ethnicity and education, and child sex· BW for GA model removes parity from covariates·

Outcome: Gestational Age

	Across Pregnancy		Trimester 1		Trimester 2		Trimester 3	
**Exposure**	**N**	**Beta (95% CI)**	**N**	**Beta (95% CI)**	**N**	**Beta (95% CI)**	**N**	**Beta (95% CI)**
LMW	968	-0·028 (-0·291, 0·234)	152	0·185 (-0·423, 0·794)	662	0·149 (-0·138, 0·437)	723	0·118 (-0·136, 0·372)
HMW	977	-0·251 (-0·552, 0·051)	193	-0·258 (-0·911, 0·396)	644	0·033 (-0·298, 0·364)	667	-0·205 (-0·545, 0·136)
DEHP	981	**-0·37 (-0·653, -0·086)**	200	-0·396 (-1·034, 0·241)	645	-0·084 (-0·395, 0·226)	672	**-0·323 (-0·633, -0·013)**
PA	563	**-0·762 (-1·15, -0·373)**	125	-0·409 (-1·156, 0·337)	432	-0·023 (-0·397, 0·351)	480	**-0·507 (-0·826, -0·189)**
DiNP	570	**-0·56 (-0·872, -0·248)**	115	-0·209 (-0·696, 0·278)	449	0·103 (-0·194, 0·4)	275	**-0·723 (-1·087, -0·358)**
DnOP	408	**-1·339 (-1·922, -0·757)**	107	-0·341 (-0·954, 0·272)	316	-0·181 (-0·714, 0·352)	234	**-1·097 (-1·659, -0·535)**
DiDP	774	**-0·652 (-0·976, -0·328)**	132	-0·347 (-0·921, 0·227)	593	-0·061 (-0·382, 0·26)	560	**-0·528 (-0·821, -0·234)**

Outcome: Birth Length

	Across Pregnancy		Trimester 1		Trimester 2		Trimester 3	
**Exposure**	**N**	**Beta (95% CI)**	**N**	**Beta (95% CI)**	**N**	**Beta (95% CI)**	**N**	**Beta (95% CI)**
LMW	775	-0·247 (-0·734, 0·241)	91	-0·554 (-1·759, 0·651)	544	0·225 (-0·306, 0·756)	590	-0·041 (-0·525, 0·444)
HMW	812	-0·297 (-0·832, 0·239)	141	-0·093 (-1·364, 1·179)	537	0·444 (-0·149, 1·037)	544	-0·344 (-0·956, 0·268)
DEHP	816	-0·401 (-0·906, 0·103)	148	-0·471 (-1·685, 0·743)	538	0·332 (-0·214, 0·878)	549	-0·442 (-1·001, 0·117)
PA	403	**-1·725 (-2·525, -0·925)**	70	0·418 (-1·073, 1·909)	335	-0·03 (-0·773, 0·713)	369	**-1·402 (-2·11, -0·695)**
DiNP	459	**-0·974 (-1·555, -0·393)**	79	-0·189 (-1·051, 0·673)	374	0·143 (-0·381, 0·667)	175	**-1·451 (-2·251, -0·651)**
DnOP	296	**-1·401 (-2·617, -0·185)**	70	0·278 (-0·947, 1·504)	242	0·603 (-0·373, 1·578)	134	**-1·464 (-2·863, -0·065)**
DiDP	621	**-0·78 (-1·413, -0·146)**	79	-0·196 (-1·335, 0·942)	498	0·515 (-0·089, 1·12)	451	**-0·857 (-1·404, -0·309)**

Outcome: Birthweight

	Across Pregnancy		Trimester 1		Trimester 2		Trimester 3	
**Exposure**	**N**	**Beta (95% CI)**	**N**	**Beta (95% CI)**	**N**	**Beta (95% CI)**	**N**	**Beta (95% CI)**
LMW	968	-39·635 (-119·279, 40·009)	152	71·882 (-109·866, 253·63)	662	-17·65 (-107·542, 72·241)	723	21·415 (-58·168, 100·999)
HMW	977	-23·156 (-114·985, 68·672)	193	136·724 (-66·128, 339·575)	644	15·135 (-88·936, 119·206)	667	-0·621 (-106·095, 104·853)
DEHP	981	-66·838 (-153·31, 19·634)	200	35·859 (-156·311, 228·029)	645	-13·096 (-110·815, 84·623)	672	-54·71 (-150·863, 41·444)
PA	563	**-186·205 (-303·654, -68·757)**	125	-120·873 (-355·675, 113·928)	432	-13·271 (-126·104, 99·562)	480	-93·503 (-194·85, 7·844)
DiNP	570	**-129·973 (-225·49, -34·457)**	115	57·555 (-95·19, 210·301)	449	44·561 (-45·929, 135·051)	275	**-191·853 (-305·444, -78·261)**
DnOP	408	**-221·272 (-394·528, -48·017)**	107	-71·02 (-262·577, 120·537)	316	50·752 (-103·899, 205·403)	234	**-208·226 (-382·172, -34·28)**
DiDP	774	-87·38 (-188·612, 13·851)	132	112·485 (-67·79, 292·76)	593	76·28 (-23·591, 176·151)	560	**-122·245 (-217·138, -27·351)**

Outcome: Birthweight for Gestational Age Z-Score

	Across Pregnancy		Trimester 1		Trimester 2		Trimester 3	
**Exposure**	**N**	**Beta (95% CI)**	**N**	**Beta (95% CI)**	**N**	**Beta (95% CI)**	**N**	**Beta (95% CI)**
LMW	893	-0·089 (-0·252, 0·074)	87	-0·057 (-0·511, 0·398)	594	-0·133 (-0·334, 0·068)	651	0·003 (-0·167, 0·173)
HMW	902	0·068 (-0·124, 0·259)	128	0·369 (-0·122, 0·861)	576	-0·041 (-0·269, 0·186)	595	0·123 (-0·103, 0·349)
DEHP	906	0·026 (-0·155, 0·207)	135	0·305 (-0·162, 0·773)	577	-0·021 (-0·238, 0·195)	600	0·031 (-0·176, 0·238)
PA	491	-0·133 (-0·36, 0·094)	61	0·03 (-0·591, 0·651)	366	-0·058 (-0·292, 0·176)	410	-0·04 (-0·245, 0·165)
DiNP	496	-0·076 (-0·275, 0·124)	50	0·265 (-0·146, 0·675)	381	0·002 (-0·205, 0·209)	204	-0·139 (-0·394, 0·117)
DnOP	336	0·017 (-0·334, 0·369)	43	0·046 (-0·652, 0·744)	250	0·087 (-0·274, 0·449)	164	0·06 (-0·33, 0·45)
DiDP	700	0·058 (-0·155, 0·271)	67	**0·575 (0·087, 1·064)**	525	0·143 (-0·098, 0·384)	489	-0·058 (-0·258, 0·141)

Supplement Table 6b3: Parity-Stratified Adjusted Associations of Phthalate Exposures with Categorical Birth Outcomes, Parity=2+ Only

Models adjusted for maternal age, race/ethnicity and education, and child sex·

Outcome: Preterm

	Across Pregnancy		Trimester 1		Trimester 2		Trimester 3	
**Exposure**	**N**	**OR (95% CI)**	**N**	**OR (95% CI)**	**N**	**OR (95% CI)**	**N**	**OR (95% CI)**
LMW	968	0·82 (0·48, 1·4)	152	0·38 (0·1, 1·47)	662	0·58 (0·28, 1·2)	723	0·79 (0·46, 1·34)
HMW	977	1·56 (0·86, 2·82)	193	1·05 (0·19, 5·66)	644	0·58 (0·27, 1·26)	667	1·83 (0·96, 3·5)
DEHP	981	**1·74 (1·01, 3)**	200	1·64 (0·36, 7·46)	645	0·79 (0·38, 1·62)	672	**1·91 (1·08, 3·37)**
PA	563	**2·48 (1·32, 4·68)**	125	1·44 (0·32, 6·56)	432	0·75 (0·36, 1·57)	480	**2·05 (1·1, 3·8)**
DiNP	570	**1·83 (1·07, 3·14)**	115	0·54 (0·15, 1·97)	449	**0·45 (0·23, 0·91)**	275	**1·98 (1·16, 3·38)**
DnOP	408	**4·25 (1·95, 9·23)**	107	1·18 (0·29, 4·71)	316	1·08 (0·44, 2·64)	234	**3·88 (1·85, 8·17)**
DiDP	774	1·56 (0·82, 2·95)	132	0·66 (0·16, 2·67)	593	0·56 (0·28, 1·13)	560	1·71 (0·96, 3·06)

Outcome: Small for Gestational Age

	Across Pregnancy		Trimester 1		Trimester 2		Trimester 3	
**Exposure**	**N**	**OR (95% CI)**	**N**	**OR (95% CI)**	**N**	**OR (95% CI)**	**N**	**OR (95% CI)**
LMW	967	0·72 (0·4, 1·27)	152	0·55 (0·12, 2·49)	662	0·74 (0·38, 1·45)	722	0·64 (0·35, 1·15)
HMW	976	0·53 (0·27, 1·02)	193	**0·2 (0·04, 0·99)**	644	0·86 (0·41, 1·8)	666	0·45 (0·2, 1·02)
DEHP	980	0·74 (0·4, 1·36)	200	0·31 (0·07, 1·35)	645	0·99 (0·5, 1·97)	671	0·68 (0·34, 1·37)
PA	563	1·02 (0·47, 2·2)	125	0·84 (0·13, 5·4)	432	0·86 (0·38, 1·92)	480	1·02 (0·49, 2·09)
DiNP	570	1·02 (0·54, 1·91)	115	0·86 (0·28, 2·61)	449	0·81 (0·43, 1·52)	275	1·16 (0·59, 2·29)
DnOP	408	1·2 (0·44, 3·3)	107	0·92 (0·23, 3·73)	316	0·74 (0·25, 2·14)	234	0·92 (0·35, 2·38)
DiDP	774	1·35 (0·69, 2·65)	132	0·71 (0·18, 2·83)	593	0·74 (0·37, 1·49)	560	1·55 (0·82, 2·93)

Outcome: Large for Gestational Age

	Across Pregnancy		Trimester 1		Trimester 2		Trimester 3	
**Exposure**	**N**	**OR (95% CI)**	**N**	**OR (95% CI)**	**N**	**OR (95% CI)**	**N**	**OR (95% CI)**
LMW	967	**0·61 (0·38, 0·99)**	152	1·14 (0·4, 3·21)	662	0·63 (0·36, 1·11)	722	0·66 (0·42, 1·06)
HMW	976	1·09 (0·65, 1·82)	193	1·5 (0·45, 4·99)	644	1·02 (0·56, 1·85)	666	1·12 (0·62, 2·03)
DEHP	980	0·94 (0·57, 1·54)	200	1·32 (0·4, 4·29)	645	0·84 (0·48, 1·48)	671	0·96 (0·55, 1·68)
PA	563	1 (0·56, 1·77)	125	0·84 (0·28, 2·56)	432	1·39 (0·74, 2·61)	480	0·96 (0·59, 1·55)
DiNP	570	0·88 (0·54, 1·44)	115	1·18 (0·54, 2·54)	449	1·01 (0·6, 1·7)	275	0·59 (0·34, 1·01)
DnOP	408	1·43 (0·64, 3·21)	107	0·64 (0·24, 1·72)	316	1·31 (0·58, 2·99)	234	0·79 (0·38, 1·66)
DiDP	774	1·2 (0·69, 2·11)	132	2·01 (0·82, 4·92)	593	1·12 (0·62, 2·02)	560	0·86 (0·51, 1·45)

Outcome: Low Birthweight

	Across Pregnancy		Trimester 1		Trimester 2		Trimester 3	
**Exposure**	**N**	**OR (95% CI)**	**N**	**OR (95% CI)**	**N**	**OR (95% CI)**	**N**	**OR (95% CI)**
LMW	968	0·83 (0·42, 1·63)	152	1·09 (0·49, 2·45)	662	0·71 (0·3, 1·67)	723	0·54 (0·27, 1·08)
HMW	977	1·24 (0·57, 2·69)	193	1·41 (0·58, 3·43)	644	0·78 (0·3, 2·03)	667	1·01 (0·43, 2·35)
DEHP	981	1·5 (0·75, 3·01)	200	**0·02 (0, 0·46)**	645	0·97 (0·4, 2·34)	672	1·3 (0·63, 2·7)
PA	563	**2·65 (1·24, 5·66)**	125	1·16 (0·46, 2·91)	432	0·97 (0·38, 2·48)	480	1·77 (0·84, 3·71)
DiNP	570	**2·17 (1·16, 4·04)**	115	1·41 (0·69, 2·88)	449	0·5 (0·22, 1·16)	275	**1·86 (1·01, 3·43)**
DnOP	408	1·64 (0·67, 4)	107	0·75 (0·31, 1·8)	316	**0·2 (0·05, 0·75)**	234	1·56 (0·69, 3·55)
DiDP	774	**2·46 (1·15, 5·24)**	132	1·29 (0·66, 2·52)	593	**0·39 (0·16, 0·91)**	560	**3·01 (1·53, 5·92)**

Outcome: Low Birthweight (Preterms only)

	Across Pregnancy		Trimester 1		Trimester 2		Trimester 3	
**Exposure**	**N**	**OR (95% CI)**	**N**	**OR (95% CI)**	**N**	**OR (95% CI)**	**N**	**OR (95% CI)**
LMW	87	1·03 (0·39, 2·72)	17	0·01 (0, 6·31)	48	1·62 (0·24, 11·07)	64	0·87 (0·31, 2·44)
HMW	84	1·49 (0·37, 5·91)			48	0·76 (0·13, 4·43)	59	1·75 (0·41, 7·43)
DEHP	85	1·87 (0·5, 7·07)			48	0·86 (0·16, 4·66)	60	1·74 (0·45, 6·74)
PA	63	1·44 (0·53, 3·88)			38	1·48 (0·15, 14·54)	49	2·05 (0·73, 5·74)
DiNP	56	1·94 (0·74, 5·14)					36	1·78 (0·66, 4·81)
DnOP	55	0·7 (0·2, 2·4)					37	1·18 (0·34, 4·07)
DiDP	70	2·2 (0·72, 6·72)			44	0·48 (0·1, 2·36)	51	**5·47 (1·34, 22·39)**

Outcome: Low Birthweight (Terms only)

	Across Pregnancy		Trimester 1		Trimester 2		Trimester 3	
**Exposure**	**N**	**OR (95% CI)**	**N**	**OR (95% CI)**	**N**	**OR (95% CI)**	**N**	**OR (95% CI)**
LMW	881	0·99 (0·38, 2·58)			614	1 (0·35, 2·91)	659	0·44 (0·17, 1·16)
HMW	893	0·78 (0·26, 2·34)			596	1·35 (0·37, 4·94)	608	0·48 (0·14, 1·66)
DEHP	896	1·02 (0·38, 2·75)			597	1·57 (0·5, 4·98)	612	0·76 (0·26, 2·2)
PA	500	1·62 (0·44, 5·99)			394	1·53 (0·41, 5·66)	431	0·54 (0·18, 1·59)
DiNP	514	1·4 (0·51, 3·8)			413	0·91 (0·29, 2·83)	239	1·17 (0·45, 3·09)
DnOP	353	0·58 (0·09, 3·72)			282	0·23 (0·04, 1·55)	197	0·39 (0·07, 2·04)
DiDP	704	1·74 (0·56, 5·4)			549	0·5 (0·14, 1·75)	509	1·66 (0·65, 4·26)

Supplement Table 7a: Sensitivity Analysis for Adjusted Associations of Phthalate Exposures with Birth Outcomes (Continuously Measured), Adding Tobacco as a Covariate

Models adjusted for maternal age, race/ethnicity, parity, tobacco use, and education, and child sex· BW for GA model removes parity from covariates·

Outcome: Gestational Age

	Across Pregnancy		Trimester 1		Trimester 2		Trimester 3	
**Exposure**	**N**	**Beta (95% CI)**	**N**	**Beta (95% CI)**	**N**	**Beta (95% CI)**	**N**	**Beta (95% CI)**
LMW	3927	-0·021 (-0·152, 0·11)	709	-0·003 (-0·254, 0·248)	2796	0·035 (-0·106, 0·175)	2810	-0·051 (-0·179, 0·077)
HMW	3790	-0·031 (-0·197, 0·134)	670	-0·205 (-0·524, 0·114)	2739	0·129 (-0·032, 0·291)	2553	-0·141 (-0·315, 0·033)
DEHP	3798	-0·029 (-0·186, 0·127)	699	-0·149 (-0·459, 0·161)	2746	0·118 (-0·036, 0·272)	2566	-0·141 (-0·302, 0·02)
PA	2246	**-0·699 (-0·9, -0·497)**	638	0·137 (-0·117, 0·391)	1827	-0·083 (-0·256, 0·089)	1916	**-0·528 (-0·683, -0·373)**
DiNP	2528	**-0·502 (-0·642, -0·361)**	624	0·046 (-0·182, 0·274)	2028	-0·033 (-0·165, 0·098)	1129	**-0·638 (-0·818, -0·459)**
DnOP	1805	**-0·787 (-1·057, -0·517)**	624	0·093 (-0·169, 0·355)	1461	-0·069 (-0·296, 0·159)	1000	**-0·724 (-1, -0·449)**
DiDP	3344	**-0·36 (-0·514, -0·207)**	638	0·127 (-0·083, 0·337)	2654	-0·023 (-0·161, 0·115)	2291	**-0·282 (-0·416, -0·148)**

Outcome: Birth Length

	Across Pregnancy		Trimester 1		Trimester 2		Trimester 3	
**Exposure**	**N**	**Beta (95% CI)**	**N**	**Beta (95% CI)**	**N**	**Beta (95% CI)**	**N**	**Beta (95% CI)**
LMW	3466	-0·06 (-0·29, 0·17)	595	-0·286 (-0·724, 0·152)	2526	0·08 (-0·165, 0·324)	2446	-0·085 (-0·317, 0·147)
HMW	3352	0·042 (-0·246, 0·33)	569	-0·004 (-0·541, 0·534)	2474	0·231 (-0·048, 0·509)	2206	-0·049 (-0·361, 0·264)
DEHP	3360	0·129 (-0·145, 0·402)	594	0·103 (-0·415, 0·621)	2481	**0·293 (0·028, 0·558)**	2219	0·005 (-0·284, 0·295)
PA	1891	**-0·895 (-1·276, -0·515)**	551	0·016 (-0·429, 0·461)	1614	0·138 (-0·172, 0·449)	1615	**-0·764 (-1·075, -0·453)**
DiNP	2205	**-0·903 (-1·15, -0·656)**	551	-0·005 (-0·417, 0·406)	1823	-0·104 (-0·334, 0·127)	862	**-1·086 (-1·431, -0·741)**
DnOP	1490	**-0·829 (-1·325, -0·332)**	551	0·179 (-0·265, 0·623)	1264	0·132 (-0·271, 0·535)	731	**-0·986 (-1·538, -0·435)**
DiDP	2973	**-0·713 (-0·984, -0·443)**	552	0·083 (-0·271, 0·437)	2425	0·006 (-0·233, 0·245)	1988	**-0·762 (-1·003, -0·521)**

Outcome: Birthweight

	Across Pregnancy		Trimester 1		Trimester 2		Trimester 3	
**Exposure**	**N**	**Beta (95% CI)**	**N**	**Beta (95% CI)**	**N**	**Beta (95% CI)**	**N**	**Beta (95% CI)**
LMW	3927	-22·591 (-60·099, 14·916)	709	-4·689 (-80·035, 70·658)	2796	-18·354 (-60·143, 23·434)	2810	-16·361 (-54·268, 21·547)
HMW	3790	16·394 (-30·883, 63·67)	670	43·001 (-51·93, 137·931)	2739	9·802 (-38·237, 57·842)	2553	8·502 (-42·628, 59·633)
DEHP	3798	10·955 (-33·862, 55·772)	699	45·742 (-46·817, 138·301)	2746	12·012 (-33·788, 57·813)	2566	-6·628 (-53·979, 40·724)
PA	2246	**-141·221 (-198·178, -84·264)**	638	53·496 (-23·367, 130·359)	1827	-7·451 (-58·274, 43·371)	1916	**-101·899 (-148·23, -55·568)**
DiNP	2528	**-94·215 (-134·106, -54·324)**	624	**78·621 (10·155, 147·088)**	2028	16·967 (-21·921, 55·856)	1129	**-127·944 (-178·622, -77·267)**
DnOP	1805	**-165·293 (-239·206, -91·381)**	624	46·597 (-32·506, 125·7)	1461	-15·738 (-81·067, 49·59)	1000	**-171·741 (-248·707, -94·776)**
DiDP	3344	**-83·27 (-127·645, -38·896)**	638	34·158 (-29·526, 97·842)	2654	-1·856 (-43·209, 39·497)	2291	**-75·887 (-116·017, -35·757)**

Outcome: Birthweight for Gestational Age Z-Score

	Across Pregnancy		Trimester 1		Trimester 2		Trimester 3	
**Exposure**	**N**	**Beta (95% CI)**	**N**	**Beta (95% CI)**	**N**	**Beta (95% CI)**	**N**	**Beta (95% CI)**
LMW	3914	-0·034 (-0·11, 0·043)	704	-0·048 (-0·208, 0·112)	2789	-0·051 (-0·138, 0·037)	2799	-0·009 (-0·087, 0·069)
HMW	3777	0·078 (-0·017, 0·174)	665	0·185 (-0·017, 0·386)	2732	-0·033 (-0·133, 0·067)	2542	**0·12 (0·016, 0·223)**
DEHP	3785	0·061 (-0·03, 0·152)	694	0·153 (-0·045, 0·352)	2739	-0·024 (-0·12, 0·071)	2555	0·08 (-0·017, 0·177)
PA	2241	-0·009 (-0·115, 0·097)	634	0·035 (-0·125, 0·195)	1822	0·016 (-0·086, 0·117)	1911	0·004 (-0·086, 0·095)
DiNP	2523	0·022 (-0·055, 0·098)	620	**0·179 (0·035, 0·324)**	2023	0·061 (-0·018, 0·14)	1124	0·007 (-0·087, 0·1)
DnOP	1800	-0·028 (-0·162, 0·106)	620	0·004 (-0·163, 0·171)	1456	0·006 (-0·122, 0·134)	995	-0·073 (-0·212, 0·065)
DiDP	3338	-0·029 (-0·117, 0·059)	634	-0·006 (-0·14, 0·127)	2648	0·024 (-0·062, 0·11)	2286	-0·045 (-0·124, 0·034)

Supplement Table 7b: Sensitivity Analysis for Adjusted Associations of Phthalate Exposures with Categorical Birth Outcomes, Adding Tobacco as a Covariate

Models adjusted for maternal age, race/ethnicity, parity, tobacco use, and education, and child sex·

Outcome: Preterm

	Across Pregnancy		Trimester 1		Trimester 2		Trimester 3	
Exposure	N	OR (95% CI)	N	OR (95% CI)	N	OR (95% CI)	N	OR (95% CI)
LMW	3927	0·93 (0·71, 1·22)	709	0·75 (0·39, 1·43)	2796	0·82 (0·58, 1·16)	2810	1·01 (0·77, 1·35)
HMW	3790	1·29 (0·93, 1·78)	670	0·71 (0·29, 1·75)	2739	0·71 (0·48, 1·05)	2553	**1·79 (1·26, 2·54)**
DEHP	3798	1·18 (0·87, 1·61)	699	0·58 (0·25, 1·31)	2746	**0·68 (0·47, 1)**	2566	**1·65 (1·2, 2·26)**
PA	2246	**2·86 (1·99, 4·1)**	638	0·74 (0·38, 1·45)	1827	1·03 (0·67, 1·58)	1916	**2·62 (1·83, 3·74)**
DiNP	2528	**2·43 (1·88, 3·13)**	624	0·92 (0·49, 1·72)	2028	1·11 (0·82, 1·51)	1129	**2·85 (2·08, 3·91)**
DnOP	1805	**4·32 (2·83, 6·61)**	624	0·74 (0·35, 1·56)	1461	**1·7 (1·06, 2·72)**	1000	**3·81 (2·47, 5·89)**
DiDP	3344	**2·1 (1·55, 2·85)**	638	0·59 (0·32, 1·08)	2654	1·23 (0·88, 1·71)	2291	**1·97 (1·48, 2·63)**

Outcome: Small for Gestational Age

	Across Pregnancy		Trimester 1		Trimester 2		Trimester 3	
Exposure	N	OR (95% CI)	N	OR (95% CI)	N	OR (95% CI)	N	OR (95% CI)
LMW	3920	0·98 (0·77, 1·23)	708	1·1 (0·69, 1·73)	2795	0·95 (0·72, 1·25)	2804	0·87 (0·69, 1·1)
HMW	3783	1·01 (0·76, 1·36)	669	0·97 (0·54, 1·74)	2738	1·19 (0·88, 1·61)	2547	0·95 (0·7, 1·29)
DEHP	3791	1·04 (0·79, 1·37)	698	0·87 (0·5, 1·52)	2745	1·15 (0·86, 1·53)	2560	0·97 (0·73, 1·29)
PA	2246	1·14 (0·82, 1·6)	638	1·36 (0·83, 2·23)	1827	1·05 (0·76, 1·45)	1916	0·97 (0·72, 1·3)
DiNP	2528	1·01 (0·79, 1·3)	624	0·73 (0·47, 1·14)	2028	0·93 (0·72, 1·2)	1129	1·07 (0·8, 1·43)
DnOP	1805	1·09 (0·71, 1·66)	624	0·96 (0·59, 1·56)	1461	0·96 (0·64, 1·43)	1000	1·23 (0·81, 1·88)
DiDP	3344	1·14 (0·87, 1·49)	638	1·22 (0·84, 1·79)	2654	0·96 (0·74, 1·25)	2291	1·04 (0·82, 1·32)

Outcome: Large for Gestational Age

	Across Pregnancy		Trimester 1		Trimester 2		Trimester 3	
**Exposure**	**N**	**OR (95% CI)**	**N**	**OR (95% CI)**	**N**	**OR (95% CI)**	**N**	**OR (95% CI)**
LMW	3920	1·02 (0·8, 1·3)	708	1·29 (0·74, 2·25)	2795	0·87 (0·65, 1·16)	2804	1·12 (0·87, 1·44)
HMW	3783	1·12 (0·83, 1·52)	669	1·75 (0·91, 3·37)	2738	0·82 (0·59, 1·14)	2547	1·23 (0·88, 1·72)
DEHP	3791	1·09 (0·81, 1·45)	698	1·78 (0·95, 3·35)	2745	0·83 (0·61, 1·14)	2560	1·12 (0·82, 1·54)
PA	2246	1·31 (0·92, 1·85)	638	**1·97 (1·1, 3·53)**	1827	**1·43 (1, 2·04)**	1916	1·08 (0·8, 1·44)
DiNP	2528	1·11 (0·87, 1·43)	624	**1·91 (1·23, 2·98)**	2028	1·23 (0·95, 1·59)	1129	0·92 (0·66, 1·28)
DnOP	1805	1·11 (0·7, 1·75)	624	1·37 (0·79, 2·37)	1461	1 (0·64, 1·58)	1000	0·87 (0·55, 1·38)
DiDP	3344	1·1 (0·82, 1·47)	638	1·54 (0·97, 2·42)	2654	1·18 (0·89, 1·56)	2291	0·94 (0·71, 1·24)

Outcome: Low Birthweight

	Across Pregnancy		Trimester 1		Trimester 2		Trimester 3	
**Exposure**	**N**	**OR (95% CI)**	**N**	**OR (95% CI)**	**N**	**OR (95% CI)**	**N**	**OR (95% CI)**
LMW	3927	1·2 (0·89, 1·61)	709	1·13 (0·61, 2·1)	2796	1·1 (0·76, 1·58)	2810	1·05 (0·77, 1·42)
HMW	3790	1·23 (0·85, 1·78)	670	1·24 (0·57, 2·72)	2739	0·94 (0·61, 1·44)	2553	1·13 (0·76, 1·69)
DEHP	3798	1·19 (0·84, 1·69)	699	1·11 (0·53, 2·32)	2746	0·92 (0·61, 1·38)	2566	1·22 (0·85, 1·75)
PA	2246	**2·81 (1·91, 4·12)**	638	1·26 (0·63, 2·49)	1827	1·21 (0·78, 1·9)	1916	**2·03 (1·39, 2·95)**
DiNP	2528	**2·13 (1·6, 2·83)**	624	0·78 (0·42, 1·44)	2028	0·95 (0·67, 1·35)	1129	**2·18 (1·57, 3·03)**
DnOP	1805	**2·61 (1·67, 4·08)**	624	0·77 (0·38, 1·55)	1461	0·99 (0·59, 1·65)	1000	**2·52 (1·61, 3·94)**
DiDP	3344	**1·94 (1·39, 2·71)**	638	0·86 (0·5, 1·47)	2654	0·98 (0·68, 1·41)	2291	**1·78 (1·32, 2·41)**

Outcome: Low Birthweight (Preterms only)

	Across Pregnancy		Trimester 1		Trimester 2		Trimester 3	
**Exposure**	**N**	**OR (95% CI)**	**N**	**OR (95% CI)**	**N**	**OR (95% CI)**	**N**	**OR (95% CI)**
LMW	313	1·54 (0·94, 2·52)	45	1·29 (0·15, 11·49)	195	2·07 (0·99, 4·34)	207	1·02 (0·6, 1·75)
HMW	306	1·04 (0·53, 2·04)	37	1·03 (0·04, 24·69)	193	1·53 (0·63, 3·71)	194	0·66 (0·31, 1·38)
DEHP	308	1·02 (0·54, 1·92)	44	0·46 (0·04, 5·05)	194	1·25 (0·53, 2·92)	195	0·83 (0·42, 1·67)
PA	195	1·62 (0·87, 3·04)	34	0·73 (0·11, 5·07)	120	1·2 (0·4, 3·56)	147	1·74 (0·92, 3·29)
DiNP	210	**1·82 (1·09, 3·06)**	33	0·99 (0·17, 5·74)	138	1·21 (0·54, 2·71)	105	1·1 (0·63, 1·93)
DnOP	169	0·88 (0·39, 1·97)	33	0·34 (0·04, 3·25)	101	0·58 (0·2, 1·7)	101	0·9 (0·4, 2·01)
DiDP	264	1·51 (0·85, 2·69)	34	0·36 (0·05, 2·94)	181	1·07 (0·5, 2·29)	168	0·94 (0·52, 1·7)

Outcome: Low Birthweight (Terms only)

	Across Pregnancy		Trimester 1		Trimester 2		Trimester 3	
**Exposure**	**N**	**OR (95% CI)**	**N**	**OR (95% CI)**	**N**	**OR (95% CI)**	**N**	**OR (95% CI)**
LMW	3614	1·15 (0·72, 1·83)	664	1·47 (0·62, 3·51)	2601	1·03 (0·59, 1·8)	2603	0·99 (0·62, 1·58)
HMW	3484	1·1 (0·61, 1·98)	633	1·77 (0·65, 4·77)	2546	1·02 (0·53, 1·95)	2359	0·87 (0·47, 1·63)
DEHP	3490	1·22 (0·71, 2·1)	655	2·09 (0·84, 5·21)	2552	1·17 (0·64, 2·13)	2371	1·05 (0·6, 1·82)
PA	2051	1·51 (0·77, 2·97)	604	1·8 (0·69, 4·7)	1707	1·45 (0·74, 2·82)	1769	0·78 (0·44, 1·37)
DiNP	2318	1·05 (0·63, 1·75)	591	0·66 (0·28, 1·54)	1890	0·71 (0·41, 1·21)	1024	1·24 (0·7, 2·18)
DnOP	1636	1·19 (0·52, 2·74)	591	0·86 (0·35, 2·14)	1360	0·56 (0·24, 1·31)	899	1·66 (0·77, 3·56)
DiDP	3080	1·19 (0·68, 2·06)	604	1·34 (0·66, 2·74)	2473	0·61 (0·35, 1·04)	2123	1·56 (0·98, 2·49)

Supplement Table 8: Nominal Logistic Analysis for Adjusted Associations of Phthalate Exposures with 4 Category Gestational Age

Models adjusted for maternal age, race/ethnicity, parity, and education, and child sex·

Reference = Term Pregnancies

Exposure	N	Preterm [OR (95% CI)]	Early Term [OR (95% CI)]	Late Term [OR (95% CI)]
LMW	4389	0·91 (0·7, 1·18)	0·95 (0·81, 1·13)	1·14 (0·91, 1·42)
HMW	4446	**1·44 (1·06, 1·96)**	1·13 (0·93, 1·38)	1·2 (0·92, 1·56)
DEHP	4454	**1·35 (1·01, 1·8)**	1·18 (0·98, 1·43)	1·16 (0·9, 1·5)
PA	2607	**2·47 (1·77, 3·45)**	1·01 (0·8, 1·28)	0·79 (0·58, 1·09)
DINP	2845	**2·09 (1·63, 2·68)**	1·07 (0·9, 1·27)	0·86 (0·67, 1·1)
DNOP	2037	**3·12 (2·08, 4·68)**	0·93 (0·68, 1·28)	0·86 (0·57, 1·32)
DIDP	3775	**1·57 (1·18, 2·08)**	1·03 (0·86, 1·23)	0·97 (0·75, 1·26)

Supplement Table 9: Sensitivity Analysis for Adjusted Associations of Phthalate Exposures with Birth Outcomes, Adding Pre-Pregnancy BMI as a covariate

Models adjusted for maternal age, race/ethnicity, parity, pre-pregnancy BMI, and education, and child sex. BW for GA model removes parity and child sex from covariates.

Outcome: Gestational Age

	Across Pregnancy		Trimester 1		Trimester 2		Trimester 3	
Exposure	N	Beta (95% CI)	N	Beta (95% CI)	N	Beta (95% CI)	N	Beta (95% CI)
LMW	4167	-0·022 (-0·148, 0·104)	858	-0·107 (-0·344, 0·13)	2924	0·048 (-0·089, 0·185)	2938	-0·041 (-0·166, 0·084)
HMW	4218	**-0·186 (-0·34, -0·032)**	1021	**-0·335 (-0·592, -0·079)**	2862	0·091 (-0·07, 0·252)	2674	-0·172 (-0·346, 0·001)
DEHP	4225	**-0·169 (-0·314, -0·023)**	1050	**-0·321 (-0·569, -0·072)**	2868	0·075 (-0·077, 0·226)	2685	**-0·171 (-0·331, -0·01)**
PA	2424	**-0·71 (-0·897, -0·522)**	767	0·063 (-0·169, 0·296)	1908	-0·144 (-0·309, 0·022)	2007	**-0·553 (-0·705, -0·4)**
DiNP	2657	**-0·481 (-0·62, -0·343)**	691	0·089 (-0·126, 0·304)	2135	-0·015 (-0·144, 0·115)	1264	**-0·621 (-0·792, -0·451)**
DnOP	1854	**-0·762 (-1·034, -0·489)**	674	0·05 (-0·197, 0·297)	1503	0·016 (-0·211, 0·244)	1083	**-0·656 (-0·915, -0·397)**
DiDP	3573	**-0·303 (-0·443, -0·162)**	777	0·089 (-0·11, 0·288)	2782	0·015 (-0·118, 0·148)	2422	**-0·294 (-0·427, -0·162)**

Outcome: Birth Length

	Across Pregnancy		Trimester 1		Trimester 2		Trimester 3	
Exposure	N	Beta (95% CI)	N	Beta (95% CI)	N	Beta (95% CI)	N	Beta (95% CI)
LMW	3515	-0·106 (-0·334, 0·122)	620	**-0·637 (-1·091, -0·183)**	2561	0·027 (-0·215, 0·27)	2509	-0·058 (-0·286, 0·17)
HMW	3639	-0·136 (-0·408, 0·136)	819	-0·069 (-0·546, 0·408)	2522	0·184 (-0·096, 0·463)	2271	-0·056 (-0·368, 0·255)
DEHP	3646	-0·059 (-0·316, 0·199)	844	-0·166 (-0·623, 0·292)	2528	0·226 (-0·037, 0·488)	2282	0·016 (-0·274, 0·305)
PA	1884	**-0·991 (-1·365, -0·616)**	557	-0·028 (-0·458, 0·402)	1602	0·092 (-0·211, 0·394)	1646	**-0·803 (-1·109, -0·497)**
DiNP	2281	**-0·873 (-1·115, -0·632)**	576	0·122 (-0·259, 0·504)	1877	-0·08 (-0·306, 0·146)	945	**-1·104 (-1·427, -0·782)**
DnOP	1483	**-0·896 (-1·404, -0·387)**	557	0·052 (-0·387, 0·491)	1252	0·206 (-0·2, 0·612)	763	**-0·943 (-1·461, -0·425)**
DiDP	3037	**-0·632 (-0·899, -0·364)**	577	0·164 (-0·196, 0·525)	2467	0·107 (-0·13, 0·345)	2062	**-0·689 (-0·928, -0·449)**

Outcome: Birth Weight

	Across Pregnancy		Trimester 1		Trimester 2		Trimester 3	
Exposure	N	Beta (95% CI)	N	Beta (95% CI)	N	Beta (95% CI)	N	Beta (95% CI)
LMW	4167	-20·502 (-56·696, 15·692)	858	-37·302 (-108·258, 33·654)	2924	-17·757 (-58·558, 23·043)	2938	-14·119 (-51·205, 22·966)
HMW	4218	0·227 (-44·058, 44·512)	1021	22·059 (-55·701, 99·818)	2862	-0·758 (-48·721, 47·204)	2674	-0·816 (-51·585, 49·953)
DEHP	4225	4·978 (-36·889, 46·845)	1050	11·601 (-63·516, 86·717)	2868	3·465 (-41·757, 48·687)	2685	-3·609 (-50·734, 43·515)
PA	2424	**-148·336 (-201·348, -95·324)**	767	6·858 (-63·759, 77·474)	1908	-20·368 (-69·021, 28·285)	2007	**-122·547 (-167·59, -77·503)**
DiNP	2657	**-91·545 (-130·767, -52·322)**	691	**78·085 (13·504, 142·667)**	2135	16·363 (-21·775, 54·501)	1264	**-120·577 (-168·823, -72·33)**

	Across Pregnancy		Trimester 1		Trimester 2		Trimester 3	
Exposure	N	Beta (95% CI)	N	Beta (95% CI)	N	Beta (95% CI)	N	Beta (95% CI)
DnOP	1854	**-158·982 (-233·364, -84·601)**	674	34·354 (-39·763, 108·471)	1503	6·397 (-58·481, 71·276)	1083	**-159·831 (-231·927, -87·735)**
DiDP	3573	**-48·954 (-89·696, -8·213)**	777	40·282 (-20·28, 100·844)	2782	16·411 (-23·263, 56·085)	2422	**-61·879 (-101·409, -22·348)**

Outcome: Birthweight for Gestational Age Z-score

	Across Pregnancy		Trimester 1		Trimester 2		Trimester 3	
Exposure	N	Beta (95% CI)	N	Beta (95% CI)	N	Beta (95% CI)	N	Beta (95% CI)
LMW	3959	-0·035 (-0·11, 0·039)	688	-0·095 (-0·26, 0·07)	2733	-0·073 (-0·161, 0·015)	2743	-0·015 (-0·094, 0·064)
HMW	4009	0·089 (-0·002, 0·179)	850	0·096 (-0·08, 0·272)	2671	-0·055 (-0·156, 0·047)	2479	**0·109 (0·003, 0·214)**
DEHP	4016	**0·097 (0·012, 0·183)**	879	0·089 (-0·085, 0·263)	2677	-0·025 (-0·121, 0·071)	2490	0·096 (-0·002, 0·194)
PA	2226	-0·016 (-0·118, 0·085)	599	0·011 (-0·15, 0·173)	1721	0·023 (-0·079, 0·126)	1819	-0·022 (-0·113, 0·068)
DiNP	2457	0·009 (-0·067, 0·085)	522	**0·18 (0·032, 0·328)**	1946	0·048 (-0·032, 0·128)	1075	0·003 (-0·091, 0·096)
DnOP	1656	0 (-0·14, 0·141)	506	0·064 (-0·113, 0·242)	1316	0·008 (-0·126, 0·142)	895	-0·054 (-0·197, 0·09)
DiDP	3372	0·017 (-0·067, 0·1)	608	0·02 (-0·117, 0·157)	2592	0·026 (-0·062, 0·114)	2233	-0·023 (-0·104, 0·057)

Outcome: Preterm

	Across Pregnancy		Trimester 1		Trimester 2		Trimester 3	
Exposure	N	OR (95% CI)	N	OR (95% CI)	N	OR (95% CI)	N	OR (95% CI)
LMW	4167	0·89 (0·68, 1·16)	858	0·76 (0·39, 1·46)	2924	0·8 (0·57, 1·12)	2938	0·95 (0·72, 1·25)
HMW	4218	**1·49 (1·09, 2·03)**	1021	1·64 (0·82, 3·29)	2862	0·75 (0·51, 1·11)	2674	**1·62 (1·15, 2·29)**
DEHP	4225	**1·35 (1·01, 1·81)**	1050	1·35 (0·7, 2·61)	2868	0·74 (0·51, 1·07)	2685	**1·53 (1·12, 2·11)**
PA	2424	**2·65 (1·9, 3·7)**	767	0·79 (0·41, 1·5)	1908	1·08 (0·73, 1·59)	2007	**2·34 (1·69, 3·25)**
DiNP	2657	**2·14 (1·66, 2·75)**	691	0·76 (0·41, 1·4)	2135	0·9 (0·66, 1·22)	1264	**2·31 (1·74, 3·07)**
DnOP	1854	**3·72 (2·46, 5·62)**	674	0·86 (0·43, 1·72)	1503	1·28 (0·81, 2·03)	1083	**2·72 (1·87, 3·96)**
DiDP	3573	**1·65 (1·23, 2·19)**	777	0·7 (0·39, 1·26)	2782	0·82 (0·59, 1·12)	2422	**1·81 (1·37, 2·38)**

Outcome: Small for Gestational Age

	Across Pregnancy		Trimester 1		Trimester 2		Trimester 3	
Exposure	N	OR (95% CI)	N	OR (95% CI)	N	OR (95% CI)	N	OR (95% CI)
LMW	4160	0·96 (0·77, 1·2)	857	1·32 (0·86, 2·02)	2923	0·95 (0·73, 1·24)	2932	0·88 (0·7, 1·11)
HMW	4210	0·87 (0·66, 1·15)	1019	1·01 (0·62, 1·65)	2861	1·15 (0·85, 1·56)	2668	0·89 (0·65, 1·21)
DEHP	4217	0·87 (0·67, 1·12)	1048	0·97 (0·61, 1·56)	2867	1·11 (0·83, 1·48)	2679	0·86 (0·65, 1·15)

	Across Pregnancy		Trimester 1		Trimester 2		Trimester 3	
**Exposure**	**N**	**OR (95% CI)**	**N**	**OR (95% CI)**	**N**	**OR (95% CI)**	**N**	**OR (95% CI)**
PA	2424	0·98 (0·71, 1·33)	767	1·33 (0·85, 2·09)	1908	0·98 (0·73, 1·33)	2007	1·03 (0·78, 1·37)
DiNP	2657	1·04 (0·82, 1·33)	691	0·79 (0·52, 1·2)	2135	0·97 (0·75, 1·25)	1264	1·07 (0·81, 1·42)
DnOP	1854	1·05 (0·69, 1·59)	674	0·84 (0·53, 1·34)	1503	0·92 (0·62, 1·37)	1083	1·22 (0·82, 1·81)
DiDP	3573	0·94 (0·73, 1·22)	777	1·04 (0·72, 1·5)	2782	0·96 (0·74, 1·25)	2422	0·97 (0·77, 1·24)

Outcome: Large for Gestational Age

	Across Pregnancy		Trimester 1		Trimester 2		Trimester 3	
**Exposure**	**N**	**OR (95% CI)**	**N**	**OR (95% CI)**	**N**	**OR (95% CI)**	**N**	**OR (95% CI)**
LMW	4160	0·97 (0·77, 1·23)	857	0·85 (0·53, 1·35)	2923	0·83 (0·63, 1·08)	2932	1·09 (0·86, 1·39)
HMW	4210	1·09 (0·82, 1·44)	1019	1·54 (0·93, 2·55)	2861	0·74 (0·54, 1·01)	2668	1·12 (0·81, 1·55)
DEHP	4217	1·1 (0·84, 1·44)	1048	1·47 (0·91, 2·38)	2867	0·77 (0·57, 1·04)	2679	1·07 (0·79, 1·45)
PA	2424	1·1 (0·8, 1·49)	767	1·03 (0·66, 1·6)	1908	1·25 (0·91, 1·71)	2007	0·95 (0·73, 1·24)
DiNP	2657	0·96 (0·76, 1·22)	691	1·31 (0·89, 1·92)	2135	1·01 (0·79, 1·29)	1264	0·85 (0·64, 1·13)
DnOP	1854	0·89 (0·58, 1·38)	674	0·87 (0·55, 1·4)	1503	0·84 (0·55, 1·29)	1083	0·87 (0·59, 1·28)
DiDP	3573	1·13 (0·88, 1·45)	777	1·36 (0·93, 1·97)	2782	1·06 (0·83, 1·37)	2422	1·04 (0·8, 1·34)

Outcome: Low Birthweight

	Across Pregnancy		Trimester 1		Trimester 2		Trimester 3	
**Exposure**	**N**	**OR (95% CI)**	**N**	**OR (95% CI)**	**N**	**OR (95% CI)**	**N**	**OR (95% CI)**
LMW	4167	1·17 (0·87, 1·56)	858	1·28 (0·67, 2·44)	2924	1·11 (0·77, 1·59)	2938	0·98 (0·72, 1·32)
HMW	4218	1·39 (0·97, 1·97)	1021	1·44 (0·7, 2·97)	2862	1·02 (0·67, 1·57)	2674	1·18 (0·8, 1·74)
DEHP	4225	1·32 (0·95, 1·84)	1050	1·31 (0·67, 2·59)	2868	1·01 (0·68, 1·51)	2685	1·24 (0·87, 1·78)
PA	2424	**2·72 (1·9, 3·89)**	767	1·33 (0·67, 2·65)	1908	1·24 (0·82, 1·89)	2007	**2·03 (1·42, 2·89)**
DiNP	2657	**2·08 (1·57, 2·76)**	691	0·54 (0·26, 1·08)	2135	0·91 (0·64, 1·28)	1264	**2·14 (1·57, 2·92)**
DnOP	1854	**2·55 (1·64, 3·97)**	674	0·67 (0·31, 1·43)	1503	0·78 (0·46, 1·33)	1083	**2·16 (1·44, 3·24)**
DiDP	3573	**1·55 (1·12, 2·13)**	777	0·73 (0·41, 1·3)	2782	0·79 (0·55, 1·13)	2422	**1·62 (1·2, 2·18)**

Outcome: Low Birthweight (Preterms only)

	Across Pregnancy		Trimester 1		Trimester 2		Trimester 3	
**Exposure**	**N**	**OR (95% CI)**	**N**	**OR (95% CI)**	**N**	**OR (95% CI)**	**N**	**OR (95% CI)**
LMW	324	1·46 (0·9, 2·35)	51	0·99 (0·22, 4·47)	201	1·7 (0·88, 3·3)	220	0·93 (0·56, 1·55)
HMW	323	1·11 (0·57, 2·17)	50	0·24 (0·02, 2·96)	198	1·41 (0·58, 3·4)	208	0·89 (0·45, 1·77)
DEHP	325	1·08 (0·58, 1·99)	57	0·15 (0·02, 1·3)	199	1·35 (0·59, 3·05)	208	1·03 (0·54, 1·97)
PA	212	1·67 (0·93, 3·01)	40	1·11 (0·16, 7·76)	131	0·97 (0·38, 2·45)	163	1·78 (0·97, 3·24)
DiNP	217	**1·75 (1·08, 2·84)**			145	0·96 (0·48, 1·93)	121	1·39 (0·82, 2·35)
DnOP	177	1·01 (0·46, 2·21)			109	0·35 (0·11, 1·07)	117	1·14 (0·54, 2·41)
DiDP	279	1·35 (0·81, 2·25)	39	1·14 (0·2, 6·36)	191	0·92 (0·46, 1·82)	184	0·96 (0·56, 1·66)

Outcome: Low Birthweight (Terms only)

	Across Pregnancy		Trimester 1		Trimester 2		Trimester 3	
**Exposure**	**N**	**OR (95% CI)**	**N**	**OR (95% CI)**	**N**	**OR (95% CI)**	**N**	**OR (95% CI)**
LMW	3843	1·14 (0·72, 1·81)	807	1·7 (0·74, 3·93)	2723	1·1 (0·64, 1·91)	2718	0·97 (0·61, 1·54)
HMW	3895	1·17 (0·68, 2·03)	971	1·94 (0·82, 4·57)	2664	1·16 (0·62, 2·19)	2466	0·84 (0·45, 1·56)
DEHP	3900	1·26 (0·76, 2·09)	993	**2·25 (1·04, 4·86)**	2669	1·25 (0·7, 2·25)	2477	0·96 (0·55, 1·68)
PA	2212	1·46 (0·77, 2·76)	727	1·93 (0·78, 4·75)	1777	1·42 (0·75, 2·66)	1844	0·84 (0·48, 1·46)
DiNP	2440	1·12 (0·68, 1·85)	658	0·49 (0·2, 1·2)	1990	0·84 (0·5, 1·4)	1143	1·18 (0·7, 2·02)
DnOP	1677	1·4 (0·62, 3·16)	641	0·67 (0·26, 1·73)	1394	0·67 (0·3, 1·51)	966	1·47 (0·72, 3)
DiDP	3294	0·99 (0·58, 1·69)	738	1 (0·49, 2·05)	2591	0·69 (0·41, 1·17)	2238	1·35 (0·85, 2·16)

Supplement Table 10a1: Sensitivity Analysis for Adjusted Associations of Phthalate Exposures with Birth Outcomes (Continuously Measured), Adding Gestational Diabetes (GDM) as a Covariate

Models adjusted for maternal race/ethnicity, parity, GDM, and education, and child sex· BW for GA model removes parity from covariates·

Outcome: Gestational Age

	Across Pregnancy		Trimester 1		Trimester 2		Trimester 3	
**Exposure**	**N**	**Beta (95% CI)**	**N**	**Beta (95% CI)**	**N**	**Beta (95% CI)**	**N**	**Beta (95% CI)**
LMW	4317	-0·048 (-0·172, 0·075)	997	-0·093 (-0·298, 0·111)	3113	0·048 (-0·084, 0·179)	3036	-0·073 (-0·194, 0·048)
HMW	4388	**-0·168 (-0·319, -0·017)**	1168	**-0·317 (-0·55, -0·084)**	3054	0·113 (-0·041, 0·267)	2779	-0·158 (-0·325, 0·01)
DEHP	4396	**-0·169 (-0·311, -0·026)**	1197	**-0·295 (-0·52, -0·07)**	3061	0·082 (-0·064, 0·228)	2792	**-0·178 (-0·333, -0·022)**
PA	2596	**-0·677 (-0·857, -0·497)**	907	0·007 (-0·202, 0·216)	2073	-0·132 (-0·289, 0·026)	2137	**-0·521 (-0·667, -0·375)**
DiNP	2833	**-0·467 (-0·599, -0·334)**	832	0·051 (-0·142, 0·243)	2305	-0·025 (-0·148, 0·098)	1393	**-0·556 (-0·716, -0·396)**
DnOP	2034	**-0·672 (-0·924, -0·421)**	815	0·067 (-0·154, 0·287)	1672	-0·032 (-0·242, 0·178)	1216	**-0·556 (-0·793, -0·319)**
DiDP	3751	**-0·284 (-0·418, -0·149)**	916	0·104 (-0·072, 0·279)	2961	-0·004 (-0·127, 0·119)	2553	**-0·245 (-0·371, -0·119)**

Outcome: Birth Length

	Across Pregnancy		Trimester 1		Trimester 2		Trimester 3	
**Exposure**	**N**	**Beta (95% CI)**	**N**	**Beta (95% CI)**	**N**	**Beta (95% CI)**	**N**	**Beta (95% CI)**
LMW	3669	-0·096 (-0·32, 0·128)	754	**-0·406 (-0·785, -0·027)**	2743	0·08 (-0·151, 0·312)	2609	-0·061 (-0·283, 0·161)
HMW	3803	-0·115 (-0·382, 0·152)	959	-0·009 (-0·439, 0·42)	2704	0·215 (-0·051, 0·481)	2373	-0·047 (-0·352, 0·258)
DEHP	3811	-0·056 (-0·31, 0·198)	984	-0·039 (-0·452, 0·375)	2711	**0·254 (0·002, 0·506)**	2386	0·001 (-0·283, 0·284)
PA	2052	**-0·845 (-1·201, -0·488)**	691	-0·075 (-0·455, 0·306)	1762	0·103 (-0·183, 0·39)	1770	**-0·702 (-0·992, -0·412)**
DiNP	2445	**-0·814 (-1·047, -0·58)**	710	0·073 (-0·269, 0·416)	2038	-0·072 (-0·286, 0·143)	1065	**-0·987 (-1·289, -0·686)**
DnOP	1652	**-0·774 (-1·235, -0·313)**	691	0·092 (-0·297, 0·48)	1413	0·101 (-0·271, 0·472)	887	**-0·841 (-1·304, -0·378)**
DiDP	3209	**-0·591 (-0·843, -0·339)**	711	0·137 (-0·174, 0·448)	2640	0·056 (-0·162, 0·274)	2185	**-0·637 (-0·863, -0·411)**

Outcome: Birthweight

	Across Pregnancy		Trimester 1		Trimester 2		Trimester 3	
**Exposure**	**N**	**Beta (95% CI)**	**N**	**Beta (95% CI)**	**N**	**Beta (95% CI)**	**N**	**Beta (95% CI)**
LMW	4317	-20·115 (-56·146, 15·917)	997	-10·413 (-73·719, 52·892)	3113	-7·872 (-47·63, 31·887)	3036	-17·95 (-54·447, 18·548)
HMW	4388	10·133 (-33·9, 54·166)	1168	40·433 (-32·314, 113·18)	3054	11·019 (-35·625, 57·664)	2779	8·717 (-41·483, 58·917)
DEHP	4396	3·692 (-38·059, 45·444)	1197	29·537 (-40·708, 99·782)	3061	9·112 (-35·142, 53·366)	2792	-6·343 (-52·957, 40·271)
PA	2596	**-136·438 (-188·462, -84·413)**	907	0·579 (-64·871, 66·028)	2073	-16·077 (-63·229, 31·075)	2137	**-109·489 (-153·522, -65·456)**
DiNP	2833	**-77·989 (-116·405, -39·573)**	832	**71·867 (12·124, 131·609)**	2305	19·321 (-17·612, 56·254)	1393	**-102·698 (-148·883, -56·512)**
DnOP	2034	**-144·086 (-214·078, -74·095)**	815	24·29 (-44·25, 92·831)	1672	-1·512 (-62·762, 59·739)	1216	**-143·431 (-211·062, -75·801)**
DiDP	3751	**-51·294 (-90·906, -11·682)**	916	36·886 (-18·013, 91·785)	2961	13·913 (-23·524, 51·35)	2553	**-51·053 (-89·139, -12·967)**

Outcome: Birthweight for Gestational Age Z-Score

	Across Pregnancy		Trimester 1		Trimester 2		Trimester 3	
**Exposure**	**N**	**Beta (95% CI)**	**N**	**Beta (95% CI)**	**N**	**Beta (95% CI)**	**N**	**Beta (95% CI)**
LMW	4088	-0·024 (-0·099, 0·05)	808	-0·031 (-0·176, 0·115)	2905	-0·048 (-0·134, 0·038)	2820	-0·008 (-0·085, 0·07)
HMW	4158	**0·109 (0·02, 0·199)**	978	**0·189 (0·025, 0·354)**	2846	-0·027 (-0·125, 0·072)	2563	**0·129 (0·025, 0·233)**
DEHP	4166	**0·093 (0·008, 0·179)**	1007	**0·162 (0·001, 0·324)**	2853	-0·011 (-0·105, 0·083)	2576	0·088 (-0·009, 0·185)
PA	2377	0·001 (-0·1, 0·102)	720	0·025 (-0·125, 0·175)	1869	0·028 (-0·071, 0·127)	1928	0·001 (-0·088, 0·09)
DiNP	2612	0·046 (-0·029, 0·121)	644	**0·201 (0·063, 0·338)**	2099	0·07 (-0·008, 0·147)	1183	0·034 (-0·057, 0·125)
DnOP	1815	-0·028 (-0·161, 0·105)	628	0·024 (-0·14, 0·189)	1468	0·003 (-0·124, 0·13)	1007	-0·077 (-0·215, 0·06)
DiDP	3529	-0·007 (-0·088, 0·075)	728	0·003 (-0·121, 0·127)	2754	0·028 (-0·054, 0·111)	2343	-0·025 (-0·103, 0·053)

Supplement Table 10b1: Sensitivity Analysis for Adjusted Associations of Phthalate Exposures with Categorical Birth Outcomes, Adding Gestational Diabetes (GDM) as a Covariate

Models adjusted for maternal race/ethnicity, parity, GDM, and education, and child sex·

Outcome: Preterm

	Across Pregnancy		Trimester 1		Trimester 2		Trimester 3	
**Exposure**	**N**	**OR (95% CI)**	**N**	**OR (95% CI)**	**N**	**OR (95% CI)**	**N**	**OR (95% CI)**
LMW	4317	0·95 (0·73, 1·23)	997	0·94 (0·54, 1·64)	3113	0·79 (0·58, 1·1)	3036	1·02 (0·78, 1·34)
HMW	4388	**1·37 (1, 1·86)**	1168	1·41 (0·74, 2·7)	3054	**0·67 (0·46, 0·96)**	2779	**1·6 (1·13, 2·26)**
DEHP	4396	1·26 (0·94, 1·69)	1197	1·2 (0·65, 2·22)	3061	**0·68 (0·48, 0·97)**	2792	**1·51 (1·1, 2·07)**
PA	2596	**2·6 (1·87, 3·61)**	907	0·95 (0·52, 1·73)	2073	1·08 (0·74, 1·57)	2137	**2·33 (1·68, 3·23)**
DiNP	2833	**2·15 (1·68, 2·76)**	832	0·86 (0·49, 1·5)	2305	0·95 (0·71, 1·26)	1393	**2·28 (1·72, 3·01)**
DnOP	2034	**3·27 (2·2, 4·86)**	815	0·74 (0·39, 1·41)	1672	1·29 (0·83, 1·99)	1216	**2·62 (1·81, 3·79)**
DiDP	3751	**1·61 (1·22, 2·13)**	916	0·65 (0·39, 1·08)	2961	0·91 (0·68, 1·22)	2553	**1·71 (1·3, 2·24)**

Outcome: Small for Gestational Age

	Across Pregnancy		Trimester 1		Trimester 2		Trimester 3	
**Exposure**	**N**	**OR (95% CI)**	**N**	**OR (95% CI)**	**N**	**OR (95% CI)**	**N**	**OR (95% CI)**
LMW	4310	0·92 (0·74, 1·15)	996	1·05 (0·72, 1·53)	3112	0·91 (0·71, 1·17)	3030	0·85 (0·68, 1·06)
HMW	4380	0·86 (0·66, 1·12)	1166	0·77 (0·49, 1·22)	3053	1·16 (0·87, 1·54)	2773	0·89 (0·66, 1·2)
DEHP	4388	0·89 (0·69, 1·14)	1195	0·78 (0·51, 1·21)	3060	1·09 (0·83, 1·44)	2786	0·91 (0·69, 1·19)
PA	2596	0·95 (0·7, 1·28)	907	1·18 (0·79, 1·77)	2073	0·98 (0·74, 1·31)	2137	0·97 (0·74, 1·27)
DiNP	2833	0·95 (0·75, 1·21)	832	0·74 (0·5, 1·08)	2305	0·93 (0·74, 1·18)	1393	0·97 (0·74, 1·26)
DnOP	2034	1·06 (0·72, 1·56)	815	0·89 (0·59, 1·35)	1672	0·94 (0·65, 1·35)	1216	1·24 (0·86, 1·78)
DiDP	3751	0·95 (0·75, 1·21)	916	1·08 (0·78, 1·5)	2961	0·92 (0·72, 1·16)	2553	0·98 (0·78, 1·23)

Outcome: Large for Gestational Age

	Across Pregnancy		Trimester 1		Trimester 2		Trimester 3	
**Exposure**	**N**	**OR (95% CI)**	**N**	**OR (95% CI)**	**N**	**OR (95% CI)**	**N**	**OR (95% CI)**
LMW	4310	1·02 (0·81, 1·28)	996	1·14 (0·75, 1·73)	3112	0·87 (0·67, 1·13)	3030	1·1 (0·87, 1·39)
HMW	4380	1·14 (0·86, 1·51)	1166	**1·66 (1·03, 2·67)**	3053	0·82 (0·61, 1·12)	2773	1·12 (0·81, 1·55)
DEHP	4388	1·11 (0·85, 1·44)	1195	**1·62 (1·03, 2·54)**	3060	0·83 (0·62, 1·11)	2786	1·03 (0·76, 1·39)
PA	2596	1·15 (0·85, 1·56)	907	1·13 (0·75, 1·72)	2073	1·33 (0·98, 1·81)	2137	1 (0·77, 1·3)
DiNP	2833	1·07 (0·84, 1·35)	832	**1·48 (1·03, 2·11)**	2305	1·11 (0·88, 1·4)	1393	0·89 (0·67, 1·17)
DnOP	2034	0·97 (0·64, 1·47)	815	0·93 (0·6, 1·44)	1672	0·92 (0·62, 1·37)	1216	0·86 (0·59, 1·26)
DiDP	3751	1·11 (0·86, 1·42)	916	1·4 (0·99, 1·99)	2961	1·08 (0·85, 1·37)	2553	1·08 (0·84, 1·39)

Outcome: Low Birthweight

	Across Pregnancy		Trimester 1		Trimester 2		Trimester 3	
**Exposure**	**N**	**OR (95% CI)**	**N**	**OR (95% CI)**	**N**	**OR (95% CI)**	**N**	**OR (95% CI)**
LMW	4317	1·16 (0·87, 1·55)	997	1·08 (0·62, 1·89)	3113	0·99 (0·7, 1·4)	3036	1·03 (0·77, 1·38)
HMW	4388	1·26 (0·89, 1·79)	1168	1·35 (0·69, 2·61)	3054	0·91 (0·61, 1·36)	2779	1·09 (0·74, 1·61)
DEHP	4396	1·26 (0·91, 1·74)	1197	1·27 (0·68, 2·36)	3061	0·93 (0·63, 1·35)	2792	1·22 (0·86, 1·74)
PA	2596	**2·57 (1·81, 3·65)**	907	1·27 (0·69, 2·34)	2073	1·21 (0·81, 1·81)	2137	**1·96 (1·38, 2·77)**
DiNP	2833	**1·92 (1·46, 2·54)**	832	0·72 (0·4, 1·3)	2305	0·92 (0·66, 1·27)	1393	**1·88 (1·39, 2·53)**
DnOP	2034	**2·26 (1·48, 3·45)**	815	0·71 (0·37, 1·36)	1672	0·88 (0·54, 1·42)	1216	**2·04 (1·38, 3·01)**
DiDP	3751	**1·52 (1·12, 2·07)**	916	0·86 (0·53, 1·39)	2961	0·86 (0·63, 1·19)	2553	**1·5 (1·12, 2)**

Outcome: Low Birthweight (Preterms only)

	Across Pregnancy		Trimester 1		Trimester 2		Trimester 3	
**Exposure**	**N**	**OR (95% CI)**	**N**	**OR (95% CI)**	**N**	**OR (95% CI)**	**N**	**OR (95% CI)**
LMW	338	1·39 (0·87, 2·23)	60	1·34 (0·37, 4·81)	217	1·45 (0·76, 2·74)	224	0·97 (0·59, 1·6)
HMW	338	1·05 (0·56, 1·97)	59	0·21 (0·03, 1·57)	214	1·39 (0·61, 3·14)	212	0·73 (0·37, 1·46)
DEHP	340	1·04 (0·58, 1·89)	66	0·19 (0·04, 1·02)	215	1·36 (0·62, 3)	213	0·91 (0·48, 1·74)
PA	224	1·55 (0·88, 2·74)	49	1·19 (0·3, 4·82)	142	0·81 (0·33, 2·02)	168	1·68 (0·94, 2·98)
DiNP	232	**1·64 (1·02, 2·63)**	42	0·84 (0·17, 4·08)	158	0·91 (0·46, 1·79)	128	1·25 (0·76, 2·07)
DnOP	191	0·96 (0·46, 2·01)	42	0·88 (0·18, 4·39)	122	0·51 (0·2, 1·32)	123	1·16 (0·56, 2·38)
DiDP	292	1·29 (0·79, 2·12)	48	0·94 (0·27, 3·26)	203	0·91 (0·48, 1·72)	189	0·89 (0·52, 1·51)

Outcome: Low Birthweight (Terms only)

	Across Pregnancy		Trimester 1		Trimester 2		Trimester 3	
**Exposure**	**N**	**OR (95% CI)**	**N**	**OR (95% CI)**	**N**	**OR (95% CI)**	**N**	**OR (95% CI)**
LMW	3979	1·11 (0·71, 1·74)	937	1·17 (0·55, 2·46)	2896	0·95 (0·56, 1·62)	2812	0·99 (0·63, 1·55)
HMW	4050	1·09 (0·64, 1·88)	1109	1·76 (0·78, 3·99)	2840	1·04 (0·57, 1·91)	2567	0·79 (0·43, 1·47)
DEHP	4056	1·25 (0·76, 2·05)	1131	**2·16 (1·03, 4·5)**	2846	1·13 (0·64, 1·98)	2579	1 (0·58, 1·72)
PA	2372	1·42 (0·76, 2·64)	858	1·58 (0·7, 3·54)	1931	1·46 (0·8, 2·68)	1969	0·8 (0·47, 1·38)
DiNP	2601	0·94 (0·57, 1·55)	790	0·64 (0·3, 1·4)	2147	0·83 (0·51, 1·34)	1265	0·93 (0·55, 1·57)
DnOP	1843	1·18 (0·54, 2·56)	773	0·66 (0·28, 1·52)	1550	0·72 (0·34, 1·53)	1093	1·31 (0·67, 2·57)
DiDP	3459	1·02 (0·61, 1·69)	868	1·13 (0·6, 2·15)	2758	0·71 (0·44, 1·14)	2364	1·28 (0·82, 2·01)

Supplement Table 10a2: Sensitivity Analysis for Adjusted Associations of Phthalate Exposures with Birth Outcomes (Continuously Measured), Adding Gestational Hypertension (GHTN) as a Covariate

Models adjusted for maternal race/ethnicity, parity, GHTN, and education, and child sex· BW for GA model removes parity from covariates·

Outcome: Gestational Age

	Across Pregnancy		Trimester 1		Trimester 2		Trimester 3	
Exposure	N	Beta (95% CI)	N	Beta (95% CI)	N	Beta (95% CI)	N	Beta (95% CI)
LMW	2099	**-0·29 (-0·476, -0·104)**	547	-0·085 (-0·365, 0·196)	1612	0·034 (-0·145, 0·212)	1117	**-0·399 (-0·62, -0·179)**
HMW	2232	**-0·51 (-0·738, -0·282)**	749	**-0·355 (-0·639, -0·071)**	1582	0·015 (-0·204, 0·234)	1046	**-0·625 (-0·943, -0·307)**
DEHP	2240	**-0·48 (-0·708, -0·252)**	778	**-0·321 (-0·603, -0·038)**	1587	0·049 (-0·173, 0·27)	1051	**-0·582 (-0·896, -0·267)**
PA	895	**-1·205 (-1·493, -0·918)**	477	0·138 (-0·135, 0·411)	712	-0·038 (-0·271, 0·195)	698	**-0·958 (-1·188, -0·727)**
DiNP	1504	**-0·558 (-0·723, -0·394)**	477	0·059 (-0·188, 0·305)	1169	-0·117 (-0·269, 0·035)	850	**-0·679 (-0·881, -0·477)**
DnOP	895	**-1·036 (-1·376, -0·695)**	477	0·072 (-0·214, 0·358)	712	-0·181 (-0·445, 0·083)	698	**-1·001 (-1·324, -0·677)**
DiDP	1998	**-0·265 (-0·455, -0·075)**	478	0·098 (-0·123, 0·319)	1540	-0·073 (-0·229, 0·082)	1075	**-0·311 (-0·529, -0·093)**

Outcome: Birth Length

	Across Pregnancy		Trimester 1		Trimester 2		Trimester 3	
Exposure	N	Beta (95% CI)	N	Beta (95% CI)	N	Beta (95% CI)	N	Beta (95% CI)
LMW	1778	**-0·444 (-0·789, -0·1)**	473	-0·13 (-0·608, 0·348)	1413	-0·056 (-0·389, 0·278)	865	**-0·604 (-1·036, -0·172)**
HMW	1910	**-0·907 (-1·331, -0·483)**	676	-0·022 (-0·534, 0·49)	1384	-0·281 (-0·691, 0·129)	795	**-0·893 (-1·524, -0·261)**
DEHP	1918	**-0·837 (-1·262, -0·411)**	701	0·004 (-0·499, 0·507)	1389	-0·118 (-0·528, 0·293)	800	**-0·722 (-1·355, -0·088)**
PA	628	**-1·948 (-2·586, -1·31)**	430	0·19 (-0·288, 0·669)	556	0·065 (-0·437, 0·566)	459	**-1·65 (-2·198, -1·101)**
DiNP	1232	**-1·172 (-1·476, -0·869)**	430	0·045 (-0·404, 0·494)	1009	**-0·407 (-0·697, -0·116)**	610	**-1·176 (-1·564, -0·788)**
DnOP	628	**-1·209 (-1·907, -0·51)**	430	0·195 (-0·294, 0·684)	556	-0·206 (-0·727, 0·314)	459	**-1·435 (-2·128, -0·741)**
DiDP	1710	**-0·575 (-0·917, -0·233)**	431	0·124 (-0·246, 0·493)	1366	-0·237 (-0·522, 0·048)	832	**-0·612 (-1·012, -0·211)**

Outcome: Birthweight

	Across Pregnancy		Trimester 1		Trimester 2		Trimester 3	
**Exposure**	**N**	**Beta (95% CI)**	**N**	**Beta (95% CI)**	**N**	**Beta (95% CI)**	**N**	**Beta (95% CI)**
LMW	2099	**-92·516 (-146·272, -38·759)**	547	-23·618 (-109·607, 62·37)	1612	-18·343 (-73·792, 37·106)	1117	**-111·571 (-175·084, -48·059)**
HMW	2232	-49·563 (-115·604, 16·478)	749	67·498 (-20·868, 155·864)	1582	-21·033 (-89·268, 47·202)	1046	-90·942 (-182·564, 0·681)
DEHP	2240	-48·724 (-114·78, 17·332)	778	70·098 (-17·441, 157·637)	1587	-12·571 (-81·409, 56·268)	1051	**-95·314 (-185·828, -4·8)**
PA	895	**-227·67 (-308·796, -146·545)**	477	57·871 (-27·484, 143·227)	712	-5·993 (-79·481, 67·495)	698	**-170·102 (-236·166, -104·037)**
DiNP	1504	**-124·694 (-172·026, -77·362)**	477	**85·426 (8·528, 162·325)**	1169	-15·791 (-64·182, 32·6)	850	**-152·103 (-208·58, -95·626)**
DnOP	895	**-191·588 (-286·751, -96·425)**	477	67·208 (-22·104, 156·519)	712	-38·871 (-122·175, 44·434)	698	**-212·225 (-303·289, -121·16)**
DiDP	1998	**-68·974 (-124·101, -13·848)**	478	36·147 (-33·032, 105·325)	1540	-15·109 (-64·125, 33·906)	1075	**-66·104 (-128·093, -4·116)**

Outcome: Birthweight for Gestational Age Z-Score

	Across Pregnancy		Trimester 1		Trimester 2		Trimester 3	
**Exposure**	**N**	**Beta (95% CI)**	**N**	**Beta (95% CI)**	**N**	**Beta (95% CI)**	**N**	**Beta (95% CI)**
LMW	2097	-0·091 (-0·198, 0·015)	546	-0·057 (-0·234, 0·121)	1610	-0·047 (-0·167, 0·073)	1117	-0·098 (-0·215, 0·019)
HMW	2229	0·101 (-0·029, 0·232)	747	**0·3 (0·118, 0·482)**	1580	-0·05 (-0·196, 0·097)	1046	0·084 (-0·08, 0·248)
DEHP	2237	0·088 (-0·043, 0·219)	776	**0·29 (0·108, 0·471)**	1585	-0·045 (-0·193, 0·103)	1051	0·047 (-0·118, 0·211)
PA	895	0·045 (-0·095, 0·185)	477	0·047 (-0·126, 0·22)	712	0 (-0·147, 0·147)	698	0·054 (-0·058, 0·165)
DiNP	1504	-0·016 (-0·105, 0·073)	477	**0·229 (0·075, 0·383)**	1169	0·035 (-0·066, 0·137)	850	-0·022 (-0·122, 0·077)
DnOP	895	0·013 (-0·15, 0·176)	477	0·085 (-0·097, 0·267)	712	0·001 (-0·166, 0·167)	698	-0·046 (-0·2, 0·108)
DiDP	1997	-0·041 (-0·149, 0·067)	478	0·042 (-0·098, 0·182)	1539	0·015 (-0·09, 0·12)	1075	-0·014 (-0·126, 0·097)

Supplement Table 10b2: Sensitivity Analysis for Adjusted Associations of Phthalate Exposures with Categorical Birth Outcomes, Adding Gestational Hypertension (GHTN) as a Covariate

Models adjusted for maternal race/ethnicity, parity, GHTN, and education, and child sex·

Outcome: Preterm

	Across Pregnancy		Trimester 1		Trimester 2		Trimester 3	
**Exposure**	**N**	**OR (95% CI)**	**N**	**OR (95% CI)**	**N**	**OR (95% CI)**	**N**	**OR (95% CI)**
LMW	2099	1·23 (0·86, 1·75)	547	0·99 (0·46, 2·13)	1612	0·86 (0·55, 1·36)	1117	1·41 (0·97, 2·07)
HMW	2232	**2·06 (1·34, 3·15)**	749	1·32 (0·59, 2·92)	1582	0·88 (0·5, 1·56)	1046	**2·73 (1·63, 4·59)**
DEHP	2240	**1·92 (1·26, 2·9)**	778	1·08 (0·5, 2·31)	1587	0·84 (0·47, 1·5)	1051	**2·51 (1·53, 4·13)**
PA	895	**5·28 (3·35, 8·35)**	477	0·82 (0·38, 1·78)	712	1·48 (0·74, 2·94)	698	**4·71 (2·98, 7·43)**
DiNP	1504	**2·95 (2·19, 3·99)**	477	1·12 (0·56, 2·24)	1169	**1·64 (1·11, 2·42)**	850	**3·6 (2·5, 5·19)**
DnOP	895	**5·32 (3·17, 8·94)**	477	0·74 (0·31, 1·77)	712	**2·32 (1·2, 4·5)**	698	**5·37 (3·19, 9·03)**
DiDP	1998	**2·25 (1·55, 3·25)**	478	0·63 (0·33, 1·21)	1540	**1·53 (1·02, 2·31)**	1075	**2·13 (1·43, 3·18)**

Outcome: Small for Gestational Age

	Across Pregnancy		Trimester 1		Trimester 2		Trimester 3	
**Exposure**	**N**	**OR (95% CI)**	**N**	**OR (95% CI)**	**N**	**OR (95% CI)**	**N**	**OR (95% CI)**
LMW	2098	1·16 (0·84, 1·6)	546	1·1 (0·67, 1·83)	1611	1·04 (0·71, 1·51)	1117	1·02 (0·71, 1·45)
HMW	2230	1·09 (0·73, 1·64)	747	0·68 (0·39, 1·18)	1581	1·42 (0·9, 2·24)	1046	1·37 (0·84, 2·24)
DEHP	2238	1·11 (0·74, 1·67)	776	0·6 (0·35, 1·03)	1586	1·37 (0·86, 2·16)	1051	1·28 (0·79, 2·07)
PA	895	1·19 (0·75, 1·88)	477	1·42 (0·82, 2·45)	712	1·24 (0·74, 2·07)	698	0·95 (0·65, 1·4)
DiNP	1504	1·19 (0·89, 1·59)	477	0·69 (0·42, 1·15)	1169	0·93 (0·65, 1·32)	850	1·22 (0·89, 1·68)
DnOP	895	1·18 (0·69, 2·02)	477	1·05 (0·61, 1·8)	712	1·04 (0·6, 1·81)	698	1·18 (0·71, 1·94)
DiDP	1998	1·25 (0·89, 1·76)	478	1·23 (0·8, 1·88)	1540	0·88 (0·63, 1·23)	1075	1·15 (0·82, 1·62)

Outcome: Large for Gestational Age

	Across Pregnancy		Trimester 1		Trimester 2		Trimester 3	
**Exposure**	**N**	**OR (95% CI)**	**N**	**OR (95% CI)**	**N**	**OR (95% CI)**	**N**	**OR (95% CI)**
LMW	2098	0·92 (0·65, 1·31)	546	1·92 (0·95, 3·89)	1611	0·87 (0·59, 1·27)	1117	1·04 (0·69, 1·56)
HMW	2230	1·2 (0·8, 1·81)	747	**2·16 (1·12, 4·16)**	1581	0·83 (0·53, 1·32)	1046	1·15 (0·65, 2·05)
DEHP	2238	1·23 (0·82, 1·85)	776	**2·35 (1·23, 4·47)**	1586	0·82 (0·51, 1·32)	1051	1·04 (0·59, 1·83)
PA	895	1·3 (0·83, 2·03)	477	**2·62 (1·27, 5·42)**	712	**1·73 (1·03, 2·89)**	698	1·01 (0·71, 1·44)
DiNP	1504	0·96 (0·72, 1·28)	477	**2·33 (1·37, 3·95)**	1169	1·08 (0·79, 1·47)	850	0·91 (0·63, 1·32)
DnOP	895	1·14 (0·66, 1·95)	477	**2·02 (1·07, 3·82)**	712	1 (0·57, 1·77)	698	0·86 (0·5, 1·45)
DiDP	1998	1·08 (0·76, 1·53)	478	**1·89 (1·11, 3·22)**	1540	1·09 (0·78, 1·52)	1075	1·11 (0·73, 1·69)

Outcome: Low Birthweight

	Across Pregnancy		Trimester 1		Trimester 2		Trimester 3	
**Exposure**	**N**	**OR (95% CI)**	**N**	**OR (95% CI)**	**N**	**OR (95% CI)**	**N**	**OR (95% CI)**
LMW	2099	**1·55 (1·06, 2·27)**	547	1·13 (0·59, 2·17)	1612	1·11 (0·68, 1·82)	1117	1·41 (0·96, 2·07)
HMW	2232	**1·77 (1·09, 2·88)**	749	1·37 (0·67, 2·81)	1582	1·36 (0·73, 2·52)	1046	1·55 (0·91, 2·65)
DEHP	2240	**1·72 (1·08, 2·76)**	778	1·34 (0·68, 2·63)	1587	1·3 (0·7, 2·42)	1051	1·63 (0·97, 2·74)
PA	895	**3·96 (2·52, 6·22)**	477	1·29 (0·63, 2·65)	712	1·48 (0·77, 2·86)	698	**2·81 (1·85, 4·26)**
DiNP	1504	**2·18 (1·57, 3·04)**	477	0·91 (0·48, 1·7)	1169	1·27 (0·81, 2)	850	**2·21 (1·56, 3·13)**
DnOP	895	**3·02 (1·8, 5·06)**	477	0·81 (0·39, 1·68)	712	1·49 (0·77, 2·85)	698	**2·75 (1·69, 4·47)**
DiDP	1998	**1·8 (1·2, 2·7)**	478	1·04 (0·6, 1·81)	1540	1·34 (0·87, 2·08)	1075	1·35 (0·91, 2)

Outcome: Low Birthweight (Preterms only)

	Across Pregnancy		Trimester 1		Trimester 2		Trimester 3	
**Exposure**	**N**	**OR (95% CI)**	**N**	**OR (95% CI)**	**N**	**OR (95% CI)**	**N**	**OR (95% CI)**
LMW	181	**1·91 (1, 3·64)**			111	2·08 (0·63, 6·81)	103	1·26 (0·6, 2·64)
HMW	185	0·72 (0·28, 1·83)			109	2·81 (0·42, 18·68)	100	**0·25 (0·07, 0·84)**
DEHP	187	0·66 (0·26, 1·66)			110	1·96 (0·3, 13)	101	0·32 (0·1, 1·03)
PA	92	1·33 (0·59, 3)					73	1·39 (0·61, 3·14)
DiNP	130	1·71 (0·88, 3·32)			73	4·27 (0·92, 19·77)	79	0·78 (0·35, 1·73)
DnOP	92	1·01 (0·34, 2·99)					73	0·37 (0·1, 1·36)
DiDP	161	1·25 (0·58, 2·73)			100	2·11 (0·62, 7·17)	93	0·39 (0·15, 1·04)

Outcome: Low Birthweight (Terms only)

	Across Pregnancy		Trimester 1		Trimester 2		Trimester 3	
**Exposure**	**N**	**OR (95% CI)**	**N**	**OR (95% CI)**	**N**	**OR (95% CI)**	**N**	**OR (95% CI)**
LMW	1918	1·24 (0·67, 2·29)	512	1·22 (0·51, 2·89)	1501	0·99 (0·49, 2)	1014	1·24 (0·68, 2·28)
HMW	2047	2·02 (0·97, 4·23)	712	1·7 (0·69, 4·21)	1473	1·71 (0·73, 3·98)	946	1·6 (0·71, 3·63)
DEHP	2053	**2·32 (1·16, 4·66)**	734	2·14 (0·93, 4·89)	1477	1·79 (0·77, 4·13)	950	1·96 (0·92, 4·17)
PA	803	1·81 (0·78, 4·18)	453	1·59 (0·61, 4·15)	671	1·64 (0·7, 3·82)	625	0·94 (0·49, 1·82)
DiNP	1374	0·95 (0·53, 1·7)	453	0·68 (0·28, 1·64)	1096	0·84 (0·44, 1·61)	771	1·09 (0·61, 1·95)
DnOP	803	1·35 (0·53, 3·41)	453	0·92 (0·36, 2·35)	671	0·9 (0·35, 2·31)	625	1·74 (0·77, 3·95)
DiDP	1837	1·24 (0·64, 2·38)	454	1·41 (0·67, 2·95)	1440	0·91 (0·49, 1·7)	982	1·11 (0·59, 2·1)

Supplement Figure 1: Depicting Correlations Among Measured Phthalates

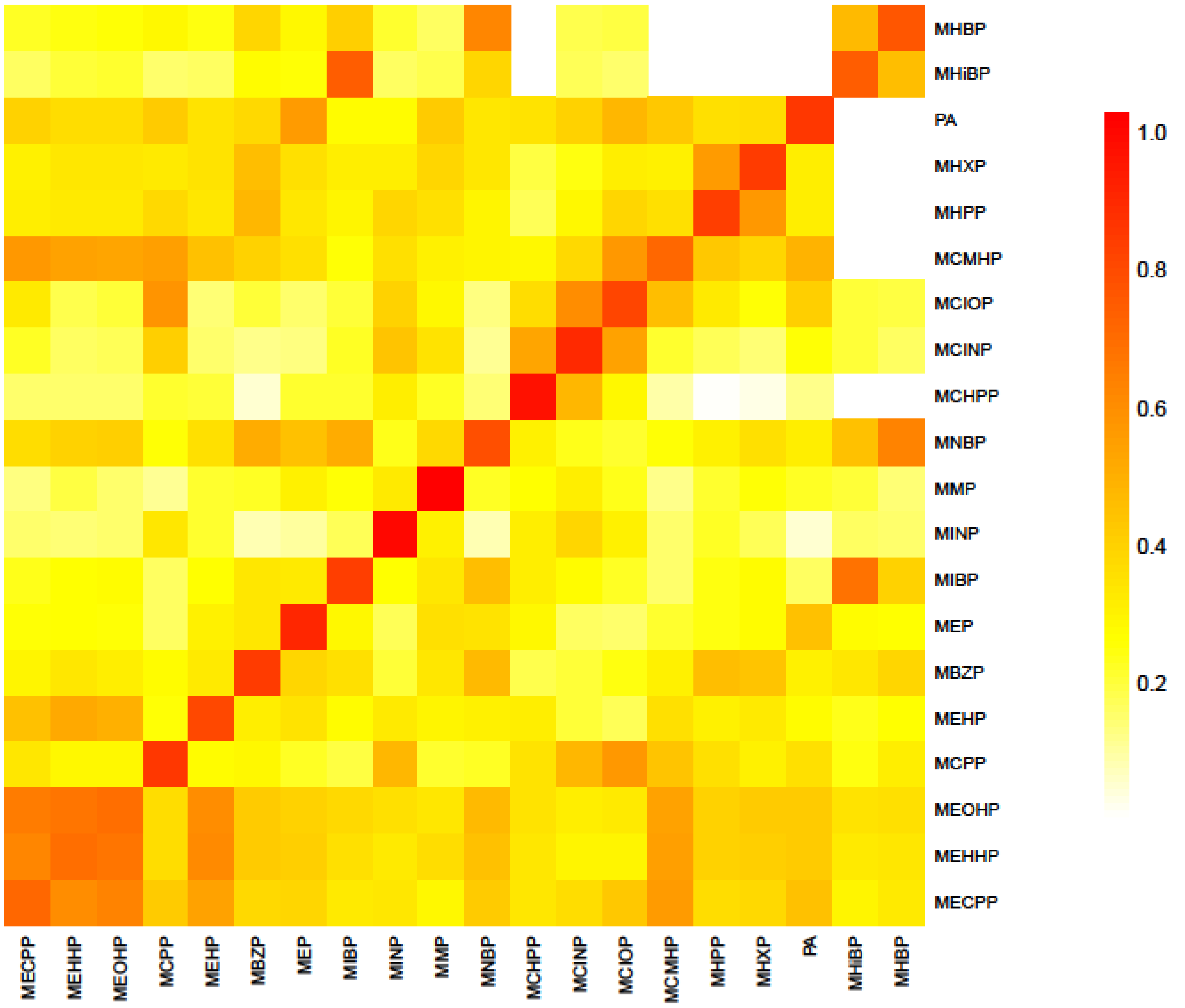

Supplement Figure 2: Distribution of Individual Analytes Across Pregnancy

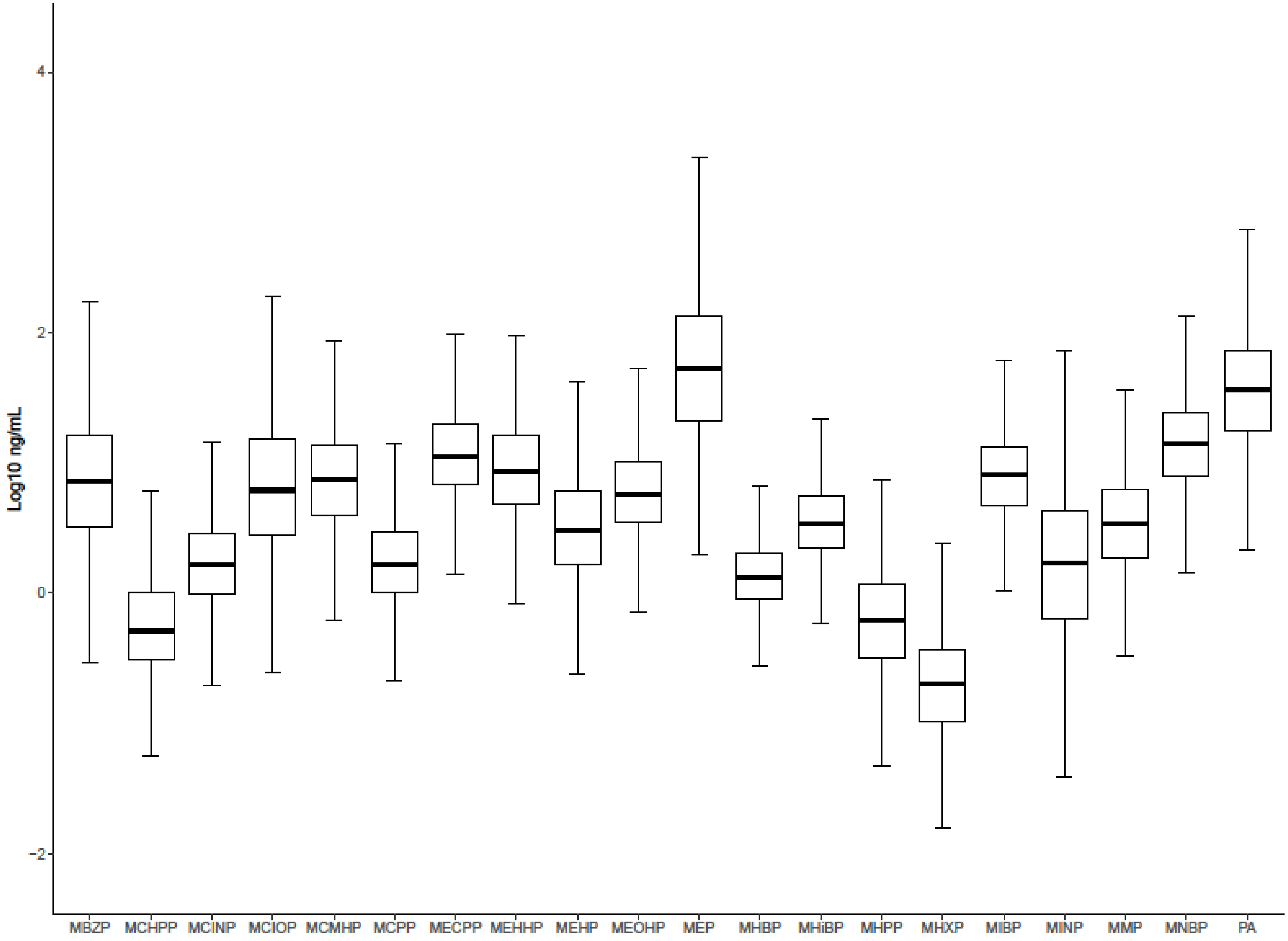

Figure 3a: Leave-One-Out Analysis: Birth Gestational Age.

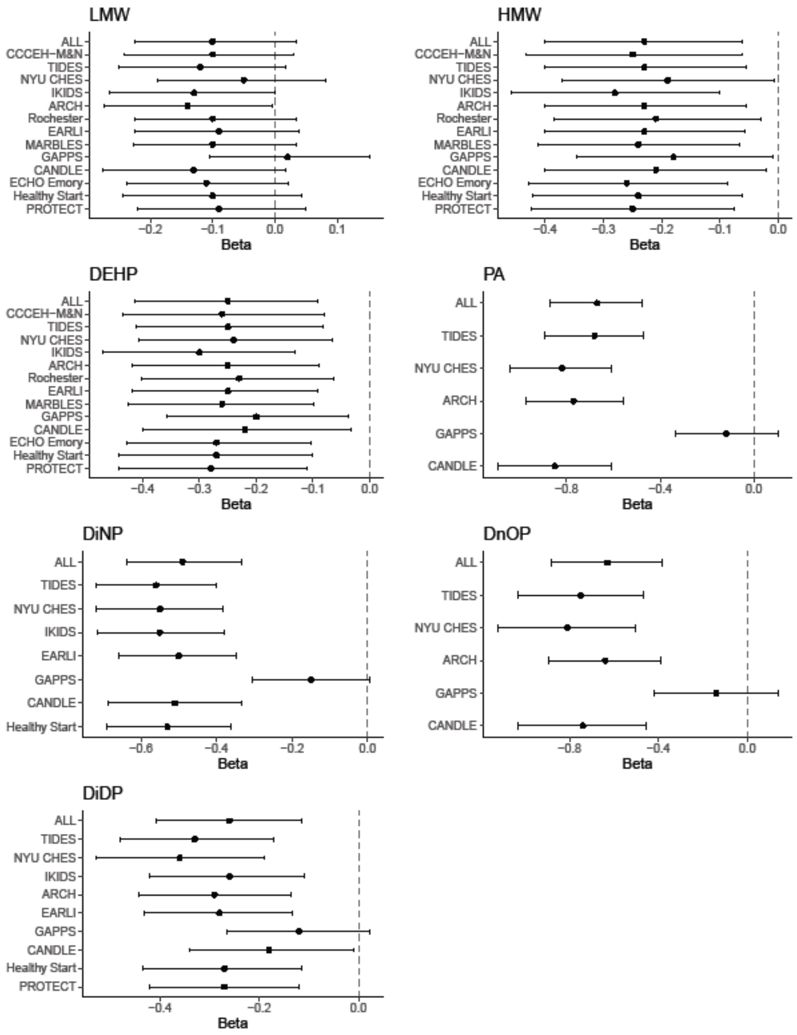

When a cohort is named in a row, it is the cohort excluded in the leave-one-out analysis.

<u>Figure 3b: Leave-One-Out Analysis: Preterm Birth.</u>

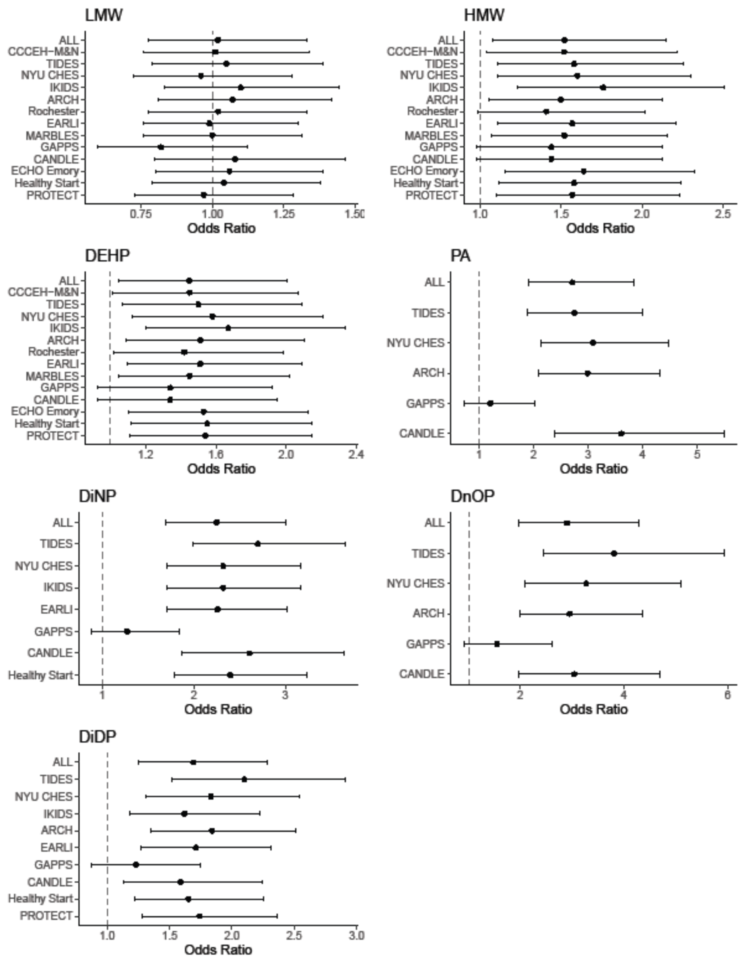

When a cohort is named in a row, it is the cohort excluded in the leave-one-out analysis.
